# Differential lipid selectivity of StARD phospholipid transporters revealed by native MS

**DOI:** 10.1038/s41467-026-76988-1

**Published:** 2026-08-22

**Authors:** Carla Kirschbaum, Sophie A. S. Lawrence, Jack L. Bennett, Olivia B. Ramsay, Yara Ahmed, Tarick J. El-Baba, Stefano Vanni, Carol V. Robinson

**Affiliations:** 1 Kavli Institute for Nanoscience Discovery, University of Oxford, Oxford, UK; 2 Department of Chemistry, University of Oxford, Oxford, UK; 3 Department of Oncology, University of Oxford, Oxford, UK; 4 Department of Biology, University of Fribourg, Fribourg, Switzerland; 5 Swiss National Center for Competence in Research Bio-inspired Materials, University of Fribourg, Fribourg, Switzerland; 6 Present Address: Institute of Biochemistry, Charité – University Medicine Berlin, Berlin, Germany

**Keywords:** Membrane lipids, Mass spectrometry, Phosphorylation, Molecular modelling, Proteomic analysis

## Abstract

Intracellular lipid transport in eukaryotes is largely mediated by lipid transfer proteins (LTPs). Transport kinetics differ markedly among lipid species, implying selective lipid recognition by the involved proteins. Here, we characterize endogenous ligands of the human phospholipid transporters STARD2, STARD7, and STARD10 by multistage native mass spectrometry (MS). Our results demonstrate that they exhibit distinct lipid selectivities, with STARD7 binding a broad range of phospholipids, whereas STARD2 and STARD10 preferentially copurify with poly- and di-unsaturated phospholipids, respectively. We link this acyl chain selectivity to tissue-specific LTP expression patterns and show that LTP expression levels modulate lipid metabolism. Through site-directed mutagenesis and molecular dynamics simulations, we further identify a conserved arginine that is essential for phospholipid binding in STARD7 but dispensable in STARD2 and STARD10. To investigate regulation of LTP activity, we mapped phosphorylation sites by native top-down MS and found that STARD2 and STARD10 are phosphorylated in membrane-binding regions. Liposome-based assays revealed that phosphorylation abolishes lipid transfer activity of STARD10 and that lipid selectivity influences the transfer rates of different lipid probes. Together, our results demonstrate that LTPs exhibit distinct lipid binding preferences and suggest that cells finely tune lipid homeostasis by regulating LTP expression levels and activity.

## Abstract (web summary)

Lipid transfer proteins organize intracellular lipid distribution. Here, the authors show that seemingly redundant phospholipid transfer proteins have distinct acyl chain preferences, and suggest that their tissue-specific expression levels and phosphorylation fine-tune membrane composition.

## Introduction

Eukaryotic membranes are defined by unique lipid compositions tailored to support organelle-specific functions^1^. To maintain distinct lipid profiles across cells, newly synthesized lipids must be selectively transported from their site of synthesis, typically the endoplasmic reticulum, to their target organelle or the plasma membrane^2^. The majority of intracellular lipid transfer is mediated by lipid transfer proteins (LTPs) at membrane contact sites^3–5^. Through their lipid selectivity, LTPs fine-tune organelle membrane composition, including the spatial distribution of signaling lipids^6,7^.

The steroidogenic acute regulatory protein-related (StAR) family comprises 15 soluble and membrane-associated LTPs^8,9^, three of which—STARD2, STARD7, and STARD10—transfer phospholipids^10–12^. These proteins are known to transport phosphatidylcholine (PC), which in STARD2 and STARD7 is stabilized through interactions between the choline headgroup and an aromatic cage in the binding pocket^13,14^. STARD10 lacks this aromatic cage and can accommodate a broader spectrum of phospholipids, most notably phosphatidylethanolamine (PE) and phosphatidylglycerol (PG)^15,16^. Although all three proteins are primarily cytosolic, STARD7 is initially targeted to mitochondria and also localizes to the mitochondrial intermembrane space^17–19^.

Despite these differences in structure and localization, the functional specialization of STARD2, STARD7, and STARD10 remains incompletely understood. Biochemical assays have demonstrated that STARD2 transports PC lipids at greatly varying rates depending on their acyl chain composition, suggesting that lipid selectivity may extend beyond headgroup recognition^20^. However, whether STARD2, STARD7, and STARD10 exhibit distinct acyl chain selectivities, or are largely redundant, remains unclear. Equally unresolved is how lipid transfer activity is regulated. For STARD2, it is thought that two alpha-helices near the C-terminus mediate membrane association^21,22^, with phosphorylation initiating relocation to mitochondria^23^. In STARD10, phosphorylation of the C-terminal tail is thought to regulate transport activity^24–26^. The direct effects of these modifications on lipid binding and transfer have not been defined.

A central challenge in understanding LTP function is to distinguish what they can bind in vitro from what they actually carry in cells. Most studies have relied on in vitro reconstitution, incubating purified proteins with lipids to assess binding capacity^27,28^. Although such approaches have been instrumental in defining the lipid-binding preferences of STARD2^20,29–31^, STARD7^11,14^, and STARD10^15,32^, they cannot capture binding selectivity within the spatially regulated cellular lipidome, where hundreds of lipid species compete for interaction. Similarly, while bifunctional and fluorescent lipids have proven powerful for studying protein–lipid interactions and lipid transport in cells^12,33^, currently available lipid probes represent only a fraction of the human lipidome^34^. To characterize endogenous protein–lipid interactions, LTPs have been affinity-purified from cells or tissues for analysis of copurified lipids using thin-layer chromatography or mass spectrometry (MS)^35^, as exemplified for STARD2 and STARD10^16,36^. However, studies addressing how acyl chains influence protein–lipid interactions are rare^27^. This represents a critical gap: acyl chain unsaturation significantly influences phospholipid transport kinetics and metabolic fate^37^, yet the endogenous lipid cargo of individual LTPs remains undefined at this level of resolution.

Here, we establish a multistage native MS workflow to directly identify endogenous ligands and regulatory post-translational modifications (PTMs) of human LTPs. We characterize lipid cargo with acyl chain resolution by purifying STARD2, STARD7, and STARD10 from their native environment, separating intact protein–lipid complexes in the gas phase, and releasing endogenous ligands for identification. We uncover distinct and non-redundant acyl chain selectivities among these phospholipid transfer proteins that align with tissue-specific expression patterns and lipid metabolic profiles. Integrating ligand profiling with top-down phosphorylation mapping and targeted mutation of the lipid-binding pocket, we define molecular mechanisms that regulate lipid selectivity and transfer. Together, our findings reveal a previously unrecognized alignment between acyl chain–selective phospholipid transport, LTP regulation, and cellular lipid metabolism.

## Results

### Headgroup and acyl chain-selective phospholipid binding in vitro

**Fig. 1:**
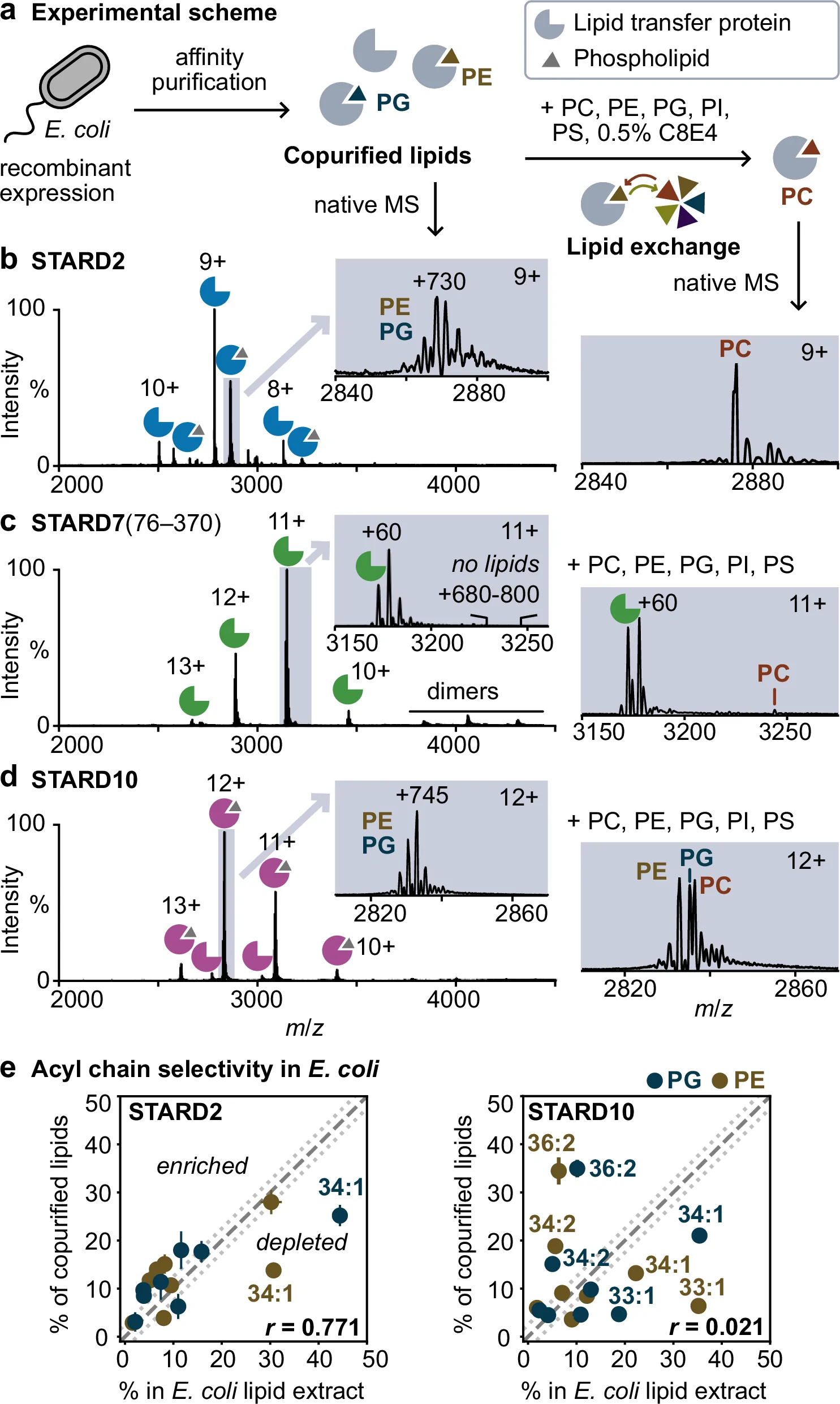
Lipid binding preferences of phospholipid transfer proteins in vitro. **a** Experimental scheme showing LTP overexpression in *E. coli*, followed by affinity purification and identification of copurified bacterial PE (beige) and PG (blue). To determine lipid class binding preferences, purified LTPs are incubated with an equimolar mixture of DOPC, DOPE, DOPG, DOPI, and DOPS in detergent micelles containing 0.5% C8E4 (1:5 protein:lipid ratio), resulting in replacement of copurified lipids by the preferred lipid ligands (here exemplarily shown for a selective PC transfer protein). **b**–**d** Native mass spectra of STARD2 (blue), STARD7(76–370) (green), and STARD10 (purple) recombinantly expressed in *E. coli*. Insets in the mass spectra of STARD2 (**b**) and STARD10 (**d**) show peaks assigned as copurified bacterial PE and PG. The mass shift relative to the *apo* protein is labeled for the most intense peak. The native mass spectrum of STARD7(76–370) (**c**) shows that it is purified in its lipid-free *apo* form and binds acetate in a 1:1 stoichiometry (+60 Da). Native mass spectra on the right are shown after incubation with the mixed lipid micelles. STARD2 and STARD7(76–370) bind exclusively to DOPC (orange), whereas STARD10 binds DOPC, DOPE, and DOPG in similar amounts. **e** Correlation plots showing the relative abundances of lipids copurified with STARD2 and STARD10 vs their relative abundances in the bacterial cell pellets. PE and PG species were quantified individually. Lipids copurified with STARD2 show a high correlation with the cellular lipidome (Pearson’s *r* = 0.771), whereas lipids bound to STARD10 are enriched in di-unsaturated lipids (34:2 and 36:2) and only weakly correlated with the cellular lipid composition (Pearson’s *r* = 0.021). Data points represent the mean of three technical replicates, and error bars indicate the standard deviation. The dotted light gray lines indicate ±2.5% deviation from the diagonal. Source data are provided as a Source Data file.

To investigate the intrinsic phospholipid-binding preferences of STARD2, STARD7, and STARD10, we recombinantly expressed full-length STARD2 and STARD10, as well as a truncated STARD7 construct (residues 76–370) to account for cleavage of the mitochondrial localization signal, in *Escherichia coli* (Supplementary Fig. 1, Supplementary Table 1). The purified proteins were analyzed by native MS following buffer exchange into 200 mM ammonium acetate (Fig. 1a). The native mass spectrum of STARD2 featured a single charge state distribution (*z* = 8–10 + ) comprising two species: (i) ca. 30% *apo* STARD2, and (ii) 70% STARD2 bound to an ensemble of ligands with an average molecular weight of 730 Da at a 1:1 ratio (Fig. 1b). To characterize the copurified ligands, we isolated the protein–ligand complexes (*z* = 9+) in the ion trap of the mass spectrometer and activated them using higher-energy collisional dissociation (HCD) to release bound molecules for high-resolution mass analysis^38^. We subsequently assigned the released ions based on accurate mass and HCD fragmentation patterns. *E. coli* membranes predominantly contain PE and PG but lack PC, the endogenous ligand of STARD2^39^. Accordingly, the copurified lipids were identified as PE and PG species (Supplementary Fig. 2a), both of which have previously been shown to bind STARD2 in the absence of PC^31^.

In contrast, for STARD7(76–370) we could not detect evidence for phospholipid binding. Rather, the protein distribution consisted of two species, one corresponding to the expected mass of *apo* STARD7(76–370) and a second, more abundant species, shifted by +60 Da. (Fig. 1c). This peak was readily eliminated by low in-source activation, suggesting non-covalent binding (Supplementary Fig. 3a). In deuterated ammonium acetate-d7 buffer (200 mM), the peak was shifted by 3 Da (Supplementary Fig. 3b), and its relative intensity varied with ammonium acetate concentration (Supplementary Fig. 3c), confirming that it corresponds to a 1:1 STARD7:acetate adduct derived from the native MS buffer. We tested alternative buffers to obtain a mass spectrum in the absence of acetate. Ammonium formate formed analogous 1:1 formate adducts with STARD7, while ammonium bicarbonate produced nonspecific adducts (Supplementary Fig. 3d, e). Lipid binding was not observed under any condition tested. We therefore performed LC-MS/MS lipidomics on purified STARD2, STARD7, and STARD10 to quantify copurified lipids prior to buffer exchange. STARD7 copurified with an order of magnitude fewer phospholipids than STARD2 and STARD10 and is predominantly in its *apo* form when purified from *E. coli* (Supplementary Fig. 4). The absence of lipids allows carboxylates to enter the empty lipid-binding pocket upon buffer exchange into native MS buffer. As acetate binding is not typically observed by native MS, the detection of 1:1 complexes with acetate could have biological relevance. However, any endogenously bound carboxylates would likely be lost during purification and thus remain undetected.

STARD10 purified almost exclusively as a 1:1 complex with phospholipids. The native mass spectrum showed >95% STARD10 bound to PE and PG (Fig. 1d and Supplementary Fig. 2a). The increased amount of lipids copurified with STARD10 compared to STARD2 suggests that STARD10 binds bacterial phospholipids more tightly.

To investigate competitive binding among different human phospholipid classes, we incubated purified STARD2, STARD7(76–370), and STARD10 with an equimolar mixture of dioleoyl PC (DOPC), dioleoyl PE (DOPE), dioleoyl PG (DOPG), dioleoyl phosphatidylinositol (DOPI), and dioleoyl phosphatidylserine (DOPS) in detergent micelles (1:5 protein:lipid ratio) (Fig. 1a). We then analyzed the bound lipids using the multistage native MS approach described above (Fig. 1b–d). STARD2 bound exclusively to DOPC, which replaced all bacterial phospholipids, underscoring its high intrinsic selectivity for PC. STARD7(76–370) bound to DOPC as well, albeit at low abundance, with most of the protein remaining in its *apo* or acetate-bound form, as further discussed in the following section. STARD10 bound not only to DOPC, but also to DOPE and DOPG in equal amounts. The bacterial phospholipids were not entirely replaced, further underscoring selective binding of PE and PG by STARD10.

In addition to lipid headgroup selectivity, we examined whether STARD2 and STARD10 preferentially copurified with bacterial phospholipids with specific acyl chain compositions by comparing the acyl chain profile of copurified phospholipids with that of total *E. coli* lipid extracts obtained from the same cell pellets (Supplementary Fig. 2b). For STARD2, we observed a strong positive correlation between the relative abundance of copurified lipids and their abundance in the bacterial membranes (Pearson’s *r* = 0.771), implying minimal selection of specific acyl chains by the transporter (Fig. 1e). In contrast, the relative abundances of lipids that copurified with STARD10 were weakly correlated with the relative abundances of total lipids from STARD10-expressing bacteria (Pearson’s *r* = 0.021). Specifically, STARD10 copurified with a relative excess of di-unsaturated lipids, notably PE and PG 36:2 and 34:2. This observation suggests intrinsic selectivity of STARD10 for phospholipids with unsaturations in both acyl chains.

### Role of a conserved arginine for lipid binding

**Fig. 2:**
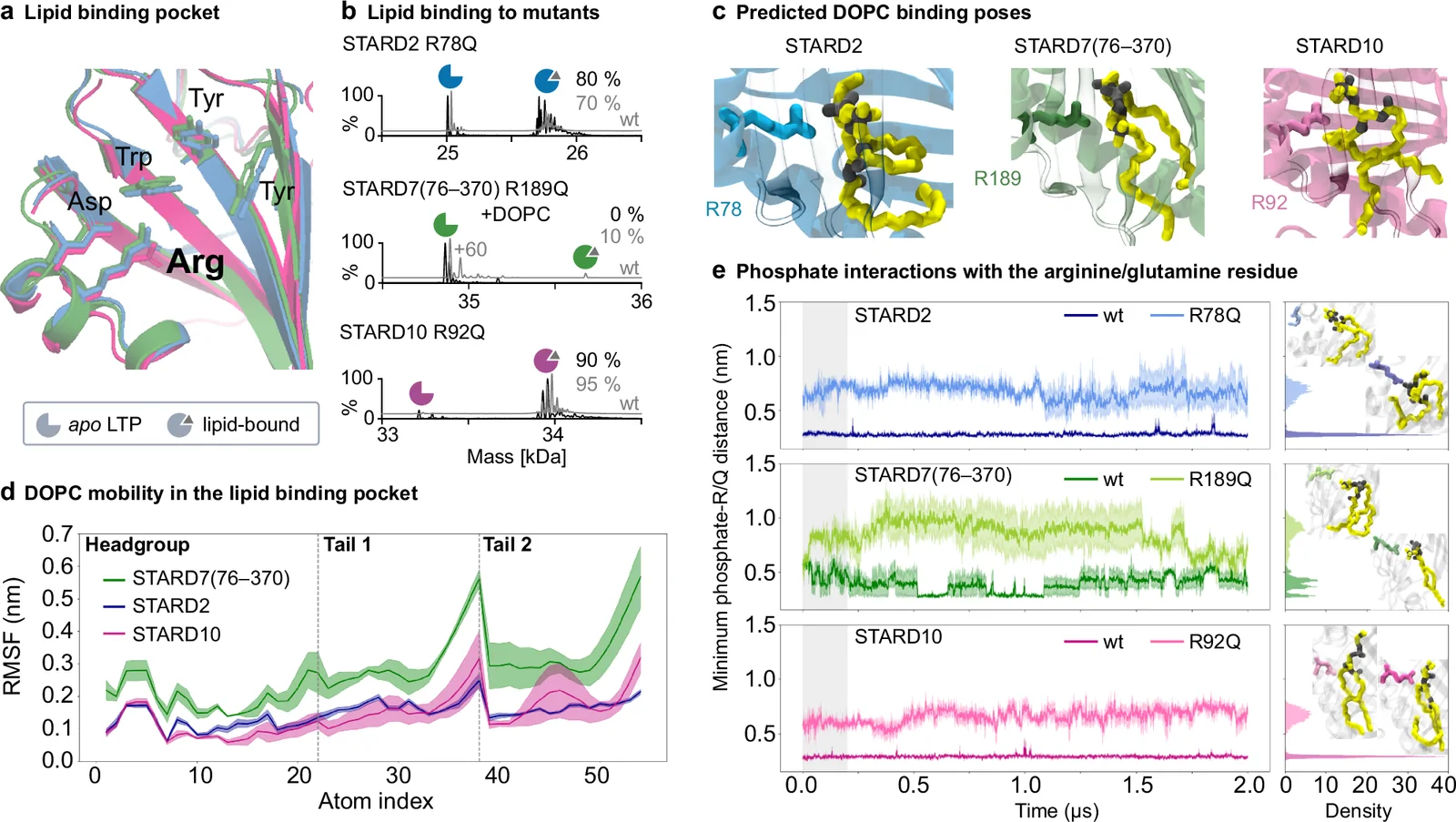
Role of a conserved arginine for phospholipid binding in STARD2 (blue), STARD7 (green), and STARD10 (purple). **a** Overlay of lipid-binding pockets, with the aromatic cage and Asp-Arg residues highlighted (AlphaFold structures). **b** Quantification of lipid binding to R → Q mutants based on deconvolved native mass spectra (black). Deconvoluted spectra of the corresponding wild-type (wt) proteins are shown for comparison (gray). The y-axis represents relative peak intensity (%), and lipid binding was quantified by integrating peak areas corresponding to *apo* and lipid-bound proteins, rounded to ±5%. STARD2 R78Q and STARD10 R92Q copurify with comparable amounts of bacterial lipids as the wt proteins upon expression in *E. coli*. Contrary to STARD7, STARD7 R189Q binds neither acetate nor DOPC after expression in *E. coli* and incubation with 100 µM DOPC in 0.5% C8E4 micelles. **c** Boltz-2 predicted structures of DOPC-bound STARD2, STARD7(76–370), and STARD10. **d** Mobility of DOPC inside the cavity as shown by the RMSF of DOPC in AA-MD. The shaded region is the standard error of the mean. **e** Minimum distance between the phosphate group of DOPC and the conserved arginine (dark traces) or the mutated glutamine (light traces) residues. Insets show the lipids inside the binding pockets at the end of the simulation (R in dark, Q in light). The shaded region around the solid line is the standard error of the mean. The first 0.2 µs of simulation, shaded in gray, were excluded in the probability density calculation and the RMSF calculation. Source data are provided as a Source Data file.

The behavior of STARD7 was unexpected, as only a limited fraction engaged in lipid binding in vitro. We further noted that acetate was only bound to the *apo* protein but not to the protein–DOPC complex after incubation with synthetic lipids, suggesting that acetate competed with PC binding to STARD7. To investigate how lipid binding in STARD7 differs from STARD2 and STARD10, we considered the arginine residue located in the lipid-binding pocket in a consensus sequence (YRKK/QWD) conserved in all three proteins (Fig. 2a)^14^. In the X-ray crystal structure of STARD2, this arginine contacts the phosphoryl group of PC ligands^13^, and in STARD7 it is crucial for phospholipid binding^14^. We expressed the three R → Q mutants STARD2 R78Q, STARD7(76–370) R189Q, and STARD10 R92Q in *E. coli* and quantified the percentage of LTP that copurified with lipids by native MS (Fig. 2b and Supplementary Fig. 5). The deconvoluted native mass spectra of STARD2 R78Q and STARD10 R92Q show that the mutation of the arginine residue does not compromise phospholipid binding, as bacterial phospholipids copurified in similar abundance as with the wild-type proteins. STARD7(76–370) R189Q did not copurify with phospholipids, as previously observed for wild-type STARD7. However, unlike the wild-type protein, STARD7(76–370) R189Q did not bind PC after incubation with excess DOPC (1:5 protein:lipid ratio) in 0.5% C8E4, whereas the wild-type protein showed clear DOPC uptake under the same conditions. Furthermore, we did not observe acetate binding in the native mass spectrum of STARD7(76–370) R189Q. We conclude that arginine 189 is a key residue for both phospholipid and acetate binding in STARD7 and propose that its electrostatic interaction with acetate diffusing into the binding pocket from the native MS buffer is central to trapping the unusual acetate adduct observed previously. Whether its ability to bind carboxylates such as acetate is relevant for the physiological function of STARD7 remains to be determined. Together, these findings suggest a different lipid binding mode in STARD7 compared to STARD2 and STARD10.

To gain further insights into the molecular mechanism behind these different binding profiles, we turned to all-atom molecular dynamics (AA-MD) simulations (Supplementary Table 2). As starting models for the simulations, we first obtained structural models for DOPC-bound STARD2, STARD7(76–370), and STARD10, using Boltz-2^40^ (Fig. 2c). For STARD2 and STARD7, the predicted binding poses are consistent with the location and orientation of dilinoleoylphosphatidylcholine (DLPC) in the crystal structure of STARD2, while for STARD10 the DOPC choline group adopts a different orientation (Supplementary Fig. 6a), likely due to the absence of the aromatic cage in STARD10. The structural models are very similar when different prediction tools and approaches (with or without guided template prediction) are used (Supplementary Fig. 6b). The AA-MD simulations indicated that DOPC is highly mobile inside the cavity of STARD7 compared to STARD2 or STARD10, as highlighted by the average root mean square fluctuation (RMSF) of the bound lipid (Fig. 2d). Further analysis of the minimum distance between the conserved arginine and the phosphate headgroup of DOPC indicates that while this interaction was stably preserved throughout the simulation for STARD2 and STARD10, this was not the case for STARD7 (Fig. 2e, dark traces). Similarly, the interaction of the DOPC headgroup with aromatic residues inside the binding cavity remained stable throughout the simulations for both STARD2 and STARD7 (Supplementary Fig. 7a, b). Mutation of the conserved arginine into a glutamine (R → Q mutation), affected the interaction between the DOPC phosphate group and the residue in all three cases, though STARD7 clearly showed the highest disruption with phosphate-glutamine distances reaching 1 nm (Fig. 2e, light traces), also resulting in disruption of the interaction between the polar headgroup of DOPC and the aromatic cage of STARD7^22^ (Supplementary Fig. 7a, b). Interestingly, while the choline group exhibits comparable binding to the aromatic cage in STARD2 and STARD7 (Supplementary Fig. 7b), upon mutation of the conserved arginine residue into glutamine, the binding of the choline group with the aromatic cage appeared more weakened in STARD7 than in STARD2 (Supplementary Fig. 7a, b). The predictions further confirmed preferential binding of acetate to the conserved Arg189 residue in STARD7, providing a rationale for the loss of carboxylate binding in the R → Q mutant (Supplementary Fig. 6c). Taken together, our data suggest that STARD7 engages in weaker interactions with PC lipids compared to STARD2 and STARD10, and that, as a result, the R → Q mutation in STARD7 has a higher impact on PC binding when compared to STARD2 and STARD10.

### Endogenous ligands reveal acyl chain selectivities

**Fig. 3:**
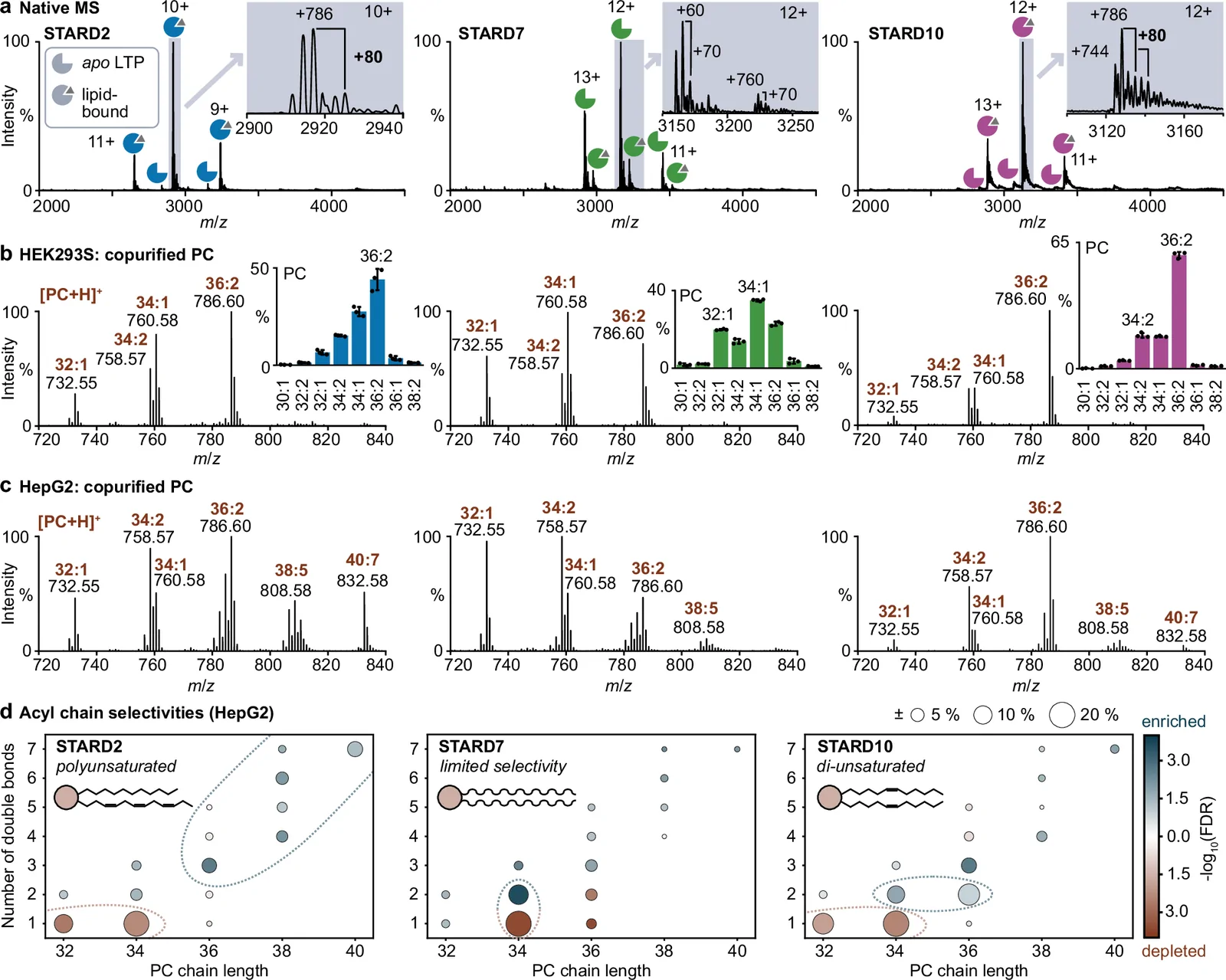
Characterization of copurified phospholipids reveals acyl chain selectivities. Endogenous lipids copurified with STARD2 (blue), STARD7 (green), and STARD10 (purple) expressed in HEK293S cells (**a**, **b**) and HepG2 cells (**c**, **d**). **a** Native mass spectra of the LTPs expressed in HEK293S cells. The spectra allow estimation of the *apo*:lipid-bound protein ratio and suggest partial phosphorylation (+80 Da) of STARD2 and STARD10. The mitochondrial localization signal of STARD7 is cleaved at two alternative sites, yielding predominantly STARD7(79–370) and a minor fraction of STARD7(78–370), which displays a mass shift of +70 Da. **b** MS^2^ spectra resulting from isolation and collisional activation of intact protein–lipid complexes obtained from HEK293S cells. In positive ion mode, protonated PC (orange) is released from all proteins, with distinct acyl chain profiles for each LTP. Relative abundances in the PC histograms were normalized to the sum of intensities of all detected PC species. Bar heights represent the mean value, and error bars indicate the standard deviation across three biological replicates. **c** MS^2^ spectra showing lipids released from STARD2, STARD7, and STARD10 after expression in HepG2 cells. **d** PC acyl chain selectivity of STARD2, STARD7, and STARD10 expressed in HepG2 cells. The relative abundance (%) of each PC species was obtained based on relative intensities in the MS^2^ spectra and subtracted from their relative abundance in HepG2 lipid extract to measure enrichment or depletion. The magnitude of this difference is encoded by circle area, while color indicates enrichment or depletion (red = depleted, blue = enriched by the protein). The color intensity represents statistical significance and is proportional to −log_10_ of the false discovery rate (FDR). Two-sided Welch’s t-tests were used to compare biological triplicate measurements between purified protein and cellular lipid extract and corrected for multiple hypothesis testing using the Benjamini–Hochberg FDR procedure. STARD2 enriches PC lipids with long, polyunsaturated acyl chains, STARD7 copurifies with PC species with limited acyl chain selectivity except an inverted ratio of PC 34:1 and 34:2, and STARD10 enriches di-unsaturated PC, especially PC 36:2. Source data are provided as a Source Data file.

In their native cellular environment, the activity and lipid cargo of LTPs are likely governed by factors beyond intrinsic lipid selectivity, including phospholipid availability and spatial organization. Thus, to identify the endogenous ligands of STARD2, STARD7, and STARD10, we next expressed the proteins with a C-terminal FLAG tag in human HEK293S cells (Supplementary Fig. 8 and Supplementary Table 1). The native mass spectrum of STARD2 showed that >95% of the protein copurified with phospholipids, suggesting stronger binding to human than to *E. coli* lipids (70%) (Fig. 3a). We further noted that the lipid binding pattern is repeated with an 80 Da mass shift, suggesting partial phosphorylation of the protein, as discussed in more detail below. We characterized copurified lipids using multistage native MS and found that they were exclusively PC lipids (Fig. 3b).

Full-length STARD7 was similarly expressed with its mitochondrial targeting sequence. The resulting native mass spectrum revealed mainly *apo* STARD7 with cleaved mitochondrial localization signal and abundant 1:1 binding of acetate (+60 Da) as for the *E. coli* construct. In contrast, only a minor fraction (ca. 15%) of lipid-bound STARD7 was observed. Accordingly, STARD7 is mainly obtained in its *apo* form even when expressed in human cells in the presence of its native ligands. Using native top-down MS, we localized the main cleavage site between Ala78|Leu79 and found a minor isoform (ca. 25%) cleaved between Ala77|Ala78 (Supplementary Fig. 9). Alternative cleavage of the STARD7 mitochondrial localization signal has been reported previously but at an alternate site that we did not observe (Met76|Ala77)^18^. Through isolation and activation of lipid-bound STARD7 complexes, we found that STARD7 copurified exclusively with PC lipids.

STARD10 expressed in HEK293S cells yielded a native mass spectrum that contained >95% STARD10 bound to endogenous ligands. The pattern of peaks was complex, and repeated twice at intervals of 80 Da, suggesting phosphorylation of the protein (discussed below). Multistage native MS indicated that STARD10 copurified with PC, PE, plasmenyl PE (PE-P), and PG. PE (-P) and PG were detected upon complex activation in negative ion mode, in accordance with their ionization preferences (Supplementary Fig. 10). STARD10 thus binds PC, PE, and PG headgroups, consistent with our in vitro binding experiment and a recent LC-MS-based characterization of lipids associated with STARD10^16^.

We noted that STARD2, STARD7, and STARD10 copurified with PC species with distinct acyl chain preferences, as illustrated by the PC histograms reconstructed from the MS^2^ peak intensities (Fig. 3b and Supplementary Table 3). Correlation of copurified PC species with total cellular lipid extracts indicated that STARD7 most closely mirrored membrane lipid availability (Pearson’s *r* = 0.840), whereas STARD10 showed the lowest degree of similarity (Pearson’s *r* = 0.521) of the three proteins (Supplementary Fig. 11). However, the lipid repertoire of HEK293S cells is relatively restricted and largely dominated by monounsaturated PC species^41^, which may limit the generalizability of our results. To investigate acyl chain selectivity in a context of broader lipid diversity, we characterized StARD cargo in HepG2 (hepatocellular carcinoma) cells, which possess a richer lipid composition, notably including polyunsaturated PCs. The three StARD proteins expressed in HepG2 cells (Supplementary Fig. 12) yielded comparable native mass spectra to their HEK293S counterparts (Supplementary Fig. 13a), with some changes in copurified lipids (Fig. 3c and Supplementary Table 4).

The acyl chain profiles of PC copurified with STARD2, STARD7, and STARD10 differed both from one another and from total lipid extracts obtained from the cell pellets (Supplementary Fig. 13b). Several lipid species were enriched, whereas others were depleted relative to their abundance in cellular membranes. To quantify enrichment or depletion, we calculated the relative abundance of each PC species normalized to the total PC signal intensity in the MS^2^ spectra and subtracted the corresponding relative abundance measured in the cellular lipid extract. The resulting differences are represented in blue (protein-associated enrichment) and red (depletion), with circle area reflecting the absolute deviation from the cellular lipid composition (Fig. 3d). We found that STARD2 enriched long, polyunsaturated PC species (C36–40) and showed marked depletion of monounsaturated PCs. In contrast, STARD7 exhibited little apparent selectivity, with the exception of an inverted ratio of PC 34:1 vs. PC 34:2. The PC profile copurified with STARD10 in HepG2 cells differed only minimally from that obtained in HEK293S cells, underscoring the strong intrinsic selectivity of STARD10 for di-unsaturated PC 34:2 and PC 36:2 and its concomitant depletion of monounsaturated PCs. To investigate the binding of lipids with different acyl chain saturations in silico, we performed AA-MD simulations for STARD2 and STARD7 bound to stearoyl-arachidonoyl-phosphatidylcholine (SAPC) and palmitoyl-oleoyl-phosphatidylcholine (POPC), respectively. Consistent with previous observations for DOPC, SAPC in STARD2 exhibited lower mobility within the cavity and maintained more persistent interactions with the conserved arginine compared to POPC in STARD7 (Supplementary Fig. 7c–e).

### Acyl chain selectivities influence lipid metabolism

**Fig. 4:**
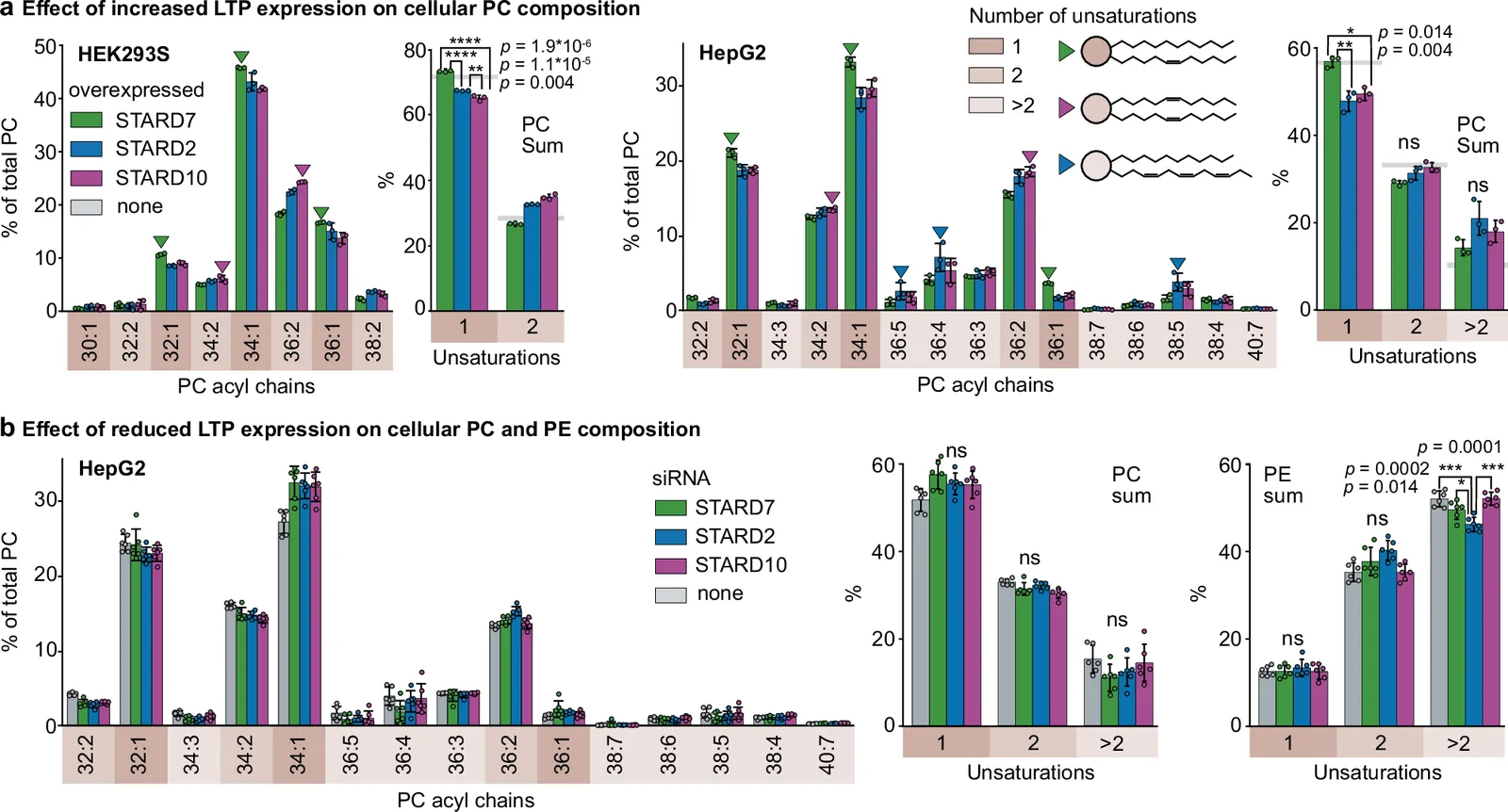
Impact of LTP expression levels on lipid metabolism. **a** Relative quantification of PC species in lipid extracts obtained from HEK293S and HepG2 cells overexpressing STARD2, STARD7, or STARD10 for 48 h compared to wild-type cells (gray bars). Overexpression of STARD2 or STARD10 consistently decreased the relative abundance of monounsaturated PC. Colored triangles point to the condition with the highest abundance for individual PC species (monounsaturated PC in STARD7-, di-unsaturated PC in STARD10-, and polyunsaturated lipids in STARD2-overexpressing cells). **b** Relative quantification of PC and PE species in HepG2 cells after siRNA-mediated knockdown of STARD2, STARD7, or STARD10. PC abundances were not significantly altered across conditions, whereas polyunsaturated PE species were significantly depleted following STARD2 knockdown (for detailed PE analysis see Supplementary Fig. 17). Bars represent the mean of three (**a**) or six (**b**) biological replicates, and error bars represent the standard deviation. To assess significance in (**a**, **b**), data were centered log-ratio (CLR) transformed to account for compositional constraints, and statistical significance was determined using one-way ANOVA, with pairwise comparisons by Tukey’s HSD. Statistical significance is indicated based on adjusted *p*-values: *p < 0.05, **p < 0.01, ***p < 0.001, ****p < 0.0001; ns = not significant. Source data are provided as a Source Data file.

Given that the StARD phospholipid transporters copurify with PC species with distinct acyl chains, we hypothesized that they selectively extract these lipids from cellular membranes, and that the expression levels of these LTPs may thus influence cellular lipid equilibria. To investigate whether overexpression of STARD2, STARD7, or STARD10 affected lipid metabolism, we analyzed whole-cell lipid extracts from HEK293S and HepG2 cells after 48 h of tetracycline-induced protein overexpression. Quantification of individual PC species revealed that overexpression of these LTPs modulated the overall cellular PC acyl chain composition (Fig. 4a). Overexpression of STARD2 or STARD10 consistently increased the abundance of unsaturated PC species in both cell lines. In contrast, STARD7 overexpression did not alter PC unsaturation levels compared to wild-type cells. This finding is intriguing, as the preferential binding of unsaturated lipids by STARD2 and STARD10 noted earlier may explain the observed increase in these species, underscoring a potential link between LTP binding specificity, expression levels, and cellular lipid composition.

We further investigated if overexpression of STARD2, STARD7, or STARD10 could also alter the acyl chain profiles of other phospholipids that are synthesized at the ER and share the same acyl-CoA pool as PC, most notably PE, PS, and PI (Supplementary Figs. 14 and S15). In the lipid extracts of HEK293S and HepG2 cells overexpressing the individual LTPs, we observed the most significant changes in the acyl chain composition of PI. Consistent with the observations made for PC, di- and polyunsaturated PI populations were significantly enhanced in cells overexpressing STARD2 or STARD10. Similar remodeling of PE was also observed, though these changes were not statistically significant in HEK293S cells. PS acyl chains were less affected, and PE-P and sphingomyelin showed no change. These observations are consistent with known lipid synthesis routes^42^, wherein PE-P pools undergo less acyl chain remodeling than PE, and the sphingomyelin side chains are built from a different biosynthetic route than the glycerophospholipids. Taken together, we observe consistent trends in acyl chain remodeling depending on the expression levels of STARD2, STARD7, and STARD10 only for phospholipids that are closely linked to PC synthesis.

To assess the effect of the reverse condition, i.e., reduced LTP expression levels, we also performed lipidomic profiling following siRNA-mediated knockdown of each protein in HepG2 cells. After 72 h, we observed an approx. 3-fold reduction in LTP abundances in HepG2 cells (Supplementary Fig. 16 and Supplementary Table 5). The cellular lipidomes showed only modest changes in PC species, with the most pronounced differences observed between the control and the three knockdown conditions for PC 34:1 (Fig. 4b). In contrast, PE species were significantly affected. Most notably, STARD2 depletion led to a reduction in polyunsaturated PE species compared to the other three conditions (Fig. 4b and Supplementary Fig. 17). This finding is consistent with our previous observation that overexpression of STARD2 increases the levels of polyunsaturated phospholipids. Polyunsaturated PCs were also slightly reduced in STARD2 knockdown cells, but this difference was not statistically significant, possibly due to their low abundance compared to PE, where polyunsaturated species predominate. Overall, siRNA-mediated protein knockdown produced more subtle changes in the lipidome than protein overexpression; however, together these data strongly indicate interactions between LTP expression levels and overall lipidomic profiles.

Given the relationship between LTP expression and cellular lipid composition, we hypothesized that LTP expression levels may correlate with lipid metabolism across tissues. We therefore examined whether LTP expression varies in accordance with the predominant lipid species produced in different organs. Based on transcriptomics data from the EMBL-EBI Expression Atlas^43^, *STARD7* RNA expression levels are largely invariant between human tissues, whereas *STARD2* and *STARD10* transcripts are selectively enriched in fewer regions, most notably in liver (Supplementary Fig. 18a). In line with the high expression levels of STARD2 and STARD10 in liver, a high percentage of their preferred lipid ligands, PC 34:2 and 36:2 and polyunsaturated PCs, was reported in human liver biopsies (Supplementary Fig. 18b)^44,45^. On the contrary, two independent studies on human heart sections (where STARD10 is expressed 10-fold less) did not detect any of the di-unsaturated PC lipids selectively bound by STARD10^46,47^. It should be noted that these data are limited by the fact that protein expression levels are inferred from transcriptomic analyses rather than direct protein measurements, and that comparable high-quality lipidomics datasets across human tissues are currently lacking for broader analysis. Nonetheless, the currently available data lead us to hypothesize that the acyl chain selective LTPs STARD2 and STARD10 may exhibit higher expression levels in tissues with higher levels of PC unsaturation.

### Localization of phosphorylation sites in STARD2 and STARD10

**Fig. 5:**
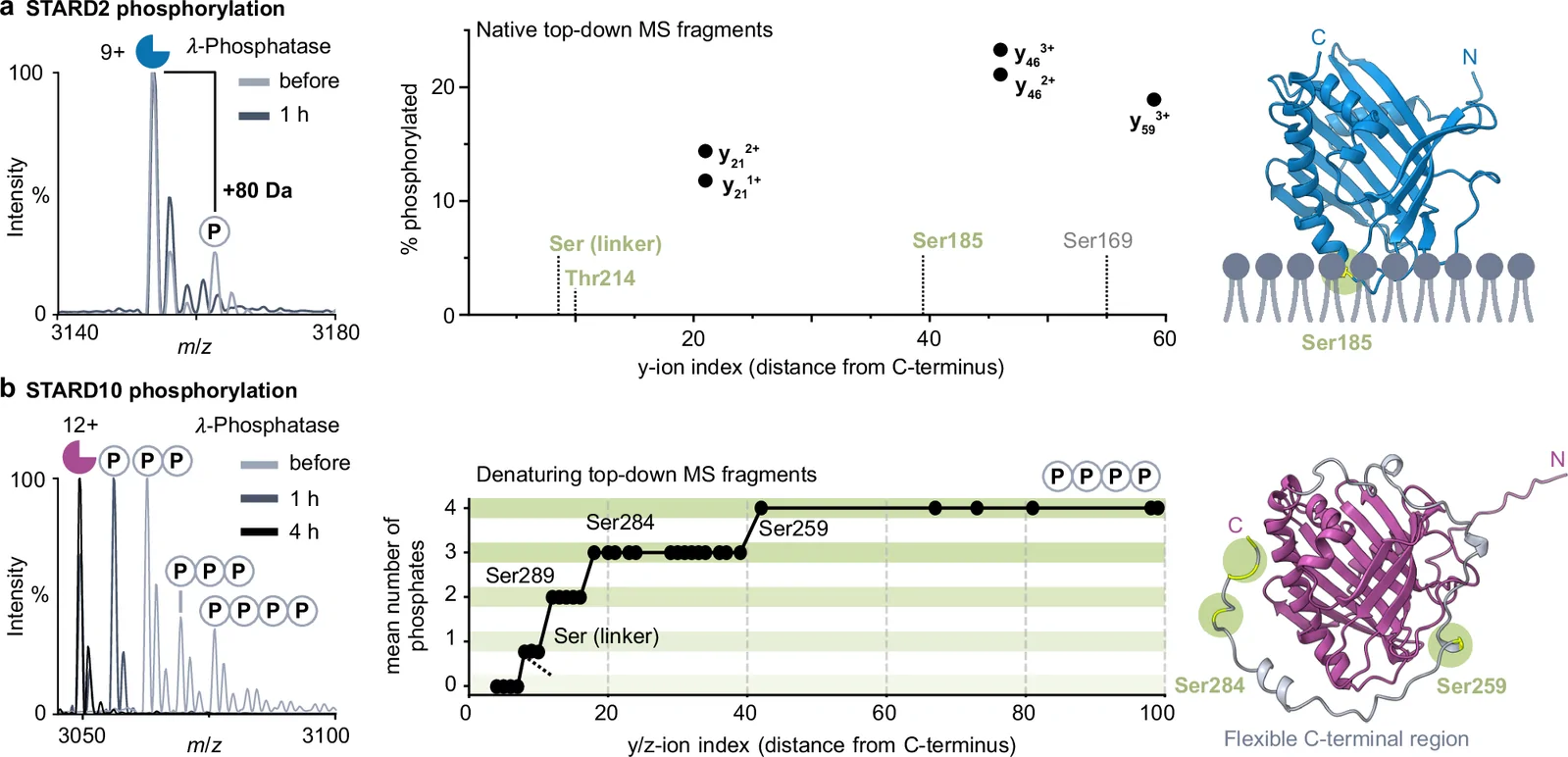
Phosphorylation of STARD2 (blue) and STARD10 (purple) in membrane-interacting regions. **a** Native mass spectra of *apo* STARD2 shown in the region of the 9+ charge state before and after treatment with λ-phosphatase, recorded with in-source activation (100 V) to remove bound lipids. Quantification of phosphorylated C-terminal y-ions generated by native top-down MS of STARD2 localizes phosphates to the C-terminus and Ser185. Structure of STARD2 interacting with a phospholipid membrane based on ref. ^22^, with Ser185 highlighted in green. **b** Native mass spectra of *apo* STARD10 recorded with in-source activation (140 V) before and after treatment with λ-phosphatase for 1 h and 4 h demonstrate that STARD10 is phosphorylated at up to four sites. Phosphates were mapped to two constitutively occupied sites (Ser259 and Ser284) and up to two partially occupied sites in the C-terminus, based on quantification of phosphorylation on C-terminal y- and z-ions generated by denaturing top-down MS. Structure of STARD10 with the flexible C-terminal region and detected phosphorylation sites highlighted. Proteins were expressed in HEK293S cells, and structures were generated using Alphafold3^76^. Source data are provided as a Source Data file.

For cytosolic LTPs, a crucial question is how they are targeted to specific membranes and how their activity is regulated. Previous studies have suggested that phosphorylation might play a key role in the regulation of STARD2 and STARD10^23,26^. To investigate such regulation, we set out to identify, quantify, and localize phosphorylation and other PTMs. The measured mass of *apo* STARD2 expressed in HEK293S and HepG2 cells was 42 Da higher than the expected mass, which we assigned to N-terminal acetylation. In addition, we observed a peak shifted by 80 Da relative to the *apo* protein, and the lipid-bound peaks were likewise reproduced with the same 80 Da shift (Fig. 3a). This pattern is consistent with partial phosphorylation of the protein (approximately 20%), as estimated from the relative peak intensities observed in the native mass spectrum. The uniform 80 Da shift across the *apo* protein and all lipid-bound states indicates that phosphorylation is not associated with any specific lipid-bound species. To accurately quantify the phosphorylated species, subsequent native mass spectra were acquired with in-source activation to dissociate all bound lipids, reducing the spectrum to two predominant peaks corresponding to *apo* STARD2 and its phosphorylated form. Phosphorylation of STARD2 was confirmed by treatment with λ-phosphatase, which eliminated the +80 Da peak (Fig. 5a).

To localize the phosphorylation site(s), we fragmented the protein in a native top-down experiment and used precisION^48^ for fragment assignment and modification discovery (Supplementary Fig. 19a). We found that all *b*-type sequence ions containing the N-terminus were shifted by 42.01 Da, confirming that STARD2 was entirely N-acetylated. Following enzymatic removal of the C-terminal FLAG tag, we were able to observe phosphorylated *y*-type ions stemming from the protein’s C-terminus (Supplementary Fig. 19b). We identified two phosphorylation sites each with ca. 10% occupancy: (i) near the C-terminus and (ii) at Ser185 (Fig. 5a and Supplementary Fig. 19c). A complementary proteomics analysis detected low-abundance phosphorylated peptides (2–3% rel. to unmodified) containing the C-terminus and Ser185 (Supplementary Fig. 20). Intriguingly, Ser185 is located directly at the protein–membrane interface, close to the lipid entry site of STARD2, which is a prominent position to mediate membrane association and/or lipid entry (Fig. 5a)^22^. To our knowledge, this is the first experimental evidence for STARD2 phosphorylation at this site.

For STARD7, we found no evidence of phosphorylation using either intact mass measurements or proteomics approaches. In contrast, native MS of STARD10 revealed an intact mass that exceeded the theoretical value by 202 Da. Using native top-down MS, we confirmed complete N-acetylation of STARD10 (Supplementary Fig. 21). Taking this PTM into account, the remaining mass shift of 160 Da could correspond to two phosphorylation sites. Enzymatic treatment of the protein with λ-phosphatase confirmed our hypothesis: we obtained two new peaks corresponding to monophosphorylated and *apo* STARD10 (Fig. 5b). STARD10 overexpressed in HEK293S or HepG2 cells is thus constitutively phosphorylated at two sites and has two additional phosphorylation sites that are partially occupied.

Proteomics analysis of STARD10 yielded two phosphorylated peptides, H229-R272 and M276-H322, which were 95% and 90% phosphorylated, respectively (Supplementary Fig. 22). Because native top-down MS did not yield fragments in the region of interest, we denatured the protein to obtain precursor ions with higher charge states that undergo more extensive fragmentation. We then activated the 28+ charge state using electron transfer dissociation with supplemental HCD (EThcD). For the doubly phosphorylated base peak, we clearly identified two phosphorylation sites, both 100% occupied: Ser259 and Ser284 (Supplementary Fig. 23a). Ser259 was previously identified as a constitutively phosphorylated site of STARD10 in breast epithelial cells^24,25^, and phosphorylation at Ser284 has been identified as an activity regulator in HEK293T cells^26^. Our results reveal that both sites are entirely phosphorylated under overexpression conditions. By fragmenting the proteoforms containing three and four phosphates, we confidently mapped the two partially occupied phosphorylation sites to the serine in the linker (GSG) and the C-terminus (TSLT) (Fig. 5b and Supplementary Fig. 23b,c). Accordingly, all phosphorylation sites detected here are in the flexible C-terminal region of STARD10 and can potentially serve as a regulatory mechanism to influence membrane association, lipid entry, or yet uncharacterized protein–protein interactions (Fig. 5b).

### Regulation of lipid transfer activity

**Fig. 6:**
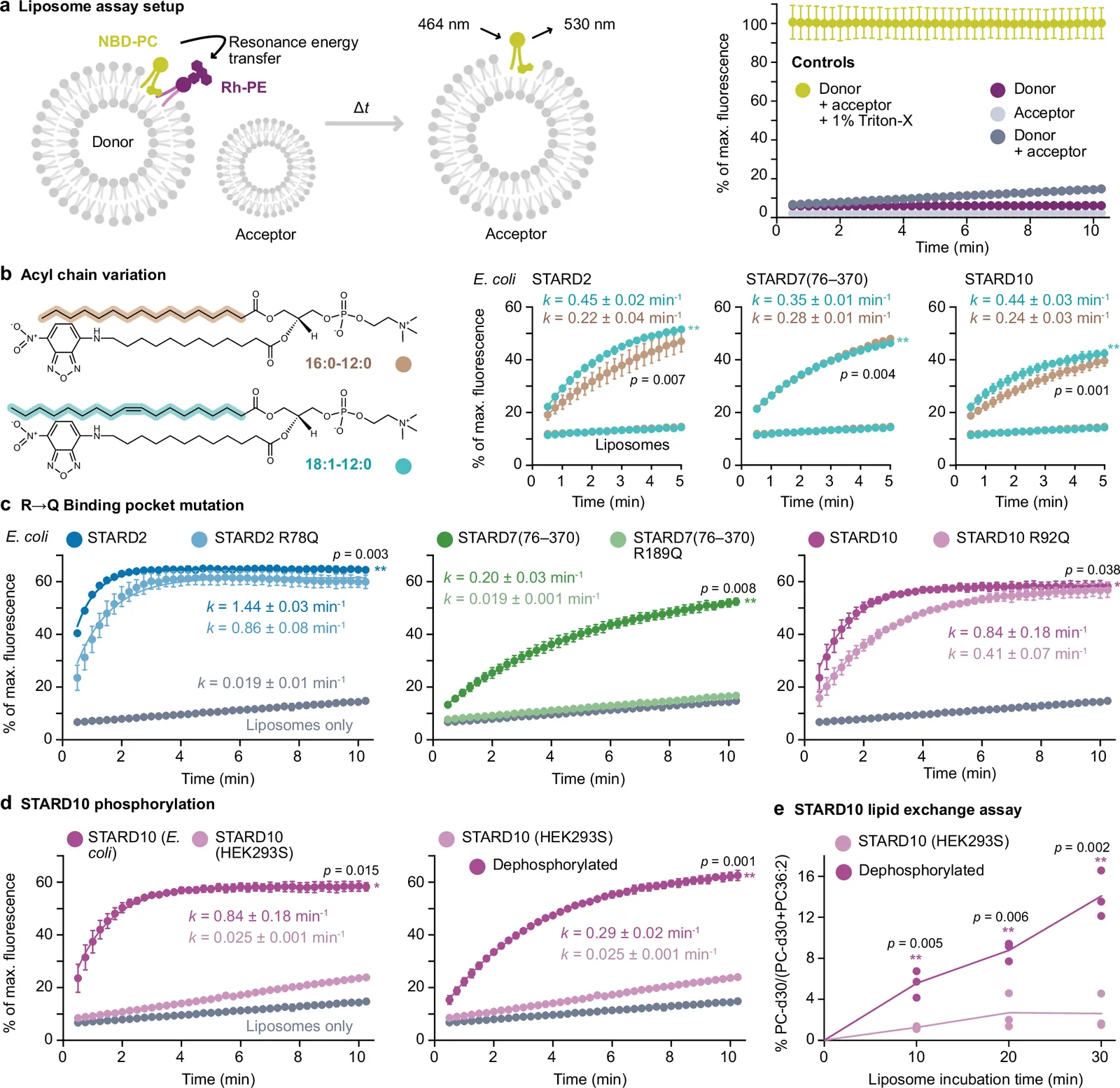
Liposome assays correlating acyl chain selectivities, binding pocket mutation, and phosphorylation with lipid transfer activity in vitro. **a** The fluorescence of NBD-PC (1 mol%) is quenched by Rh-PE (5 mol%) in donor liposomes and increases as NBD-PC is transported to acceptor liposomes. Control measurements were performed for individual donor (37.5 nmol/mL) and acceptor (75 nmol/mL) liposomes, mixed donor and acceptor liposomes, and mixed liposomes treated with Triton-X (1%) to determine the maximum fluorescence. **b** Lipid transfer assays using donor liposomes containing NBD-PC 16:0-12:0 or NBD-PC 18:1-12:0, mediated by STARD2, STARD7(76–370), or STARD10 expressed in *E. coli*. STARD2 and STAR10 transport unsaturated NBD-PC twofold faster than saturated NBD-PC, whereas the difference is only ca. 20% in STARD7-mediated lipid transfer. To obtain comparable initial slopes, STARD2 and STARD10 were added at reduced concentration (0.05 nmol/mL and 0.10 nmol/mL, respectively). **c** Arginine-to-glutamine mutation abolishes PC transfer for STARD7(76–370) but only leads to a twofold reduction in transfer speed for STARD2 and STARD10. **d** Comparison between the lipid transfer activities of STARD10 expressed in *E. coli* (not phosphorylated) and in HEK293S cells (phosphorylated) shows that phosphorylation of STARD10 significantly reduces its lipid transfer activity. Lipid transfer is partially recovered following enzymatic dephosphorylation of STARD10 in vitro. Data points in (**a**–**d**) show mean values with error bars representing the standard deviation of three technical replicates. First-order rate constants (*k*) were calculated for individual replicates and are reported as mean ± standard deviation. Pairwise statistical significance between the two conditions in (**b**–**d**) was assessed using two-sided Welch’s t-test. Statistical significance is indicated based on *p*-values: *p < 0.05, **p < 0.01, ***p < 0.001. Donor liposomes in (**b**–**d**) contained NBD-PC 16:0-12:0, and the protein concentration was 0.25 nmol/mL, unless otherwise stated. **e** Incubation of STARD10 with liposomes containing deuterated PC 16:0-d30/18:1 (PC-d30), before and after dephosphorylation, shows that phosphorylation impedes lipid uptake from liposomes, whereas dephosphorylated STARD10 exhibits clear time-dependent uptake of deuterated PC. Lipid uptake was quantified by measuring the MS^2^ intensity of protonated PC-d30 (*m*/*z* 790.78) relative to that of the most abundant endogenous PC 36:2 (*m*/*z* 786.60). Data points represent three technical replicates, and the line graph represents the mean value. Statistical significance was assessed using two-sided Welch’s t-test. Statistical significance is indicated based on *p*-values: *p < 0.05, **p < 0.01, ***p < 0.001. Source data are provided as a Source Data file.

Lastly, we sought to correlate our combined findings on acyl chain selectivity, binding pocket mutations, and phosphorylation with the lipid transfer activity of STARD2, STARD7, and STARD10 in vitro. We used a liposome assay based on resonance transfer between nitrobenzoxadiazole-PC (NBD-PC) and rhodamine-PE (Rh-PE) to measure the transfer of fluorescent NBD-PC between two types of liposomes (Fig. 6a)^11^. When donor and acceptor liposomes were mixed in the absence of LTPs, we observed a slow, linear increase in fluorescence, confirming that NBD-PC diffused from donor to acceptor liposomes. The maximum possible fluorescence that we used as a reference in the following measurements was obtained by adding Triton-X to the mixed liposomes.

In the first series of measurements, we hypothesized that the different acyl chain preferences of STARD2, STARD7, and STARD10 would lead to distinct lipid transfer kinetics of NBD-PC probes with different acyl chains. We compared the initial lipid transfer rates between NBD-PC 16:0–12:0 and 18:1–12:0 using catalytic concentrations of recombinantly expressed proteins from *E. coli* (Fig. 6b). In the presence of STARD2 or STARD10, the first-order rate constants (*k*) were approximately twofold higher for the unsaturated than the saturated NBD-PC probe. For STARD7(76–370), the difference in rate constants between the two lipid probes was only ~20%, indicating a significantly smaller effect. The results support our hypothesis that acyl chain preferences translate into differential lipid transfer kinetics.

Next, we investigated the lipid transfer abilities of the R → Q mutants (Fig. 6c). The wild-type proteins expressed in *E. coli* significantly increased lipid transfer relative to passive diffusion of NBD-PC, though STARD7(76–370) expressed in *E. coli* was less active than the version purified from HEK293S cells (Supplementary Fig. 24a). The STARD2 R78Q and STARD10 R92Q mutants retained efficient PC transfer, with rate constants approximately twofold lower than those of the wild type. STARD7(76–370) R189Q, on the other hand, showed no lipid transfer activity. The measured rate constant was indistinguishable from that obtained upon mixing donor and acceptor liposomes in the absence of LTPs. This finding consolidates our MS results, which showed that lipid binding is abolished by point mutation of the arginine residue in the binding pocket of STARD7 but not STARD2 or STARD10.

To measure the impact of phosphorylation of STARD2 and STARD10 on lipid transfer, we compared the transport activity of non-phosphorylated STARD2 and STARD10 recombinantly expressed in *E. coli* with the transport activity of the proteins expressed in HEK293S cells. For STARD2, we found no significant difference between the partially phosphorylated and non-phosphorylated protein (Supplementary Fig. 24b). Either the phosphorylation did not affect the lipid transfer ability, or, more likely, the ratio of phosphorylated protein was too low to make the effect clearly visible. By contrast, phosphorylated STARD10 had a significantly reduced lipid transfer ability compared to the non-phosphorylated protein expressed in *E. coli* (Fig. 6d). To test if we could recover its lipid transfer ability by removing the phosphates, we treated STARD10 from HEK293S cells with λ-phosphatase and confirmed complete dephosphorylation by native MS. The phosphatase-treated STARD10 regained its lipid transfer activity, confirming that phosphorylation of STARD10 abolishes phospholipid transfer. This suggests that phosphorylation might indeed serve as a regulatory mechanism for lipid transfer.

To relate phosphorylation-controlled lipid transfer activity to native MS measurements, we examined whether phosphorylation of STARD10 affects lipid exchange between its lipid-binding pocket and liposomes. We chose a simplified liposome system containing deuterated PC 16:0-d31/18:1, which is readily distinguishable by mass from endogenous PC species. STARD10 expressed in HEK293S cells was incubated with these liposomes either in its native, phosphorylated state or following λ-phosphatase treatment. Native MS analysis revealed that phosphorylated STARD10 exhibited minimal exchange of endogenous PC for deuterated PC, whereas dephosphorylated STARD10 showed pronounced, time-dependent uptake of deuterated PC from liposomes (Fig. 6e and Supplementary Fig. 25). These findings suggest that phosphorylation of the flexible STARD10 C-terminus disrupts lipid transfer between the liposomes and the STARD10 lipid-binding pocket. However, this experiment does not distinguish whether the reduced lipid transfer activity of phosphorylated STARD10 arises from impaired membrane association or from a specific hindrance of lipid uptake into, or release from, the binding pocket. More broadly, these results demonstrate that native MS-based lipid binding measurements can be directly correlated with functional lipid transfer activity.

## Discussion

We describe a native MS-based approach to identify endogenous ligands of LTPs with acyl chain resolution, which we used to characterize the endogenous ligands of the human PC transfer proteins STARD2, STARD7, and STARD10. By isolating intact protein–lipid complexes from two human cell lines and identifying bound lipids using multistage native MS, we uncovered differential acyl chain selectivities of these three LTPs. We further determined PTMs and protein isoforms and related our findings to lipid transfer activity in vitro. Compared to classical approaches based on affinity purification and identification of bound lipids by separate LC-MS/MS analysis, our method offers two major advantages. First, native MS enables quantitative characterization of intact protein–lipid complexes, including protein:lipid binding stoichiometry and the relative abundance of *apo* versus lipid-bound protein. These data provide biophysical insights that manifest in functional differences. For example, the lower degree of lipid binding to STARD7 observed by native MS correlates with its reduced rate of lipid transfer in liposome assays. Second, isolation of intact protein–lipid complexes in the gas phase prior to controlled lipid release ensures that detected lipids were initially bound to the protein as specific complexes, whereas contaminant lipids are effectively excluded from the analysis. Our approach thus eliminates the need for additional lipid extraction or chromatographic separation steps while enabling deep structural analysis of lipids that selectively bind to LTPs, including their acyl chain composition.

While the acyl chain selectivity of STARD2 has been studied extensively^20,30,31,36^, it has not previously been compared to that of other major PC transporters including STARD7 and STARD10. Here we report clear differences in their acyl chain preferences, with STARD7 copurifying with a range of different PC lipids according to their natural abundance. By contrast, STARD2 shows a clear preference for long, polyunsaturated chains according to our and previous reports^20,30,31,36^, and STARD10 reproducibly copurified with di-unsaturated phospholipids in different expression systems. Previously, STARD10 had been thought to select primarily monounsaturated lipids^15^. Our results show that those lipids are actually depleted in favor of lipids with one unsaturation in each chain. Taken together, STARD2, STARD7, and STARD10 have evolved distinct acyl chain preferences, suggesting functional specialization within cells.

**Fig. 7:**
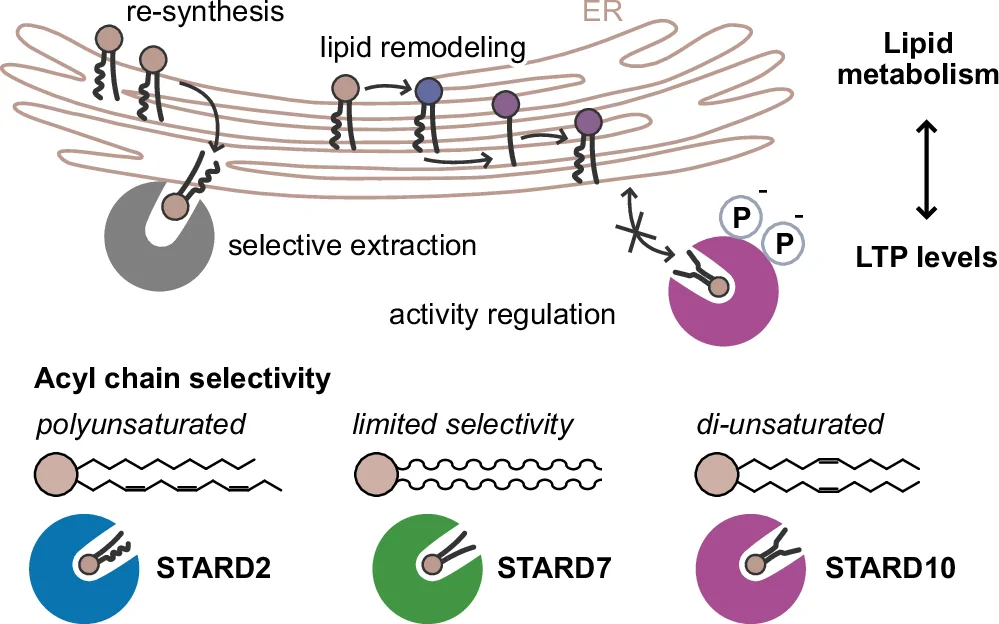
Proposed mechanism for the interplay between LTP-mediated lipid transfer and cellular lipid metabolism. Acyl chain-selective extraction of PC (beige headgroup) from the ER could lead to replenishment of the depleted acyl chain pool, causing an overall increase in the abundance of the extracted PC. The acyl chain distributions of other phospholipid classes (illustrated by changing headgroup colors) are also affected via acyl chain remodeling. Phosphorylation of STARD10 could serve as a regulatory mechanism to inhibit lipid exchange between membranes and the lipid-binding pocket, effectively reducing the lipid transfer activity of STARD10.

As LTPs mediate the majority of lipid transport in eukaryotic cells, the acyl chain-dependent differences in lipid transport kinetics must ultimately come down to preferences of LTPs for extracting and transporting lipids with certain chain length and saturation. For STARD2 and STARD10, which both prefer unsaturated lipids, this is likely related to the observation that intracellular transport of PC lipids occurs faster as the degree of unsaturation increases^37^. A possible explanation for the modulation of lipid abundances by LTP expression levels is that extraction of specific lipids by an LTP could stimulate further synthesis of the extracted lipid in the ER. This led us to hypothesize that the acyl chain-selective transport of lipids may exert a feedback control on lipid synthesis such that lipid levels are influenced by the expression levels of LTPs with different acyl chain selectivities (Fig. 7). We observed stronger effects of protein levels on lipid metabolism under overexpression conditions than for siRNA-mediated knockdown. Because the silencing is gradual and the metabolic response is delayed, it is challenging to disentangle the individual processes involved. Nevertheless, the observed alterations in polyunsaturated PE upon STARD2 knockdown suggest that LTP expression levels impact cellular lipid metabolism.

The differential acyl chain selectivities of major cytosolic phospholipid transporters could have wider implications for lipid transport in different cell types. According to the EMBL-EBI Expression Atlas (http://www.ebi.ac.uk/gxa)^43^, STARD7 is expressed at similar levels throughout human tissues, which could reflect its role as a non-selective PC transporter that is notably required for normal mitochondrial function^33,49^. On the other hand, the expression levels of STARD2 and STARD10 are highly tissue-dependent. Their acyl chain selectivities and distinct expression pattern could be linked by different lipids dominating different tissues. More generally, this points to a fine interplay between expression levels of LTPs and metabolism-dependent lipid distribution in specific tissues and cells.

We further addressed how lipid transfer activity of STARD2 and STARD10 may be regulated by reversible phosphorylation in cells. We provide direct evidence that STARD10 is phosphorylated at up to four sites and that phosphorylation abolishes its lipid transfer activity in vitro. For STARD2, we did not detect phosphorylation of the previously reported putative protein kinase C-dependent phosphorylation site Ser110^23^ but anticipate a regulatory role for the newly reported phosphorylation site at Ser185, which is located next to the lipid entry site. Phosphates introduce negative charges that may disrupt protein–membrane association and lipid exchange between the membrane and the lipid-binding pocket of LTPs. We showed by native MS that lipid transfer between liposomes and the lipid-binding pocket of STARD10 is strongly impaired by phosphorylation. However, these experiments did not reveal which step of the exchange process, i.e., protein–liposome association, lipid uptake, or lipid release, is specifically affected. Notably, STARD10 purified from human cells was entirely phosphorylated while being almost fully saturated with bound phospholipids, suggesting that phosphorylated STARD10 may still efficiently take up lipids, whereas lipid release could be impaired. Distinguishing between defects in lipid uptake versus lipid release by native MS would require completely delipidated STARD10, enabling direct measurement of lipid uptake without previous lipid release like in our lipid exchange assay. However, we were unable to delipidate STARD10, as stringent detergent washing with 2% octyl glucoside did not measurably reduce the fraction of lipid-bound protein. Together, our results confirm that phosphorylation impairs STARD10 lipid transfer activity, although the underlying mechanism and the step affected remain unresolved.

Overall, our work establishes that the lipid binding preferences and structural modifications of LTPs are closely linked to their functional activity, supporting lipid binding as a meaningful indicator of transport capacity. In this context, native mass spectrometry emerges as a powerful approach to resolve lipid binding preferences and PTMs at molecular resolution. Looking ahead, an important next step will be to extend these insights beyond in vitro systems by directly relating lipid selectivity to differential lipid transport in living cells. This could be achieved using bifunctional PC species with defined acyl chains to monitor their intracellular transport as a function of LTP expression levels. Such experiments will ultimately provide a comprehensive view of how LTP selectivity, expression levels and regulation shape lipid transport and metabolism.

## Methods

### Lipid standards

PC 18:1(9Z)/18:1(9Z) (DOPC), PE 18:1(9Z)/18:1(9Z) (DOPE), PG 18:1(9Z)/18:1(9Z) (DOPG), PS 18:1(9Z)/18:1(9Z) (DOPS), and PI 18:1(9Z)/18:1(9Z) (DOPI) were purchased from Avanti Research, dissolved in chloroform, and dried overnight in a centrifugal evaporator. The lipid films were resuspended to a final concentration of 5 mM by sonication in 200 mM ammonium acetate containing 2× critical micelle concentration (CMC) C8E4. NBD-PC 16:0-12:0, NBD-PC 18:1-12:0, and rhodamine-PE 16:0-16:0 were purchased from Avanti Research and dissolved in chloroform at a final concentration of 1 nmol/µL.

### Expression of STARD2, STARD7, and STARD10 in *E. coli*

Codon-optimized double-stranded DNA fragments (gBlocks) encoding STARD2, STARD2 R78Q, STARD7 (76–370), STARD7 (76–370) R189Q, STARD10, and STARD10 R92Q were purchased from Integrated DNA Technologies (sequences provided in Supplementary Table 6) and inserted into a modified pET28b vector using Gibson assembly, resulting in a construct encoding the respective protein with an N-terminal 6×His tag, maltose binding protein (MBP), and TEV protease cleavage site. The plasmids were transformed into *E. coli* C43(DE3) cells (New England Biolabs). Overnight cultures were grown with shaking in Luria-Broth under kanamycin selection (50 µg/mL) at 37 °C. Ten milliliters of the overnight cultures was used to inoculate 1 L of expression culture. The cultures were grown at 37 °C to OD_600_ = 0.5 and induced by the addition of IPTG to a final concentration of 0.5 mM. Induced cultures were shaken at 30 °C for 4 h. Cell pellets were harvested by centrifugation at 5000 × *g* for 15 min.

The cells were resuspended in 20 mL lysis buffer (50 mM Tris pH 8.0, 300 mM NaCl, 2.5 mM beta-mercaptoethanol (BME)) per liter of culture using one EDTA-free protease inhibitor tablet (Roche) per 50 mL lysis buffer. The cells were disrupted by four passes through a microfluidizer (30,000 psi) and centrifuged at 20,000 × *g* for 20 min. The lysate was filtered through a 0.45 µm syringe filter before loading onto a 5-mL Ni-NTA column equilibrated with five column volumes (CV) of wash buffer (20 mM Tris pH 8.0, 300 mM NaCl, 20 mM imidazole, 2.5 mM BME). The column was washed with 10 CV wash buffer, before the protein was eluted with 5 CV elution buffer (20 mM Tris pH 8.0, 300 mM NaCl, 300 mM imidazole, 2.5 mM BME). The elutate was dialyzed overnight with TEV protease against dialysis buffer (20 mM Tris pH 8.0, 150 mM NaCl, 2.5 mM BME). STARD proteins were separated from TEV protease and MBP by reverse Ni-NTA affinity column chromatography. The proteins were further purified over a Superdex 200 10/300 Increase gel filtration column equilibrated in 20 mM Tris pH 8.0, 100 mM NaCl, 2.5 mM BME.

### Expression of STARD2, STARD7, and STARD10 in human cell lines

HEK293S GnTI^-^ (ATCC #CRL-3022) and HepG2 (ATCC #HB-8065) cell lines stably expressing tetracycline repressor protein (TetR) were generated by lentiviral transduction with the TetR-HA-NLS-P2A-BSD-Myc transfer plasmid (Addgene plasmid no. 113899) and blasticidin selection (2 µg/mL)^50^. The cells were grown adherently in DMEM/F-12 medium supplemented with non-essential amino acids, 10% FBS, and blasticidin (2 µg/mL). gBlocks containing full-length STARD2, STARD7, or STARD10 with a C-terminal FLAG and His_8_ tag (sequences provided in Supplementary Table 6) were inserted into pHR-CMV-TetO_2_ plasmids featuring an IRES site and EmGFP reporter (Addgene plasmid no. 113884). The transfer plasmids were used for lentiviral transduction in conjunction with the envelope plasmid pMD2.G (Addgene plasmid no.12259) and packaging plasmid psPAX2 (Addgene plasmid no. 12260)^50^. After a week of culturing, the cells were sorted for GFP expression using FACS.

For protein expression in HEK293S TetR cells, the cells were suspension-adapted and grown in FreeStyle medium (Thermo Fisher Scientific) supplemented with 1% FBS. Protein expression was induced by the addition of tetracycline hydrochloride (1 µg/mL) to a 1 L culture after a cell density of 2–3 million cells/mL was reached. 24 h after induction, sodium butyrate was added (5 mM), and expression was allowed to proceed for another 24 h. Forty-eight hours after induction, the cells were harvested by centrifugation (300 × *g*) and washed twice with PBS.

For protein expression in HepG2 TetR cells, each cell line was expanded into twelve 150 mm dishes containing 25 mL complete media. At 80% confluency, protein expression was induced by the addition of tetracycline hydrochloride (1 µg/mL). After 24 h, 5 mM sodium butyrate was added, and the cells were harvested by scraping 48 h after induction. The cell pellet was washed twice with PBS.

Proteins were purified using anti-FLAG magnetic agarose beads (Thermo Fisher Scientific). Cell pellets were resuspended in 50 mL lysis buffer (50 mM HEPES pH 8.0, 150 mM NaCl, 5% glycerol) with a protease inhibitor tablet and stirred for 1 h. The cells were lyzed in a Potter-Elvehjem homogenizer, and cell debris was cleared by centrifugation at 10,000 × *g* for 10 min and 20,000 × *g* for 20 min. The supernatant was filtered through a 0.45 µm syringe filter before adding 250 µL magnetic beads that were previously washed with 2 × 1 mL lysis buffer. The suspension was placed on a roller for 1 h to allow protein binding to the beads. The supernatant was removed, and the beads were washed 3× with 5 mL and 10× with 1 mL lysis buffer (total wash volume: 25 mL). For elution, 0.5 mL of 1× FLAG peptide (DYKDDDDK; 1 mg/mL) in lysis buffer was added to the beads and placed on a roller for 30 min. The elution was repeated once. The resulting 1 mL eluate was concentrated to 100 µL using 10 kDa MWCO spin filters and washed twice by centrifugation with 0.5 mL lysis buffer to remove FLAG peptide. Proteins were purified on separate days from three consecutive passage numbers of the same cell lines for quantification of copurified lipids.

### Lipid extraction from purified proteins and LC-MS/MS lipidomics

Lipids were extracted from purified STARD2, STARD7(76–370), and STARD10 expressed in *E. coli* using methyl tert-butyl ether (MTBE)/methanol extraction^51^. Briefly, 100 µg of protein was diluted in 20 mM Tris-HCl buffer to a final volume of 40 µL. The samples were transferred into glass vials and mixed with 300 µL methanol and 1 mL MTBE. The solutions were incubated for 1 h at room temperature. Following incubation, 250 µL water was added, samples were vortexed and centrifuged at 400 × *g* for 10 min to induce phase separation. Eight hundred microliters of the upper phase were transferred into clean glass vials, and the lower phase was re-extracted with 400 µL of a 10:3 (v:v) MTBE:methanol mixture. The organic phases were combined, resulting in a total volume of 1.2 mL, and 800 µL were taken for LC-MS/MS analysis, adding 2 µL SPLASH Lipidomix (Avanti Research). Samples were dried in a centrifugal evaporator and resuspended in 100 µL buffer (80% lipidomics buffer A/20% lipidomics buffer B) for LC-MS/MS analysis.

Lipid extracts from purified proteins expressed in *E. coli* were analyzed by LC-MS/MS in negative ion mode. A Dionex UltiMate 3000 system equipped with a C18 column (Acclaim PepMap 100, C18, 75 μm × 15 cm, Thermo Scientific) was coupled to an Orbitrap Eclipse Tribrid mass spectrometer. Lipidomics buffer A contained ACN:H2O 60:40, 10 mM ammonium formate, 0.1% formic acid, and lipidomics buffer B consisted of isopropanol:ACN 90:10, 10 mM ammonium formate, 0.1% formic acid. During the 45 min chromatographic runs, the concentration of buffer B was increased with a gradient from 32% to 99%. Survey MS scans were acquired in the Orbitrap (*m*/*z* 300–2000) with a resolution of 120,000 @ *m*/*z* 200. The MS^2^ spectra were acquired with stepped HCD energies of 25 and 30 V. LC-MS/MS spectra were recorded in technical triplicate for each sample using Thermo XCalibur acquisition software (v. 4.6.67.17). The deprotonated internal standards PE 15:0/18:1(d7) (*m*/*z* 709.6; 5.3 μg/mL) and PG 15:0/18:1(d7) (*m*/*z* 740.5; 26.7 μg/mL) contained in the SPLASH Lipidomix mixture were used for relative lipid quantification. Files were converted using MSConvert (ProteoWizard v. 3.0)^52^ for lipid identification and chromatographic quantification using LipiDex^53^ (v. 1.1) in combination with MZmine 3^54^. Three technical replicates (*n* = 3) were measured for each lipid extract.

### Native mass spectrometry

Native MS was performed on an Orbitrap Eclipse Tribrid mass spectrometer (Thermo Fisher Scientific). Proteins were buffer-exchanged into native MS buffer (200 mM ammonium acetate pH 7.0) using Micro Bio-Spin P-6 gel columns (BioRad). Proteins were ionized by nano electrospray ionization using gold-coated borosilicate capillaries pulled in-house with a P97 Micropipette Puller (Sutter Instrument Corporation) and coated with an AgarAuto Sputter Coater.

STARD proteins were analyzed in Intact Protein mode using the Orbitrap for mass analysis (typically *m*/*z* 1000–6000, resolution = 15,000 @ *m*/*z* 200). Spectra were recorded using Thermo Xcalibur software (v. 4.6.67.17) and processed for further analysis in the Thermo XCalibur Qual Browser (v. 4.5.474.0). Spectra were deconvoluted in UniDec^55^ (v. 8.1.0) for intact mass determination. For the identification of copurified phospholipids, the intact protein–lipid complexes were isolated with a wide isolation window (usually 100 Th) in the ion trap (STARD2: *m*/*z* 2950 (10+); STARD7: *m*/*z* 3230 (12+); STARD10: *m*/*z* 3130 (12+), *m*/*z* 3760 (10−)) and activated using HCD (6–20% NCE). MS^2^ spectra of released lipids were recorded in the Orbitrap at high resolution (*m*/*z* 500–1000, resolution = 500,000 @ *m*/*z* 200). MS^2^ spectra of three biological replicates (*n* = 3) were acquired for proteins expressed in human cell lines. For proteins expressed in *E. coli*, three technical replicates (*n* = 3) were measured. Copurified lipids were quantified based on their relative intensities in the MS^2^ spectrum and normalized to the sum of intensities of all detected lipids within the same class. Different lipid classes were quantified separately to account for lipid class-specific response factors depending on the headgroup. For lipid identification, MS^3^ spectra were recorded in the ion trap using HCD (25–30% NCE) to identify lipid class and acyl chains. Lipid extracts were analyzed by direct infusion in Small Molecule mode. Mass spectra of lipid extracts were recorded in the Orbitrap at high resolution (500,000 @ *m*/*z* 200) in biological triplicate (*n* = 3) for human cell lines and in technical triplicate (*n* = 3) for bacterial lipid extracts.

For native top-down experiments, *apo* proteins were isolated in the ion trap and fragmented using HCD (70–100 V). Fragment spectra were recorded in the Orbitrap at high resolution (240,000 @ *m*/*z* 200). STARD10 was denatured by adding an equal volume of a solution containing 90% acetonitrile and 10% isopropanol with 2% formic acid. The 28+ charge state was isolated using a narrow isolation window (±1 Th) to select individual phosphorylation states. Isolated ions were fragmented using EThcD (Reaction time: 1.5 ms, 30% CE), and fragment spectra were recorded in the Orbitrap at high resolution (240,000 @ *m*/*z* 200). Fragment ions were assigned using precisION^48^ (v. 0.2.0) with a 3 ppm (post-calibration) matching tolerance.

### Lipid identification

Lipids that copurified with STARD2, STARD7, and STARD10 were identified based on MS^3^ HCD/HCD spectra. PC lipids were detected in positive ion mode and assigned through diagnostic headgroup fragments (*m*/*z* 184.07). Acyl chains were assigned based on low-intensity fragments resulting from neutral loss of each chain. PE and PG were detected in negative ion mode and assigned based on carboxylate fragments.

### Lipid quantification

PC species copurified with STARD2, STARD7, or STARD10 were compared relative to whole-cell lipid extracts from the corresponding cell pellets. Lipids were extracted from the cell pellets of bacterial and human cells obtained at the end of protein expression, following the Bligh-and-Dyer protocol^56^. Briefly, ca. 20 mg of the cell pellet was resuspended in 0.8 mL aqueous buffer (20 mM Tris pH 8.0, 150 mM NaCl) in a glass vial and mixed with 2 mL methanol and 1 mL chloroform. After sonication for 10 min, 1 mL chloroform and 1 mL of the buffer were added, and the mixture was vortexed for 30 s. After centrifugation at 1000 × *g* for 10 min, the lower (chloroform) phase was transferred into a clean glass vial. The solution was directly analyzed by MS.

We calculated the relative abundance of each PC species as the proportion of its peak intensity normalized to the total intensity of all detected PC species in the mass spectrum. For protein–lipid complexes, relative abundances were derived from MS² spectra, whereas MS¹ spectra were used for whole-cell lipid extracts. Enrichment or depletion of individual PC species in the copurified fractions was determined by subtracting their normalized abundance in the whole-cell extract from that in the corresponding protein-bound fraction. Statistical significance between copurified and whole-cell lipid samples was assessed using two-sided Welch’s t-tests performed on biological triplicates. Resulting *p*-values were adjusted for multiple hypothesis testing using the Benjamini–Hochberg false discovery rate correction.

To quantify changes in the acyl chain profiles of phospholipids in whole-cell lipid extracts, we extracted their intensities from high-resolution direct infusion mass spectra in triplicate and normalized within each lipid class. Data were first centered log-ratio (CLR) transformed to account for compositional constraints, and statistical significance was determined using one-way ANOVA, with pairwise comparisons by Tukey’s HSD. The adjusted p-values were used to determine statistical significance. For visualization of the results, bar charts were made in Origin 2025b, and correlation plots were created using Python 3.11.3.

### In vitro lipid binding assays

To determine lipid binding preferences in vitro, STARD2, 7, and 10 (20 µM) expressed in *E. coli* were incubated with a 1:1:1:1:1 mixture containing DOPC, DOPE, DOPG, DOPS, and DOPI (100 µM total lipid concentration) in 200 mM ammonium acetate containing 2xCMC C8E4. After 5 min incubation, the proteins were buffer-exchanged into 200 mM ammonium acetate using BioSpin-6 columns (BioRad) to remove excess lipids. Protein-lipid complexes were analyzed by native MS and MS^2^ experiments in positive and negative ion modes to release and assign bound lipids based on exact mass.

### siRNA-mediated protein knockdown

For knockdown of LTPs in HepG2 cells, we used ON-TARGETplus SMART pool siRNA specifically targeting STARD2, STARD7, and STARD10 (Dharmacon). The siRNA pools contained the following target sequences: STARD2: GGAAGUGGCAUUUCGUUCA, UGAUCCGGGUGAAGCAAUA, CGAUCGAGAGUGACGGCAA, CAAACUGGAUGACGUGCCA; STARD7: CAUCGAUUGUGCAGCGCUU, CCUCUGAGCGAAAGAACGA, UUAAUGAGAUGAAGCGGUU, CCAAUGUACUCACGGGAUU; STARD10: GGAAAGACUUGGUCCGAGC, GACAUUGAGUACCGCAAGA, AGAGCAGAGAGGAGCGGAU, GCCCGAUGACCAAGACUUU. HepG2 cells were seeded into 12-well plates at a seeding density of 10^5^ cells per well. After overnight incubation in complete medium (DMEM/F-12 medium supplemented with non-essential amino acids, 10% FBS), the medium was replaced by 1 mL of transfection solution containing siRNA at a final concentration of 50 nM and 4 µL DharmaFECT 4 transfection reagent per well. According to the manufacturer’s instructions, siRNA and transfection reagent were first incubated in a 200 µL volume for 20 min in serum-free medium before 800 µL complete medium was added. The transfections were performed in six replicates for each protein and control. Control cells were incubated with transfection reagent under identical conditions without adding siRNA. Cells were incubated with transfection solution at 37 °C with 5% CO_2_ for 72 h. Cells were washed twice with PBS, harvested by cell scraping, and prepared for lipid analysis and proteomics.

### Data-independent acquisition proteomics

For proteomics, ca. 6 × 10^5^ cells were lysed in 46 µL lysis buffer (5% SDS in 50 mM Tris-HCl, pH 7.4) through sonication. After clarification by centrifugation (13,000 × *g*, 8 min), the supernatant was transferred into a new tube. Two microliter of TCEP (120 mM in water) was added, and the reduction was allowed to proceed for 15 min at 55 °C. After incubation with 2 µL iodoacetamide (250 mM in 50 mM Tris-HCl) for 10 min at room temperature, the solution was acidified with 10 µL phosphoric acid (12% in water). 350 µL wash buffer (100 mM Tris-HCl in 90% methanol) was added, and the solution was applied to a miniprep column. Proteins were bound to the column by centrifugation (4000 × *g*, 30 s). The column was washed four times with 400 µL wash buffer through centrifugation and dried by a final centrifugation at 4000 × *g* for 1 min before being transferred into a new tube. 125 µL digestion buffer was applied to the column (50 mM Tris containing a total of 2 µg trypsin per column). Proteins were digested overnight at 37 °C. Peptides were eluted sequentially with 50 mM Tris, 0.2% formic acid in water, and 50% acetonitrile in water through centrifugation (4000 × *g*, 1 min; 80 µL per elution). The eluates were combined and dried in a centrifugal evaporator. The dried peptides were resuspended in 0.1% formic acid and diluted to a final concentration of 100 ng/µL.

Purified peptides were separated via nanoflow reversed-phase liquid chromatography (nanoElute 2, Bruker Daltonics) with a 30 min gradient. Peptides (50 ng, 0.5 µL injection volume) were loaded directly onto a 25 cm × 75 µm column packed with 1.7 µm C18 beads (pore size 120 Å; Aurora Ultimate CSI, IonOpticks) and separated at a flow rate of 250 nL/min and a column temperature of 50 °C. Mobile phase A consisted of 0.1% formic acid in water, and mobile phase B consisted of 0.1% formic acid in acetonitrile (v/v; Fisher Scientific, LC-MS grade). The gradient was as follows: 2–23% B over 18 min, 23–35% B over 4 min, and 35–90% B over 4 min, followed by a 4-min wash at 90% B. Eluting peptides were infused into a TIMS quadrupole time-of-flight mass spectrometer (timsTOF Ultra, Bruker Daltonics) equipped with an electrospray ion source (CaptiveSpray, Bruker Daltonics). Spectra were acquired with the Bruker HyStar software (v. 6.3). Source parameters were set to a capillary voltage of 4500 V, a dry gas flow of 3.0 L min⁻¹, and a dry gas temperature of 180 °C. Data were acquired in dia-PASEF mode over an *m*/*z* range of 400–1000 and an ion mobility range of 1/K₀ = 0.64–1.45 Vs/cm^2^ (Supplementary Table 5). The TIMS analyzer was operated at a 100% duty cycle with 100 ms accumulation and 100 ms ramp times (acquisition cycle time = 0.96 s). Collision energies were linearly stepped from 20 to 59 eV as a function of increasing ion mobility.

dia-PASEF raw files were processed using the “DIA_SpecLib_Quant_diaPASEF” workflow in FragPipe^57,58^ (v. 23.1). Pseudo-MS/MS spectra were generated using diaTRACER^59^ (v. 1.3.3) and searched using MSFragger^60^ (v. 4.3). Searches were performed against the Homo sapiens UniProt reference proteome (UP000005640; reviewed entries only) supplemented with common contaminants and decoys (downloaded November 2025). Precursor and fragment mass tolerances were set to 20 ppm, and the isotope error window was set to 0/1/2. Mass calibration and parameter optimization were enabled. Enzymatic specificity was set to “stricttrypsin”, allowing up to two missed cleavages. Carbamidomethylation of cysteine was specified as a fixed modification, while methionine oxidation, protein N-terminal acetylation, and pyro-Glu/Gln formation at peptide N-termini were included as variable modifications (maximum of three variable modifications per peptide). MSBooster and Percolator were used to predict retention time and MS/MS spectra, and to rescore peptide–spectrum matches (PSMs). PSMs were filtered to a 1% false-discovery rate (FDR) and combined with the pseudo-MS/MS spectra using EasyPQP (v.0.1.52) to generate a spectral library for DIA quantification (139,376 precursors mapping to 8,002 protein groups).

dia-PASEF runs were quantified using DIA-NN^61^ (v.1.8.2 beta 8) with the spectral library generated in FragPipe. Mass accuracy settings for MS1 and MS2, as well as the scan window, were determined automatically by DIA-NN based on the first run in the experiment. Precursor and protein group identifications were filtered at 1% FDR. Protein inference was disabled, retaining protein group assignments from the input spectral library. Quantification was performed using fixed-width peak-center integration. Cross-run normalization was performed using DIA-NN’s default RT-dependent method, and protein group quantities were calculated using the MaxLFQ algorithm. Quantification matrices for precursors, protein groups, and gene groups were exported with 1% FDR filtering. The abundance of STARD2, STARD7, and STARD10 was quantified in six biological replicates (*n* = 6) per condition (control and three siRNA knockdown conditions). *p*-values were determined by pairwise Welch’s t-test between the control sample and each knockdown condition, using Bonferroni correction to correct for multiple testing.

### Quantitative phosphoproteomics

Proteomics analysis of purified STARD2, STARD7, and STARD10 expressed in HEK293S cells was performed after tryptic digestion of Coomassie-stained gel slices. Briefly, the gel pieces were dehydrated and washed, then treated with 10 mM DTT in 25 mM ammonium bicarbonate for 1 h at 56 °C, followed by a 45 min reaction at room temperature in the dark with 55 mM iodoacetamide in 25 mM ammonium bicarbonate. After washing, the gel pieces were incubated overnight at 37 °C with 25 ng/µL trypsin in 25 mM ammonium bicarbonate. The peptide-containing supernatant and wash were combined and dried in a centrifugal evaporator. The peptides were redissolved by sonication in 20 µL LC-MS grade water containing 0.1% formic acid.

Purified peptides were separated by nanoflow reversed-phase HPLC (Dionex UltiMate 3000 system) with a 60 min gradient. Peptides (1 µL) were loaded onto a 75 µm × 2 cm pre-column, followed by an analytical C18 column (Acclaim PepMap 100, C18, 75 µm × 15 cm, Thermo Scientific), which was coupled to an Orbitrap Eclipse Tribrid mass spectrometer. Mobile phase A consisted of 0.1% formic acid in water, and mobile phase B consisted of 80% acetonitrile, 20% water, and 0.1% formic acid (v/v, Fisher Scientific, LC-MS grade). Buffer B was gradually increased from 5 to 40% over 45 min, followed by 40 to 99% over 5 min, where it was maintained for an additional 5 min. The mass spectrometer was operated in data-dependent acquisition mode including charge states 2–5 and with an exclusion duration of 30 s. Full MS scans were recorded from *m*/*z* 300–2000 at a resolution of 120,000, and MS^2^ scans were recorded with HCD (30% NCE) at a resolution of 30,000. One LC-MS/MS run was performed per sample (*n* = 1). Data were recorded using Thermo XCalibur software (v. 4.6.67.17) and analyzed using Fragpipe (v. 23.1) with quantification from IonQuant^60^.

### Structure prediction and system setup for molecular dynamics simulations

All wildtype structures were predicted using Boltz-2^40^, Chai-1^62^, and Openfold3^63^ with or without incorporating available crystallographic structures as templates to guide the predictions. The inclusion of DOPC, SAPC, POPC, or acetate as a ligand was achieved by adding the respective SMILES representation as input. Mutant structures were generated from the predicted wildtype structures by converting the conserved arginine into a glutamine using Chimera^64^. To predict the structure of STARD2, the full-length sequence from UniProt accession Q9UKL6 was used as input, supplying PDB ID: 1LN1 as a structural template. For STARD10, the full-length sequence from UniProt accession Q9Y365 and the PDB ID: 6SER were used as sequence input and structural template, respectively. For STARD7, Uniprot: Q9NQZ5, the sequence was truncated to begin from residue number 76 to account for cleavage of the mitochondrial localization signal. To account for the influence of a bound lipid, additional predictions for STARD7 and STARD10 were generated using PDB ID: 1LN1 as a structural template when permitted by the prediction tool. The respective mutants for STARD2, STARD7, and STARD10 were R78Q, R189Q (in reference to the full-length numbering of the residues), and R92Q. The predictions’ confidence scores were 0.957, 0.799, and 0.855 for the DOPC-bound wild-type STARD2, STARD7, and STARD10, respectively. To build each system, the predicted DOPC-bound protein was placed in a 10 nm cubic box, which was then solvated with water and a 0.15 M concentration of NaCl.

### Molecular dynamics simulations

The CHARMM36m force field^65^ was used in combination with the GROMACS (v 2023.3) package^66^. The topology was generated using GROMACS pdb2gmx. The systems were equilibrated following the CHARMM-GUI six-step protocol^67^. Each system was initially minimized for 5000 steps. Next, two equilibrations in the NVT ensemble were run for 125 ps, followed by four equilibrations in the NPT ensemble, gradually removing the constraints on the protein backbone and lipid head. For the production runs, a time step of 2 fs was used with the md integrator. Three independent replicas were simulated for 2 μs each. Temperature was kept at 310 K using a V-rescale thermostat^68^, while pressure was maintained at 1 bar using an isotropic C-rescale barostat^69^. A Verlet cutoff scheme with a cutoff value of 1.2 nm was used to calculate van der Waals and Coulombic interactions. Beyond 1.2 ns, particle mesh Ewald was used to compute long-range interactions. Hydrogen bonds were constrained using the LINCS algorithm^70^.

### Simulation analysis

To calculate the Root Mean Square Fluctuation (RMSF) of lipids in the protein cavities, GROMACS’s gmx rmsf was used, omitting the hydrogens to reduce noise. To calculate the minimum distance between the phosphate atom and the arginine/glutamine residue, and the choline N group and each aromatic residue, gmx mindist was used. gmx distance was used to calculate the distance between the center of mass (COM) of the choline headgroup and the COM of the aromatic cages of STARD2 and STARD7. The aromatic cage residues of STARD2 were W101, Y114, Y116, and Y155^22^. The aromatic cage residues of STARD7 were W215, Y228, Y230, and Y267, as informed by pairwise sequence alignment. The line plots show the average values from the three replicas for each system, with the shaded region showing the standard mean of error with respect to each individual replica. The probability density plots were calculated for only the last 1800 ns of the simulations’ durations, using Matplotlib’s histogram function. All graphical plots were generated using Matplotlib^71^ (v. 3.10.3). All visual representations were created using VMD^72^ (v. 1.9.3). A reliability and reproducibility checklist for molecular dynamics simulations is provided in Supplementary Table 7.

### Desphosphorylation of STARD2 and STARD10

To dephosphorylate STARD2 and STARD10 expressed in HEK293S cells, 20 µL of the purified protein (90 µM) was incubated with 0.8 µL λ protein phosphatase (New England Biolabs) and 2 µL MnCl_2_ (10 mM) in the presence of 1 mM DTT. For dephosphorylation of STARD2 and partial dephosphorylation of STARD10, the reaction mixture was incubated for 1 h at 30 °C before buffer exchange into ammonium acetate for native MS. To completely dephosphorylate of STARD10, the incubation time was increased to 4 h at room temperature. The experiment was performed once (*n* = 1).

### Liposome preparation for lipid transfer assay

Protein-mediated lipid transfer between two populations of liposomes was measured based on Förster resonance energy transfer between NBD-PC and Rh-PE^73,74^. Acceptor liposomes were generated from dried HEK293S lipid extract previously characterized by direct infusion MS. For the generation of donor liposomes, we determined the mass of the dried lipid extract and added NBD-PC and Rh-PE to a final concentration of 1 and 5 mol%, respectively (assuming an average molecular weight of 750 g/mol). The lipid film supplemented with fluorescent lipids was redissolved in chloroform, vortexed, and dried again. The dried lipid films were reconstituted in warm (37 °C) liposome buffer (20 mM Tris pH 8.0, 150 mM NaCl) at a final lipid concentration of 500 nmol/mL. Liposomes were generated by extrusion of the suspension through a mini extruder (Avanti Research) using polycarbonate membranes with pore sizes decreasing from 400 nm to 200 nm and finally 100 nm, resulting in transparent liposome solutions.

### Lipid transfer assay

The lipid transfer assay was performed in a 96-well plate at 25 °C in a total volume of 200 µL using a plate reader (CLARIOstar; BMG Labtech). Data were acquired using BMG Labtech CLARIOstar software version 5.60 R2. The excitation wavelength was 464 ± 5 nm, and emission was measured at 530 ± 5 nm. Fluorescence was measured in time steps of 15 s, after initial shaking of the plates for 10 s. Control measurements were performed in triplicate for solutions containing either 15 µL donor liposomes, 30 µL acceptor liposomes, or 15 µL donor liposomes with 30 µL acceptor liposomes with and without 1% Triton-X. The average fluorescence measured with the addition of Triton-X was set as the maximum fluorescence. To measure the lipid transfer activity of STARD proteins, 5 µL of the protein (10 µM; final concentration = 0.25 µM) was mixed with 15 µL donor liposomes in 150 µL liposome buffer. 30 µL acceptor liposomes were added and the measurement was started 30 s after the addition. To measure the initial lipid transfer kinetics for NBD-PC with different acyl chains, the proteins were added at reduced concentrations: STARD2 0.05 µM, STARD7 0.25 µM, STARD10 0.10 µM. All measurements were performed in triplicate, and the data were normalized against the maximum fluorescence. The same liposome batches were used for all measurements, except for the acyl chain-selective transport assay (Fig. 6b). For this experiment, separate batches of donor liposomes containing NBD-PC carrying either 16:0 or 18:1 acyl chains were prepared in parallel. Fluorescence time courses were analyzed by nonlinear regression using a single-exponential model describing a first-order approach to equilibrium:

$$I(t)={I}_{0}+({I}_{\infty }-{I}_{0})({1-e}^{-{kt}})$$

where *I*(*t*) is the relative fluorescence intensity at time *t*, *I*_*0*_ is the initial relative fluorescence, *I*_*∞*_ is the asymptotic relative fluorescence at equilibrium, and *k* is the apparent first-order rate constant. Each replicate was fitted independently using nonlinear least-squares regression (scipy.optimize.curve_fit) implemented in Python (v. 3.11.3) and plotted in Origin 2025b. For each condition, rate constants were obtained from three independent measurements and are reported as mean ± standard deviation. Statistical differences between conditions were assessed using Welch’s two-sample t-test applied to the fitted rate constants.

### Lipid exchange assay

STARD10 expressed in HEK293S cells was dephosphorylated using λ protein phosphatase (New England Biolabs) as described above (100 µM STARD10 in 20 µL with 10 mM MnCl_2_ and 1 mM DTT, 2 h at 30 °C). A control sample was incubated with MnCl_2_ and DTT under identical conditions but in the absence of λ protein phosphatase. After buffer exchange into ammonium acetate (200 mM), the degree of phosphorylation was determined for both samples by native MS, using in-source activation (120–130 V) to remove bound lipids. The samples were diluted to 20 µM protein concentration and incubated 1:1 (v:v) with a solution of liposomes containing deuterated PC (1.5 mM). The liposomes were prepared by resuspension of a lipid film consisting of 80% PC 16:0-d31/18:1 and 20% cholesterol in ammonium acetate (200 mM), followed by liposome extrusion as described above. Liposomes were characterized by MS, showing that the main species were protonated PC 16:0-d31/18:1 (*m*/*z* 791.78) and PC 16:0-d30/18:1 (*m*/*z* 790.78), derived from hydrogen-deuterium exchange in solution. STARD10 was incubated with the liposome solution at room temperature, and samples were taken for native MS after 10, 20, and 30 min. MS^2^ spectra were recorded in positive ion mode by ion trap isolation (*m*/*z* 3130 (12+) for phosphorylated STARD10, *m*/*z* 3115 (12+) for dephosphorylated STARD10; 80 Th isolation window), followed by the release of bound lipids using HCD (15% NCE). Lipid exchange was quantified by comparing the relative abundance of protonated PC 16:0-d30/18:1 (*m*/*z* 790.78) to protonated PC 36:2 (*m*/*z* 786.60), the most abundant endogenous lipid. The incubation and mass spectrometry analyses were performed in three independent replicates. Statistical significance was assessed using Welch’s t-test.

### Reporting summary

Further information on research design is available in the Nature Portfolio Reporting Summary linked to this article.

## Supplementary information

Supplementary Information

Reporting Summary

Transparent Peer Review File

## Source data

Source data

## Data Availability

Supplementary Figs. and tables are provided in the Supplementary Information. Raw files generated in this study have been deposited in the Figshare repository under DOI: 10.6084/m9.figshare.31056178 [https://doi.org/10.6084/m9.figshare.31056178]. The lipidomics data generated in this study have been deposited to the MassIVE database under accession code MSV000102576. Proteomics data generated in this study have been deposited to the ProteomeXchange Consortium via the PRIDE^75^ partner repository under accession code PXD072869. Source data are provided with this paper.

## References

[1] van Meer, G., Voelker, D. R. & Feigenson, G. W. Membrane lipids: where they are and how they behave. *Nat. Rev. Mol. Cell Biol.* **9**, 112–124 (2008). 18216768 10.1038/nrm2330 PMC2642958

[2] Reinisch, K. M., De Camilli, P. & Melia, T. J. Lipid dynamics at membrane contact sites. *Annu. Rev. Biochem.* **94**, 479–502 (2025). 40067957 10.1146/annurev-biochem-083024-122821

[3] Wong, L. H., Copic, A. & Levine, T. P. Advances on the transfer of lipids by lipid transfer proteins. *Trends Biochem. Sci.* **42**, 516–530 (2017). 28579073 10.1016/j.tibs.2017.05.001 PMC5486777

[4] Wong, L. H., Gatta, A. T. & Levine, T. P. Lipid transfer proteins: the lipid commute via shuttles, bridges and tubes. *Nat. Rev. Mol. Cell Biol.* **20**, 85–101 (2019). 30337668 10.1038/s41580-018-0071-5

[5] Reinisch, K. M. & Prinz, W. A. Mechanisms of nonvesicular lipid transport. *J. Cell Biol.* **220**, e202012058 (2021). 33605998 10.1083/jcb.202012058 PMC7901144

[6] Pemberton, J. G., Kim, Y. J. & Balla, T. Integrated regulation of the phosphatidylinositol cycle and phosphoinositide-driven lipid transport at ER-PM contact sites. *Traffic* **21**, 200–219 (2020). 31650663 10.1111/tra.12709

[7] Lipp, N. F., Ikhlef, S., Milanini, J. & Drin, G. Lipid exchangers: cellular functions and mechanistic links with phosphoinositide metabolism. *Front. Cell Dev. Biol.* **8**, 663 (2020). 32793602 10.3389/fcell.2020.00663 PMC7385082

[8] Alpy, F. & Tomasetto, C. START ships lipids across interorganelle space. *Biochimie* **96**, 85–95 (2014). 24076129 10.1016/j.biochi.2013.09.015

[9] Clark, B. J. The START-domain proteins in intracellular lipid transport and beyond. *Mol. Cell Endocrinol.* **504**, 110704 (2020). 31927098 10.1016/j.mce.2020.110704

[10] Kanno, K., Wu, M. K., Scapa, E. F., Roderick, S. L. & Cohen, D. E. Structure and function of phosphatidylcholine transfer protein (PC-TP)/StarD2. *Biochim. Biophys. Acta* **1771**, 654–662 (2007). 17499021 10.1016/j.bbalip.2007.04.003 PMC2743068

[11] Horibata, Y. & Sugimoto, H. StarD7 mediates the intracellular trafficking of phosphatidylcholine to mitochondria. *J. Biol. Chem.* **285**, 7358–7365 (2010). 20042613 10.1074/jbc.M109.056960 PMC2844184

[12] Haberkant, P. et al. In vivo profiling and visualization of cellular protein-lipid interactions using bifunctional fatty acids. *Angew. Chem. Int. Ed.* **52**, 4033–4038 (2013). 10.1002/anie.201210178 23450850

[13] Roderick, S. L. et al. Structure of human phosphatidylcholine transfer protein in complex with its ligand. *Nat. Struct. Biol.* **9**, 507–511 (2002). 12055623 10.1038/nsb812

[14] Bockelmann, S. et al. A search for ceramide binding proteins using bifunctional lipid analogs yields CERT-related protein StarD7. *J. Lipid Res.* **59**, 515–530 (2018). 29343537 10.1194/jlr.M082354 PMC5832928

[15] Olayioye, M. A. et al. StarD10, a START domain protein overexpressed in breast cancer, functions as a phospholipid transfer protein. *J. Biol. Chem.* **280**, 27436–27442 (2005). 15911624 10.1074/jbc.M413330200

[16] Titeca, K. et al. Systematic analyses of lipid mobilization by human lipid transfer proteins. *Nature* **651**, 511–520 (2026). 41501472 10.1038/s41586-025-10040-y PMC12979188

[17] Saita, S. et al. PARL partitions the lipid transfer protein STARD7 between the cytosol and mitochondria. *EMBO J*. **37**, e97909 (2018). 10.15252/embj.201797909 PMC5813258 29301859

[18] Horibata, Y. et al. Identification of the N-terminal transmembrane domain of StarD7 and its importance for mitochondrial outer membrane localization and phosphatidylcholine transfer. *Sci. Rep.* **7**, 8793 (2017). 28821867 10.1038/s41598-017-09205-1 PMC5562819

[19] Saita, S. et al. PARL mediates Smac proteolytic maturation in mitochondria to promote apoptosis. *Nat. Cell Biol.* **19**, 318–328 (2017). 28288130 10.1038/ncb3488

[20] Kasurinen, J., Van Paridon, P. A., Wirtz, K. W. & Somerharju, P. Affinity of phosphatidylcholine molecular species for the bovine phosphatidylcholine and phosphatidylinositol transfer proteins. Properties of the sn-1 and sn-2 acyl binding sites. *Biochemistry* **29**, 8548–8554 (1990). 2271538 10.1021/bi00489a007

[21] Feng, L., Chan, W. W., Roderick, S. L. & Cohen, D. E. High-level expression and mutagenesis of recombinant human phosphatidylcholine transfer protein using a synthetic gene: evidence for a C-terminal membrane binding domain. *Biochemistry* **39**, 15399–15409 (2000). 11112525 10.1021/bi001076a

[22] Talandashti, R., Moqadam, M. & Reuter, N. Model mechanism for lipid uptake by the human STARD2/PC-TP phosphatidylcholine transfer protein. *J. Phys. Chem. Lett.* **15**, 8287–8295 (2024). 39143857 10.1021/acs.jpclett.4c01743 PMC11331517

[23] de Brouwer, A. P. et al. Clofibrate-induced relocation of phosphatidylcholine transfer protein to mitochondria in endothelial cells. *Exp. Cell Res.* **274**, 100–111 (2002). 11855861 10.1006/excr.2001.5460

[24] Olayioye, M. A. et al. The phosphoprotein StarD10 is overexpressed in breast cancer and cooperates with ErbB receptors in cellular transformation. *Cancer Res.* **64**, 3538–3544 (2004). 15150109 10.1158/0008-5472.CAN-03-3731

[25] Hoffmann, P. et al. Breast cancer protein StarD10 identified by three-dimensional separation using free-flow electrophoresis, reversed-phase high-performance liquid chromatography, and sodium dodecyl sulfate-polyacrylamide gel electrophoresis. *Electrophoresis* **26**, 1029–1037 (2005). 15704244 10.1002/elps.200406197

[26] Olayioye, M. A. et al. Phosphorylation of StarD10 on serine 284 by casein kinase II modulates its lipid transfer activity. *J. Biol. Chem.* **282**, 22492–22498 (2007). 17561512 10.1074/jbc.M701990200

[27] Hamai, A. & Drin, G. Specificity of lipid transfer proteins: an in vitro story. *Biochimie* **227**, 85–110 (2024). 39304019 10.1016/j.biochi.2024.09.007

[28] Saliba, A. E., Vonkova, I. & Gavin, A. C. The systematic analysis of protein-lipid interactions comes of age. *Nat. Rev. Mol. Cell Biol.* **16**, 753–761 (2015). 26507169 10.1038/nrm4080

[29] Kamp, H. et al. Specificity of the phosphatidylcholine exchange protein from bovine liver. *Biochemistry* **16**, 1310–1316 (1977). 557337 10.1021/bi00626a011

[30] Somerharju, P. J., Van Loon, D. & Wirtz, K. W. Determination of the acyl chain specificity of the bovine liver phosphatidylcholine transfer protein. Application of pyrene-labeled phosphatidylcholine species. *Biochemistry* **26**, 7193–7199 (1987). 3427069 10.1021/bi00396a048

[31] de Brouwer, A. P. et al. The binding of phosphatidylcholine to the phosphatidylcholine transfer protein: affinity and role in folding. *Chem. Phys. Lipids* **112**, 109–119 (2001). 11551535 10.1016/s0009-3084(01)00171-2

[32] Carrat, G. R. et al. The type 2 diabetes gene product STARD10 is a phosphoinositide-binding protein that controls insulin secretory granule biogenesis. *Mol. Metab.* **40**, 101015 (2020). 32416313 10.1016/j.molmet.2020.101015 PMC7322359

[33] Zhang, J. et al. De novo labeling and trafficking of individual lipid species in live cells. *Mol. Metab.* **61**, 101511 (2022). 35504533 10.1016/j.molmet.2022.101511 PMC9114690

[34] Peng, T., Yuan, X. & Hang, H. C. Turning the spotlight on protein-lipid interactions in cells. *Curr. Opin. Chem. Biol.* **21**, 144–153 (2014). 25129056 10.1016/j.cbpa.2014.07.015 PMC4206213

[35] Maeda, K., Poletto, M., Chiapparino, A. & Gavin, A. C. A generic protocol for the purification and characterization of water-soluble complexes of affinity-tagged proteins and lipids. *Nat. Protoc.* **9**, 2256–2266 (2014). 25167057 10.1038/nprot.2014.148

[36] Geijtenbeek, T. B., Westerman, J., Heerma, W. & Wirtz, K. W. Phosphatidylcholine transfer protein from bovine liver contains highly unsaturated phosphatidylcholine species. *FEBS Lett.* **391**, 333–335 (1996). 8765001 10.1016/0014-5793(96)00770-3

[37] Iglesias-Artola, J. M. et al. Quantitative imaging of lipid transport in mammalian cells. *Nature* **646**, 474–482 (2025). 40836094 10.1038/s41586-025-09432-x PMC12507682

[38] Kirschbaum, C. et al. Following phospholipid transfer through the OmpF(3)-MlaA-MlaC lipid shuttle with native mass spectrometry. *Proc. Natl. Acad. Sci. USA* **122**, e2420041122 (2025). 40168124 10.1073/pnas.2420041122 PMC12002339

[39] Oursel, D. et al. Lipid composition of membranes of *Escherichia coli* by liquid chromatography/tandem mass spectrometry using negative electrospray ionization. *Rapid Commun. Mass Spectrom.* **21**, 1721–1728 (2007). 17477452 10.1002/rcm.3013

[40] Passaro, S. et al. Boltz-2: towards accurate and efficient binding affinity prediction. Preprint at *bioRxiv*, 10.1101/2025.06.14.659707 (2025).

[41] Symons, J. L. et al. Lipidomic atlas of mammalian cell membranes reveals hierarchical variation induced by culture conditions, subcellular membranes, and cell lineages. *Soft Matter* **17**, 288–297 (2021). 32451522 10.1039/d0sm00404a PMC7688498

[42] Morita, S. Y. & Ikeda, Y. Regulation of membrane phospholipid biosynthesis in mammalian cells. *Biochem. Pharmacol.* **206**, 115296 (2022). 36241095 10.1016/j.bcp.2022.115296

[43] Kapushesky, M. et al. Gene expression atlas at the European Bioinformatics Institute. *Nucleic Acids Res* **38**, D690–D698 (2010). 19906730 10.1093/nar/gkp936 PMC2808905

[44] Gorden, D. L. et al. Biomarkers of NAFLD progression: a lipidomics approach to an epidemic. *J. Lipid Res.* **56**, 722–736 (2015). 25598080 10.1194/jlr.P056002 PMC4340319

[45] Fahy, E. Biomarkers of NAFLD progression: a lipidomics approach to an epidemic. Part 1: Liver (2018). Metabolomics Workbench. Study ID: ST000915. Project 10.21228/M8V961 PMC4340319 25598080

[46] Koay, Y. C. Metabolomics studies on human cardiac samples. Metabolomics Workbench. Study ID: ST003104. Project 10.21228/M8N42X (2023).

[47] Hunter, B. Lipidomics analysis within healthy donors and end-stage heart failure with a focus on ischaemic cardiomyopathy with diabetes. Metabolomics Workbench. Study ID: ST003275. Project 10.21228/M83529 (2024).

[48] Bennett, J. L. et al. Uncovering hidden protein modifications with native top-down mass spectrometry. *Nat. Methods* **22**, 2127–2137 (2025). 41023435 10.1038/s41592-025-02846-5 PMC12510877

[49] Horibata, Y. et al. StarD7 protein deficiency adversely affects the phosphatidylcholine composition, respiratory activity, and cristae structure of mitochondria. *J. Biol. Chem.* **291**, 24880–24891 (2016). 27694445 10.1074/jbc.M116.736793 PMC5122760

[50] Elegheert, J. et al. Lentiviral transduction of mammalian cells for fast, scalable and high-level production of soluble and membrane proteins. *Nat. Protoc.* **13**, 2991–3017 (2018). 30455477 10.1038/s41596-018-0075-9 PMC6364805

[51] Wu, D. et al. Native MS-guided lipidomics to define endogenous lipid microenvironments of eukaryotic receptors and transporters. *Nat. Protoc.* **20**, 1–25 (2025). 39174660 10.1038/s41596-024-01037-4

[52] Chambers, M. C. et al. A cross-platform toolkit for mass spectrometry and proteomics. *Nat. Biotechnol.* **30**, 918–920 (2012). 23051804 10.1038/nbt.2377 PMC3471674

[53] Hutchins, P. D., Russell, J. D. & Coon, J. J. LipiDex: an integrated software package for high-confidence lipid identification. *Cell Syst.* **6**, 621–625 e625 (2018). 29705063 10.1016/j.cels.2018.03.011 PMC5967991

[54] Schmid, R. et al. Integrative analysis of multimodal mass spectrometry data in MZmine 3. *Nat. Biotechnol.* **41**, 447–449 (2023). 36859716 10.1038/s41587-023-01690-2 PMC10496610

[55] Marty, M. T. et al. Bayesian deconvolution of mass and ion mobility spectra: from binary interactions to polydisperse ensembles. *Anal. Chem.* **87**, 4370–4376 (2015). 25799115 10.1021/acs.analchem.5b00140 PMC4594776

[56] Bligh, E. G. & Dyer, W. J. A rapid method of total lipid extraction and purification. *Can. J. Biochem. Physiol.* **37**, 911–917 (1959). 13671378 10.1139/o59-099

[57] Yu, F. et al. Analysis of DIA proteomics data using MSFragger-DIA and FragPipe computational platform. *Nat. Commun.* **14**, 4154 (2023). 37438352 10.1038/s41467-023-39869-5 PMC10338508

[58] Demichev, V. et al. dia-PASEF data analysis using FragPipe and DIA-NN for deep proteomics of low sample amounts. *Nat. Commun.* **13**, 3944 (2022). 35803928 10.1038/s41467-022-31492-0 PMC9270362

[59] Li, K., Teo, G. C., Yang, K. L., Yu, F. & Nesvizhskii, A. I. diaTracer enables spectrum-centric analysis of diaPASEF proteomics data. *Nat. Commun.* **16**, 95 (2025). 39747075 10.1038/s41467-024-55448-8 PMC11696033

[60] Kong, A. T., Leprevost, F. V., Avtonomov, D. M., Mellacheruvu, D. & Nesvizhskii, A. I. MSFragger: ultrafast and comprehensive peptide identification in mass spectrometry-based proteomics. *Nat. Methods* **14**, 513–520 (2017). 28394336 10.1038/nmeth.4256 PMC5409104

[61] Demichev, V., Messner, C. B., Vernardis, S. I., Lilley, K. S. & Ralser, M. DIA-NN: neural networks and interference correction enable deep proteome coverage in high throughput. *Nat. Methods* **17**, 41–44 (2020). 31768060 10.1038/s41592-019-0638-x PMC6949130

[62] Chai Discovery Team. et al. Chai-1: decoding the molecular interactions of life. Preprint at *bioRxiv*, 10.1101/2024.10.10.615955 (2024).

[63] Ahdritz, G. et al. OpenFold: retraining AlphaFold2 yields new insights into its learning mechanisms and capacity for generalization. *Nat. Methods* **21**, 1514–1524 (2024). 38744917 10.1038/s41592-024-02272-z PMC11645889

[64] Pettersen, E. F. et al. UCSF Chimera-a visualization system for exploratory research and analysis. *J. Comput. Chem.* **25**, 1605–1612 (2004). 15264254 10.1002/jcc.20084

[65] Huang, J. et al. CHARMM36m: an improved force field for folded and intrinsically disordered proteins. *Nat. Methods* **14**, 71–73 (2017). 27819658 10.1038/nmeth.4067 PMC5199616

[66] Van Der Spoel, D. et al. GROMACS: fast, flexible, and free. *J. Comput. Chem.* **26**, 1701–1718 (2005). 16211538 10.1002/jcc.20291

[67] Jo, S., Kim, T., Iyer, V. G. & Im, W. CHARMM-GUI: a web-based graphical user interface for CHARMM. *J. Comput. Chem.* **29**, 1859–1865 (2008). 18351591 10.1002/jcc.20945

[68] Bussi, G., Donadio, D. & Parrinello, M. Canonical sampling through velocity rescaling. *J. Chem. Phys.* **126**, 014101 (2007). 17212484 10.1063/1.2408420

[69] Bernetti, M. & Bussi, G. Pressure control using stochastic cell rescaling. *J. Chem. Phys.* **153**, 114107 (2020). 32962386 10.1063/5.0020514

[70] Hess, B., Bekker, H., Berendsen, H. J. C. & Fraaije, J. G. E. M. LINCS: A linear constraint solver for molecular simulations. *J. Comput. Chem.* **18**, 1463–1472 (1997).

[71] Hunter, J. D. Matplotlib: a 2D graphics environment. *Comput. Sci. Eng.* **9**, 90–95 (2007).

[72] Humphrey, W., Dalke, A. & Schulten, K. VMD: visual molecular dynamics. *J. Mol. Graph.* **14**, 33–38–27–38 (1996). 8744570 10.1016/0263-7855(96)00018-5

[73] Nichols, J. W. & Pagano, R. E. Use of resonance energy transfer to study the kinetics of amphiphile transfer between vesicles. *Biochemistry* **21**, 1720–1726 (1982). 7082641 10.1021/bi00537a003

[74] Nichols, J. W. & Pagano, R. E. Resonance energy transfer assay of protein-mediated lipid transfer between vesicles. *J. Biol. Chem.* **258**, 5368–5371 (1983). 6853520

[75] Perez-Riverol, Y. et al. The PRIDE database at 20 years: 2025 update. *Nucleic Acids Res.* **53**, D543–D553 (2025). 39494541 10.1093/nar/gkae1011 PMC11701690

[76] Abramson, J. et al. Accurate structure prediction of biomolecular interactions with AlphaFold 3. *Nature* **630**, 493–500 (2024). 38718835 10.1038/s41586-024-07487-w PMC11168924

