# Supplementary material for: Differential lipid selectivity of StARD phospholipid transporters revealed by native MS
Source: Nat Commun. 2026 Aug 22;17:10054. doi: 10.1038/s41467-026-76988-1 (PMC13594186; doi:10.1038/s41467-026-76988-1)
Supplement: Supplementary file 1 [file 41467_2026_76988_MOESM1_ESM.pdf]

Supplementary Information for:

Differential lipid selectivity of StARD phospholipid transporters revealed by native MS

Carla Kirschbaum[1,2,6]*, Sophie A. S. Lawrence[1,2], Jack L. Bennett[1,2], Olivia B. Ramsay[1,3], Yara Ahmed[4], Tarick J. El-Baba[1,2], Stefano Vanni[4,5], Carol V. Robinson[1,2]*

1 Kavli Institute for Nanoscience Discovery, University of Oxford, Oxford OX1 3QU, United Kingdom
2 Department of Chemistry, University of Oxford, Oxford OX1 3QZ, United Kingdom
3 Department of Oncology, University of Oxford, Oxford OX3 7DQ, United Kingdom
4 Department of Biology, University of Fribourg, Fribourg, Switzerland
5 Swiss National Center for Competence in Research Bio-inspired Materials, University of Fribourg, Fribourg, Switzerland
6 Institute of Biochemistry, Charité – University Medicine Berlin, Berlin, Germany

* Correspondence: carla.kirschbaum@charite.de, carol.robinson@chem.ox.ac.uk

Contents

Supplementary Figures 2
Supplementary Figure 1. 2
Supplementary Figure 2. 2
Supplementary Figure 3. 3
Supplementary Figure 4. 4
Supplementary Figure 5. 5
Supplementary Figure 6. 6
Supplementary Figure 7. 7
Supplementary Figure 8. 8
Supplementary Figure 9. 9
Supplementary Figure 10. 10
Supplementary Figure 11 10
Supplementary Figure 12. 11
Supplementary Figure 13. 11
Supplementary Figure 14. 12
Supplementary Figure 15. 13
Supplementary Figure 16. 14
Supplementary Figure 17. 15
Supplementary Figure 18. 16
Supplementary Figure 19. 17
Supplementary Figure 20. 18
Supplementary Figure 21. 19
Supplementary Figure 22. 20
Supplementary Figure 23. 21
Supplementary Figure 24. 22
Supplementary Figure 25. 23
Supplementary Tables 25
Supplementary Table 1 25
Supplementary Table 2 25
Supplementary Table 3 26
Supplementary Table 4 27
Supplementary Table 5 29
Supplementary Table 6 30
Supplementary Table 7 34
Supplementary References 36

Supplementary Figures

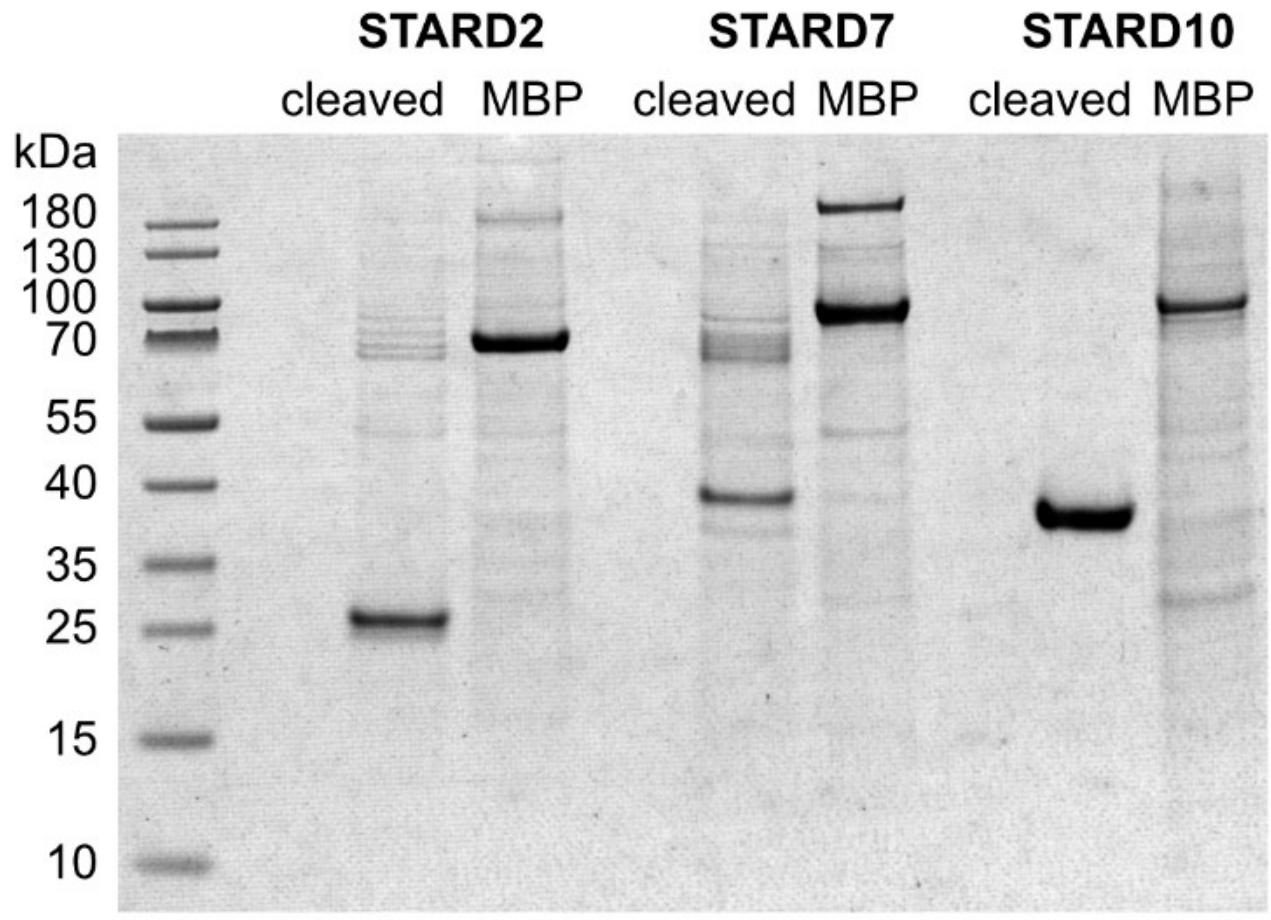

Supplementary Figure 1. SDS-PAGE of STARD2, STARD7(76–370), and STARD10 expressed in *E. coli*, before and after cleavage of the N-terminal maltose-binding protein (MBP) tag. Protein purification was performed using a single biological replicate (n = 1). The uncropped gel is provided in the Source Data file.

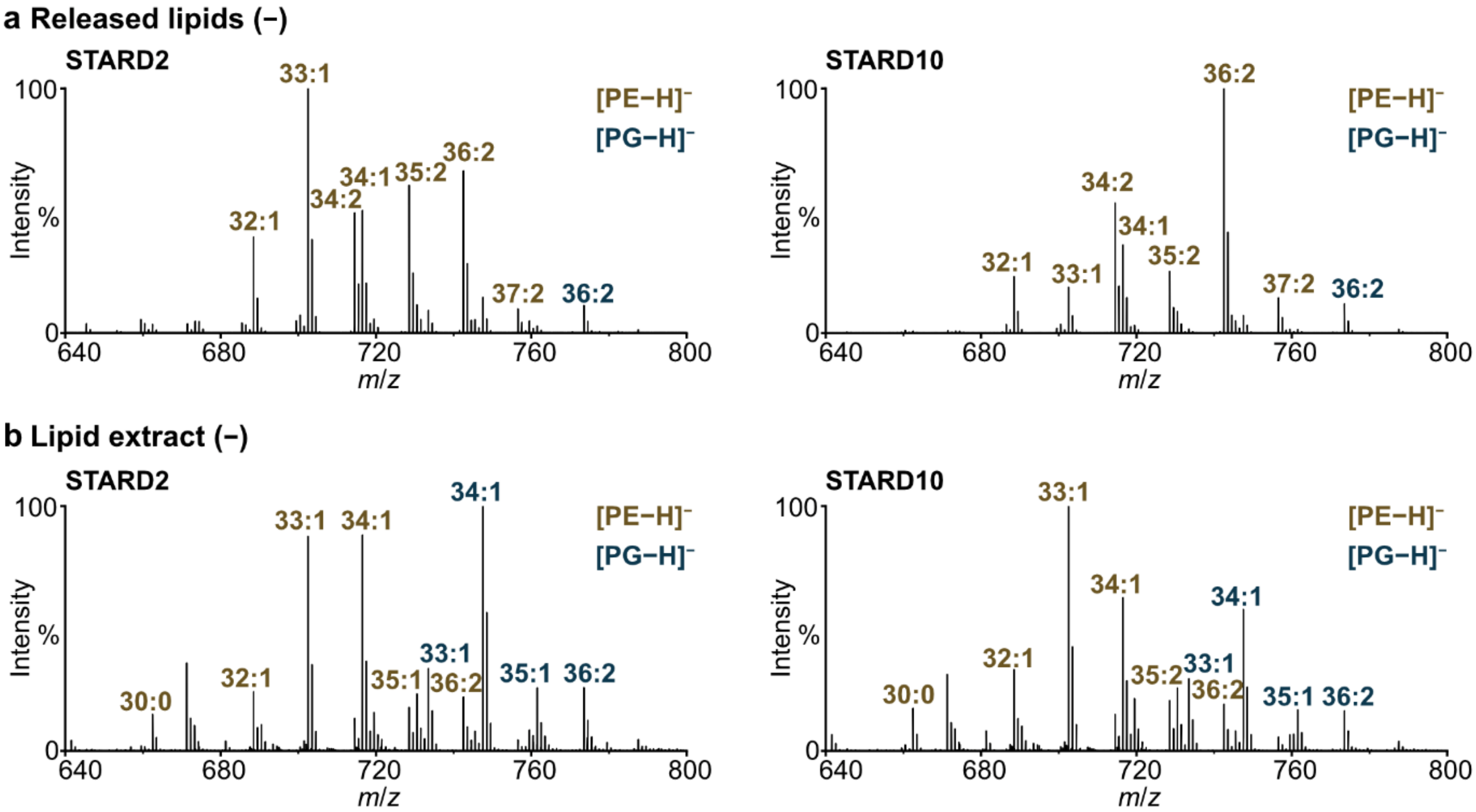

Supplementary Figure 2. Lipids copurified with STARD2 and STARD10 upon expression in *E. coli*. **a.** MS^2 spectra showing phospholipids released from purified STARD2 and STARD10 in negative ion mode. The lipids are assigned to bacterial PE (beige) and PG (blue) with different acyl chain compositions. **b.** Direct-infusion mass spectra of lipids extracted from bacteria overexpressing STARD2 or STARD10, recorded in negative ion mode. Spectra were recorded in technical triplicate, and representative spectra are shown.

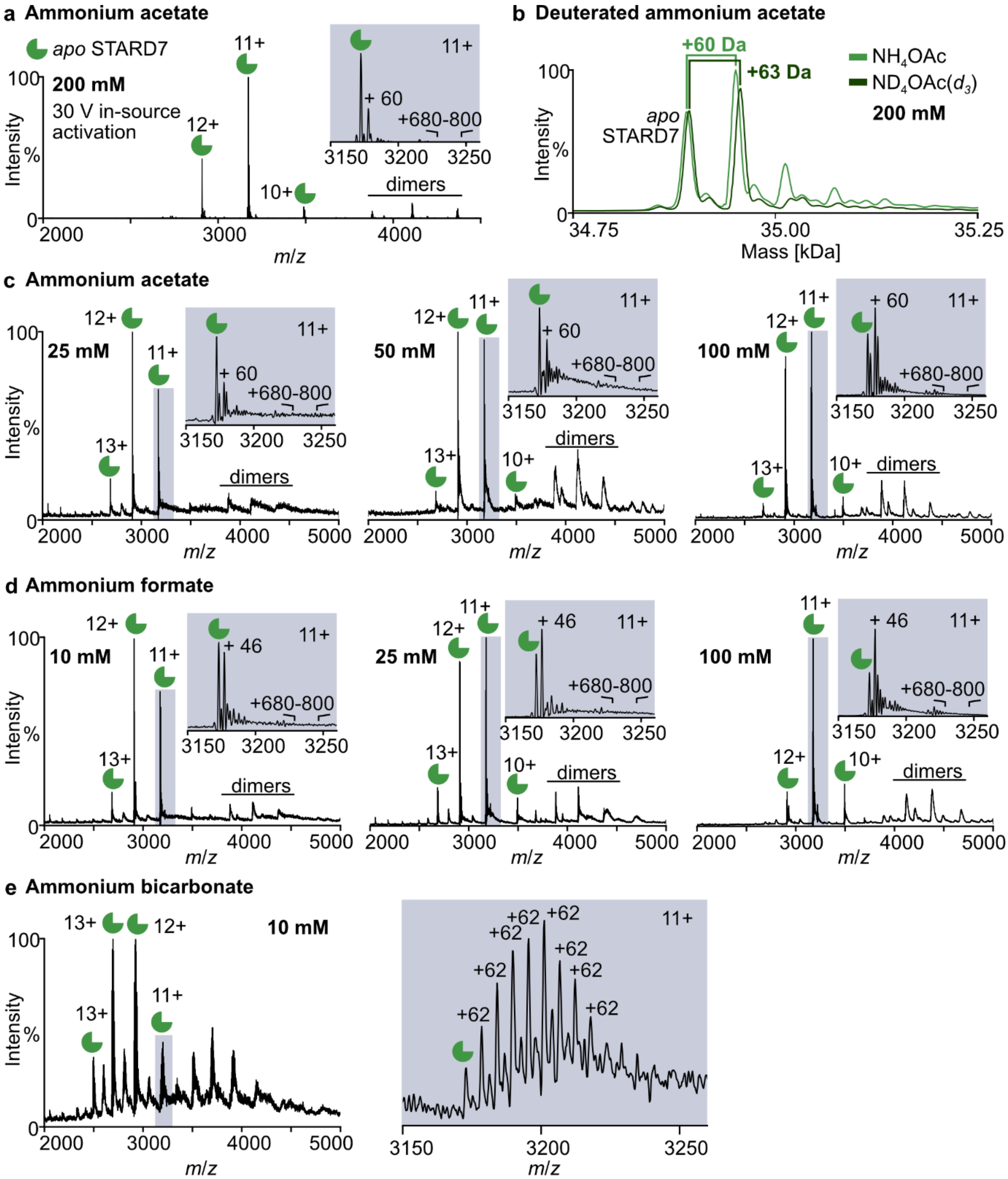

Supplementary Figure 3. Native mass spectra of STARD7(76–370; expressed in *E. coli*) obtained in different buffers. **a.** Native mass spectrum of STARD7 in 200 mM ammonium acetate recorded with in-source activation (30 V). In-source activation leads to a reduction of the +60 Da peak, suggesting non-covalent binding. **b.** Deconvoluted native mass spectra of STARD7 obtained in 200 mM ammonium acetate or ammonium acetate-d7. The adduct peak shifts by 3 Da in ammonium acetate-d7. **c.** Native mass spectra of STARD7 obtained in ammonium acetate buffer at varying concentrations (25–100 mM). The relative intensity of the adduct peak is concentration-dependent. **d.** Native mass spectra of STARD7 obtained using ammonium formate buffer at varying concentrations (10–100 mM). Formate forms 1:1 complexes with STARD7 similar to acetate (+46 Da). **e.** Native mass spectrum of STARD7 in

ammonium bicarbonate (10 mM). Bicarbonate forms multiple non-specific adducts with STARD7. Phospholipid binding (expected range +680–800 Da) is not observed under any condition. One spectrum was recorded per condition.

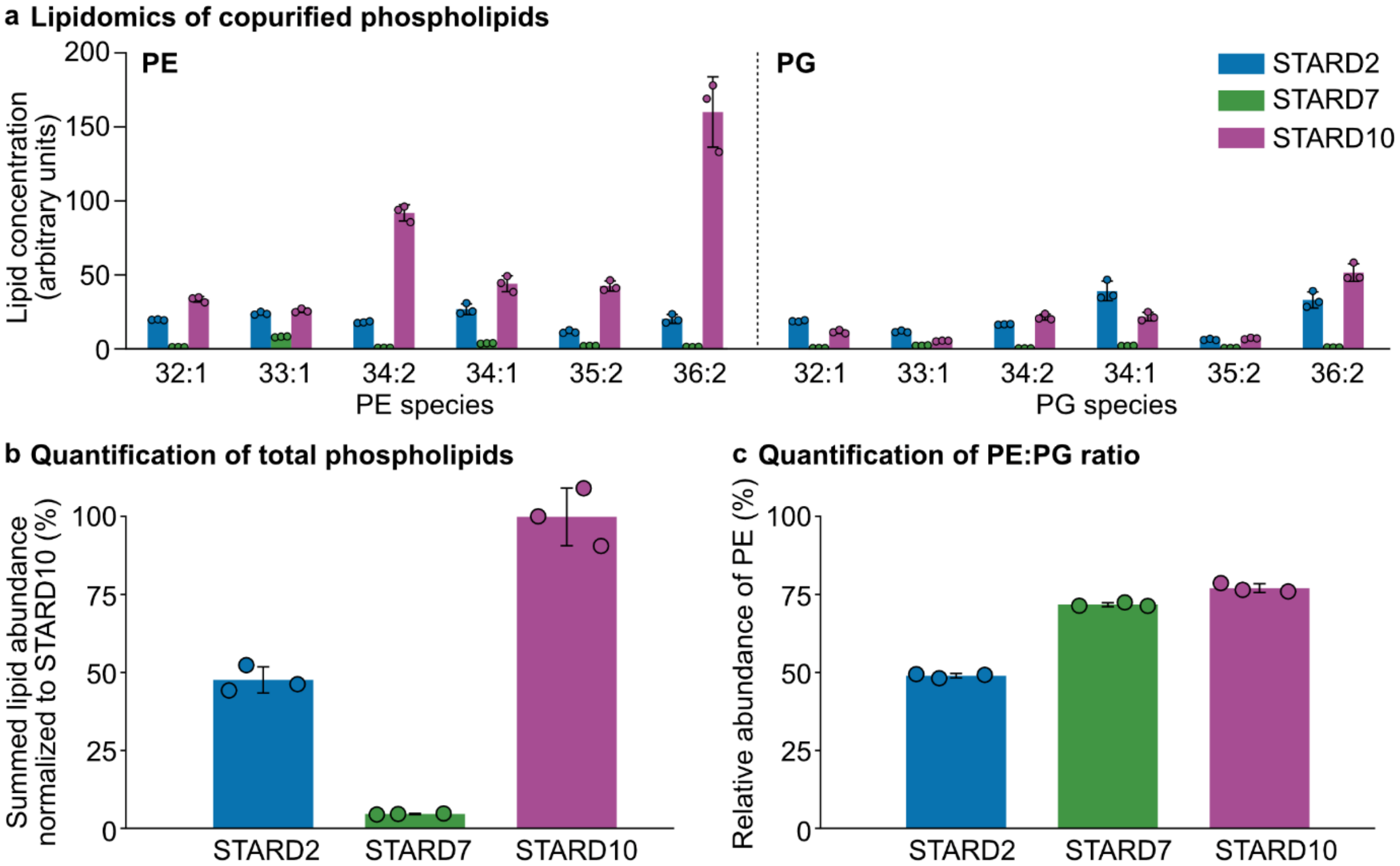

Supplementary Figure 4. Quantification of phospholipids copurified with STARD2 (blue), STARD7(76–370) (green), and STARD10 (purple) expressed in *E. coli* using LC-MS/MS lipidomics. **a.** Quantification of individual PE and PG species relative to PE 33:1-d7 and PG 33:1-d7 standards. **b.** Quantification of total copurified phospholipids (summed PE and PG) normalized to the lipid abundance copurified with STARD10. **c.** Relative abundance of PE (% of total PE+PG) copurified with STARD2, STARD7(76–370), and STARD10. Data points represent results from three technical replicates. Bar charts represent the mean ± standard deviation. Source data are provided as a Source Data file.

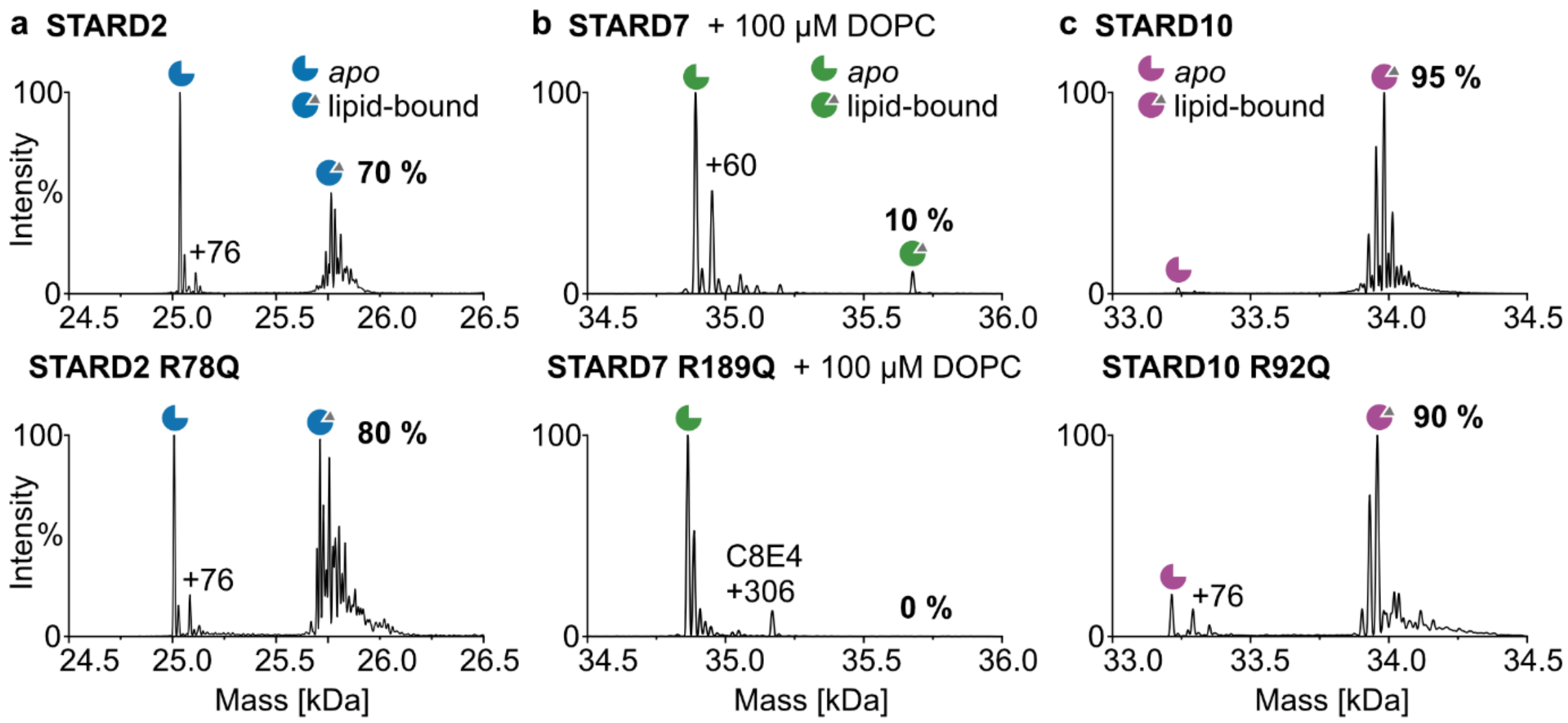

Supplementary Figure 5. Deconvoluted native mass spectra showing lipid binding to STARD2 R78Q, STARD7(76–370) R189Q, and STARD10 R92Q arginine-to-glutamine mutants. The point mutation does not reduce the ratio of phospholipids copurifying with STARD2 (blue) (**a**) and STARD10 (purple) (**c**) but abolishes lipid binding to STARD7(76–370) (green) (**b**). The STARD7(76–370) R189Q mutant neither binds acetate nor phospholipids, when incubated with 100 µM DOPC in ammonium acetate + 2×CMC C8E4. Percentages were obtained by integrating peak areas corresponding to *apo* and lipid-bound proteins and were rounded to ±5 %, based on a single measurement (n = 1). Source data are provided as a Source Data file.

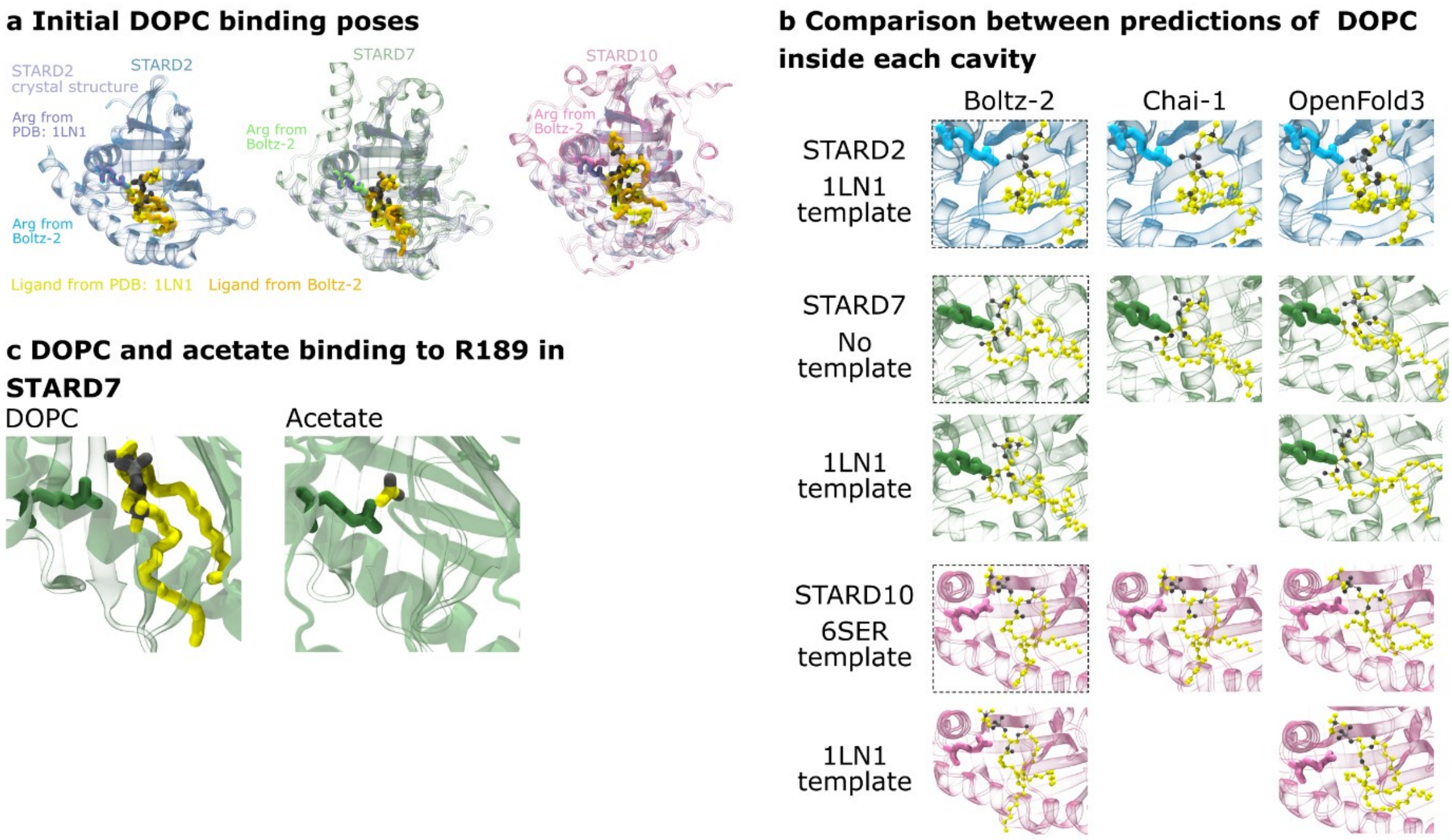

Supplementary Figure 6. Predicted models of DOPC-bound STARD2 (blue), STARD7 (green) and STARD10 (purple). **a.** Binding poses of DOPC in Boltz-2-predicted models with reference to the crystal structure of lipid-bound STARD2 (PDB-ID: 1LN1). Lipid ligands and conserved arginine residues are highlighted. **b.** DOPC poses relative to the conserved arginine from different prediction tools for each protein with and without using a template to guide the predictions. The phosphate atoms are shown in light grey, the choline N atoms are the topmost dark grey spheres in each snapshot. The predictions selected for the simulations are highlighted in dashed boxes. **c.** Boltz-2 predictions of STARD7 with bound DOPC or acetate show interactions with the conserved Arg189.

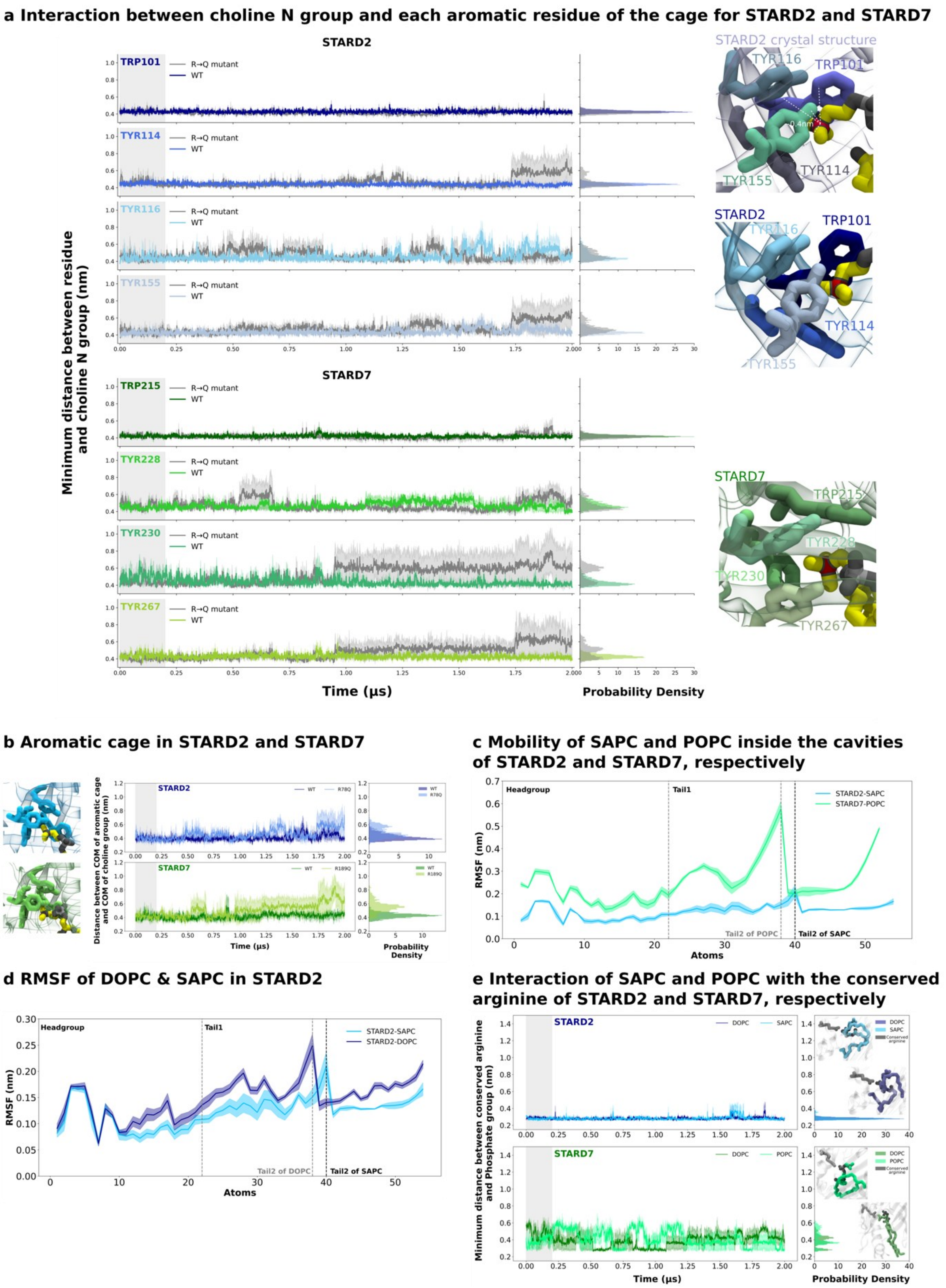

Supplementary Figure 7. Molecular dynamics simulations of lipid-bound STARD2 (blue) and STARD7 (green). **a.** Right: Boltz-2 predictions of the DOPC headgroup (choline N group highlighted in red) interacting with the aromatic residues from the crystal structure of STARD2 (PDB-ID: 1LN1) and in each protein. Left: Minimum distance between the choline N group of DOPC and each aromatic residue of the conserved cages in STARD2 and STARD7 in WT

(dark colored traces) and the R→Q mutant (dark grey traces). The shaded region around the solid line is the standard error of mean. The first 0.2 μs of simulation, shaded in gray, were excluded in the probability density calculation. **b.** Left: Boltz-2 predictions of DOPC interacting with the aromatic cage of STARD2 and STARD7. Right: COM distances between the choline group of DOPC and the aromatic cage in the wild type proteins (dark traces) or in the arginine-to-glutamine mutants (light traces). The shaded region around the solid line is the standard error of mean. The first 0.2 μs of simulation, shaded in gray, were excluded in the probability density calculation. **c.** Mobility of SAPC and POPC inside the cavities of STARD2 and STARD7, respectively, as shown by the RMSF of the lipids in AA-MD. The shaded region is the standard error of mean. **d.** Mobility of SAPC and DOPC inside the cavity of STARD2 as shown by the RMSF of the lipids in AA-MD. The shaded region is the standard error of mean. **e.** Minimum distance between the phosphate group of DOPC (dark traces) or the phosphate group of SAPC or POPC (neon traces) and the respective conserved arginine residue. Insets show the lipids inside the binding pockets at the end of the simulation (arginine is shown in silver, DOPC is shown in dark green/blue, SAPC is shown in neon blue, POPC is shown in neon green). The shaded region around the solid line is the standard error of mean. The first 0.2 μs of simulation, shaded in gray, were excluded in the probability density calculation and the RMSF calculation. Source data are provided as a Source Data file.

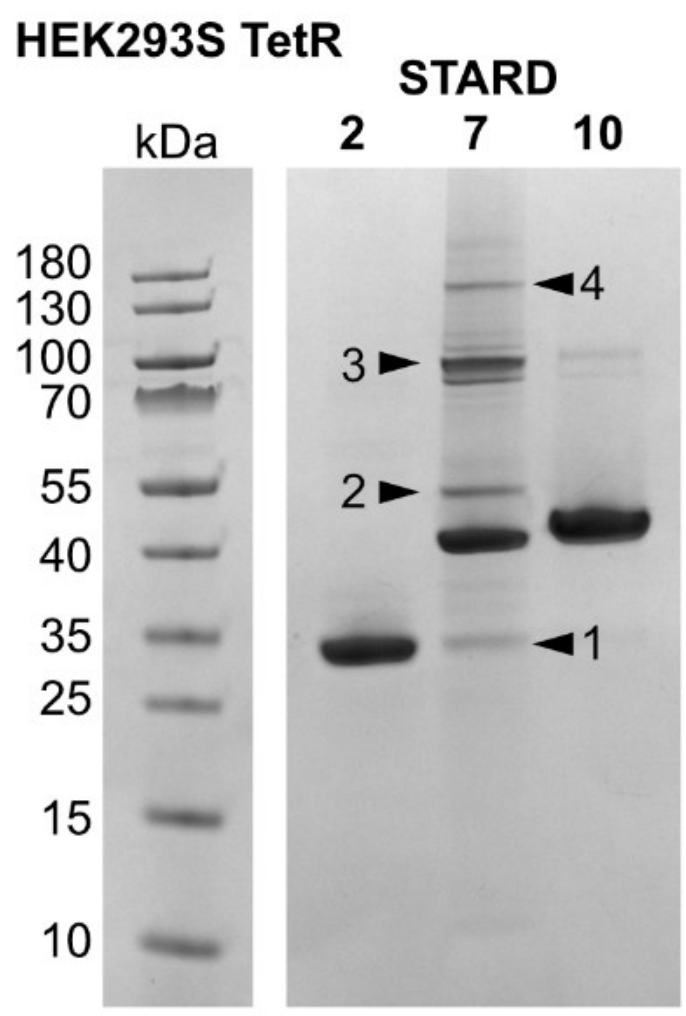

Supplementary Figure 8. SDS-PAGE of STARD2, STARD7, and STARD10 expressed in HEK293S TetR cells after elution from anti-FLAG beads. All major and annotated minor bands were analyzed by LC–MS/MS proteomics following in-gel digestion. The main band in the STARD7 lane corresponds to the monomer with cleaved mitochondrial localization signal. Band 1 could not be unambiguously assigned. Bands 2–4 yielded STARD7 as the primary hit, consistent with the presence of STARD7 monomer retaining the mitochondrial localization signal (band 2), STARD7 dimer (band 3), and STARD7 trimer (band 4). Uncropped gels from three biological replicates (*n* = 3) are provided in the Source Data file.

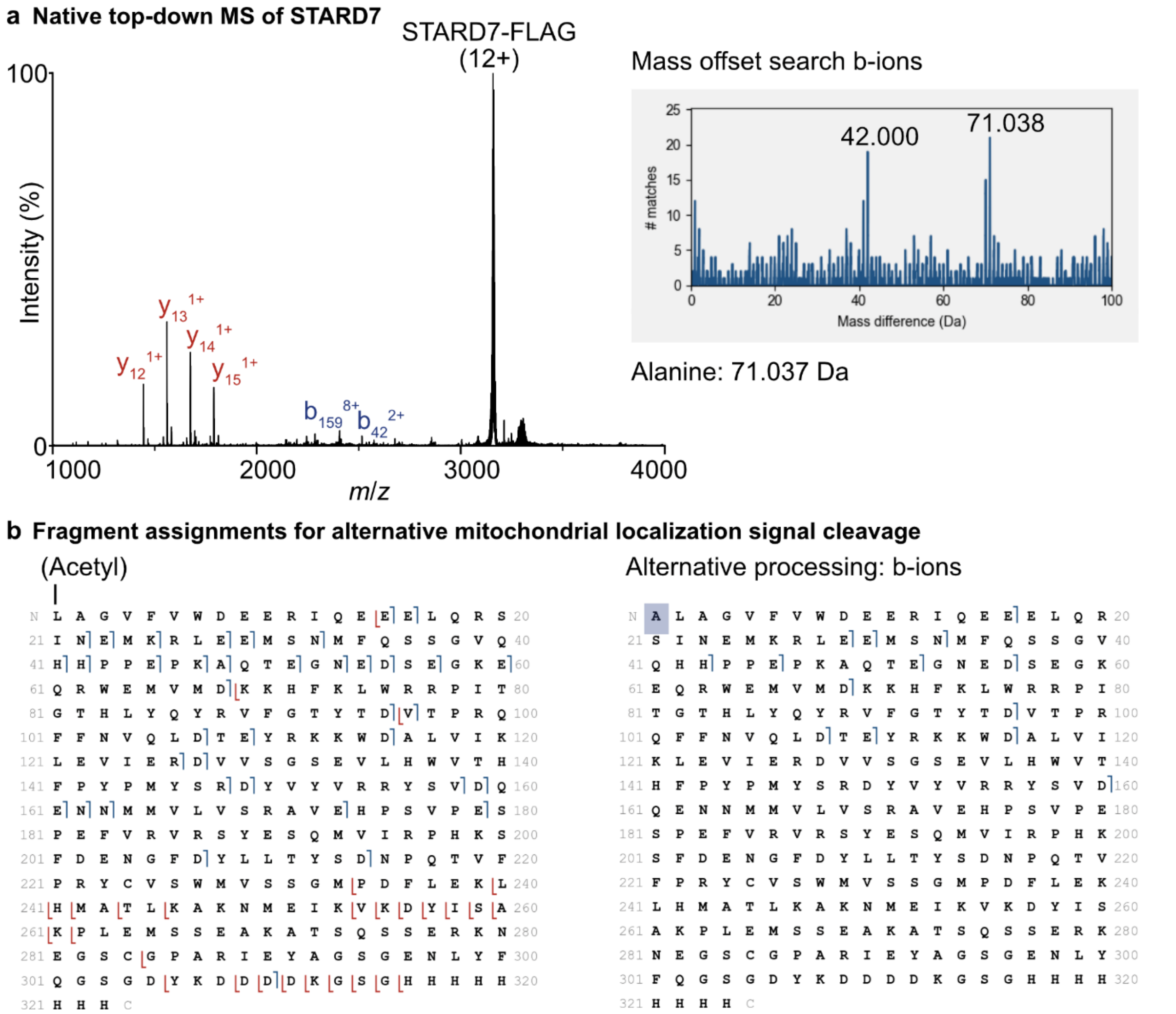

Supplementary Figure 9. Native top-down MS of STARD7 (12+ charge state, HCD 100V). **a.** The majority of sequence ions can be assigned to STARD7 cleaved between A78 and L79. A mass offset search of unassigned fragment ions showed that another set of fragment ions is shifted by the mass of an additional alanine (Δm = 71 Da), suggesting alternative cleavage between A77 and A78. **b.** Fragment maps for both protein isoforms are shown (blue: b-type ions, red: y-type ions). Because y-type ions are identical for both isoforms, only b-type ions revealing the alternative cleavage site are shown for the minor isoform.

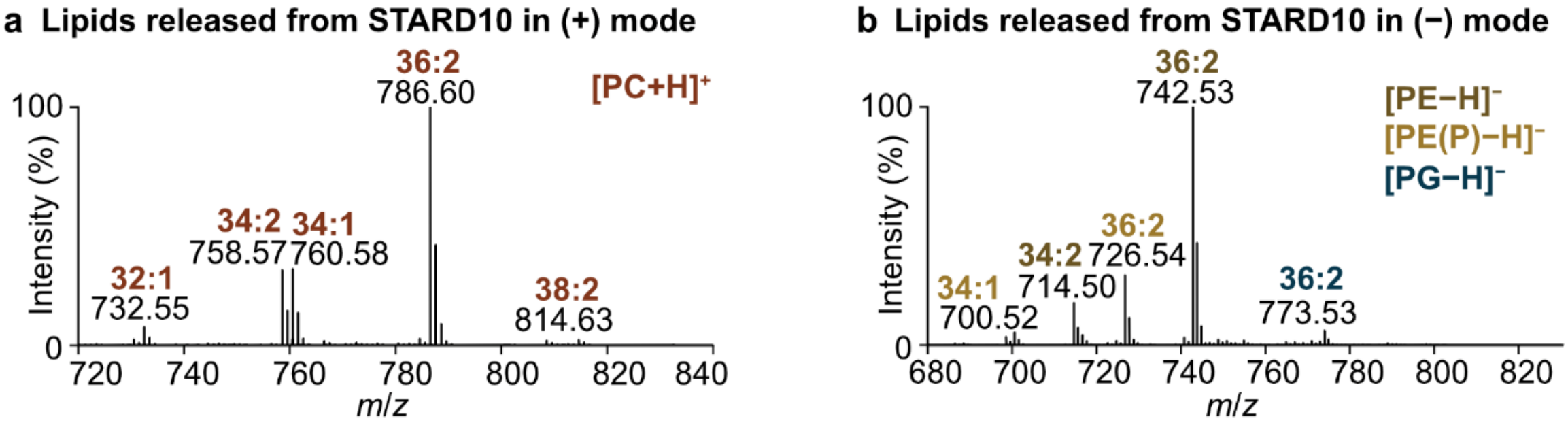

Supplementary Figure 10. MS^2 spectra comparing lipids released from STARD10 expressed in HEK293S cells in positive and negative ion modes. **a.** In positive ion mode, PC is the only lipid class detected and is observed as protonated ions (orange). **b.** In negative ion mode, deprotonated PE (beige), plasmenyl-PE (yellow), and PG (blue) are detected. All lipid classes show similar acyl chain profiles. MS^2 spectra were recorded in biological triplicate, and representative spectra are shown.

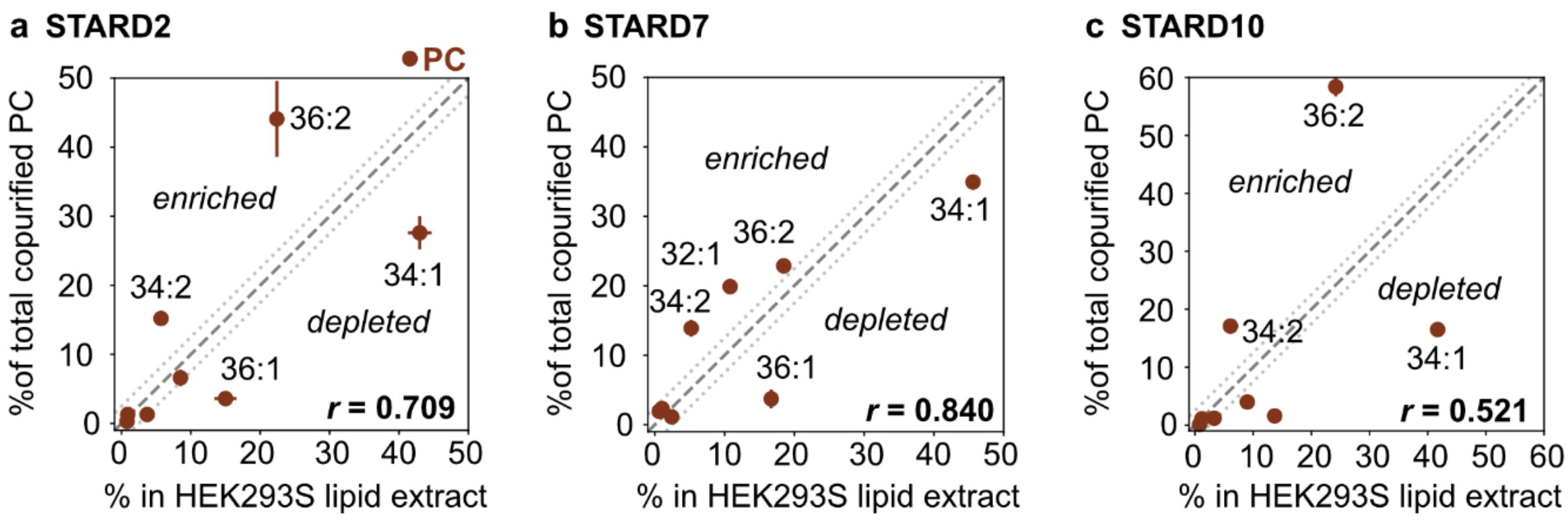

Supplementary Figure 11. Correlation plots comparing the relative abundance of individual PC species (orange) copurified with STARD2 (**a**), STARD7 (**b**), or STARD10 (**c**) to their corresponding relative abundance in whole-cell lipid extracts from HEK293S cells. Data points represent the mean of three biological replicates, and error bars indicate the standard deviation. Correlation coefficients were calculated using Pearson's *r*. The dotted light gray lines indicate ±2.5% deviation from the diagonal. Source data are provided as a Source Data file.

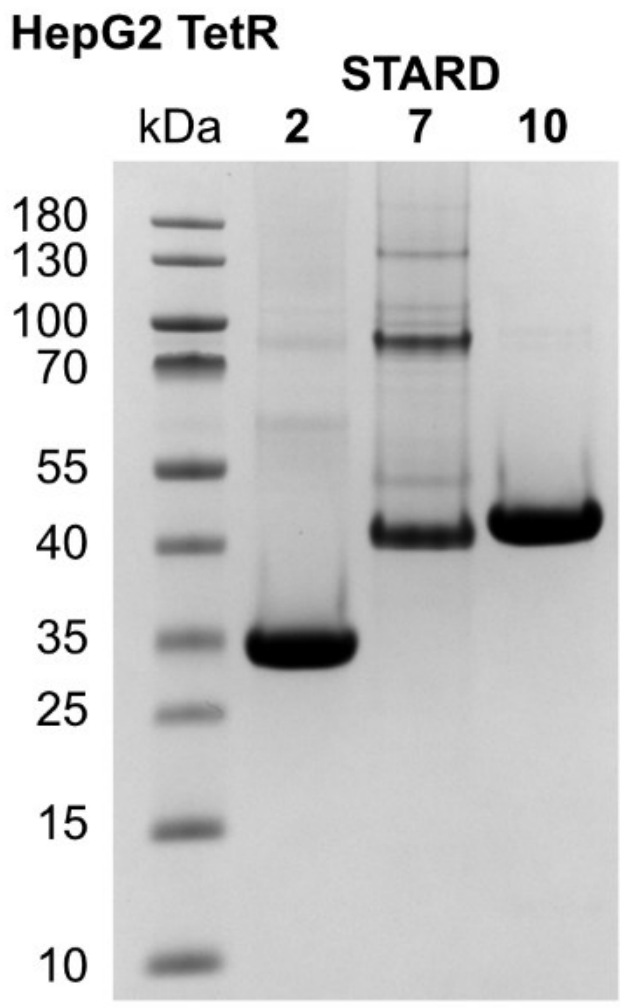

Supplementary Figure 12. SDS-PAGE of STARD2, STARD7, and STARD10 expressed in HepG2 TetR cells after elution from anti-FLAG beads. Uncropped gels from three biological replicates (n = 3) are provided in the Source Data file.

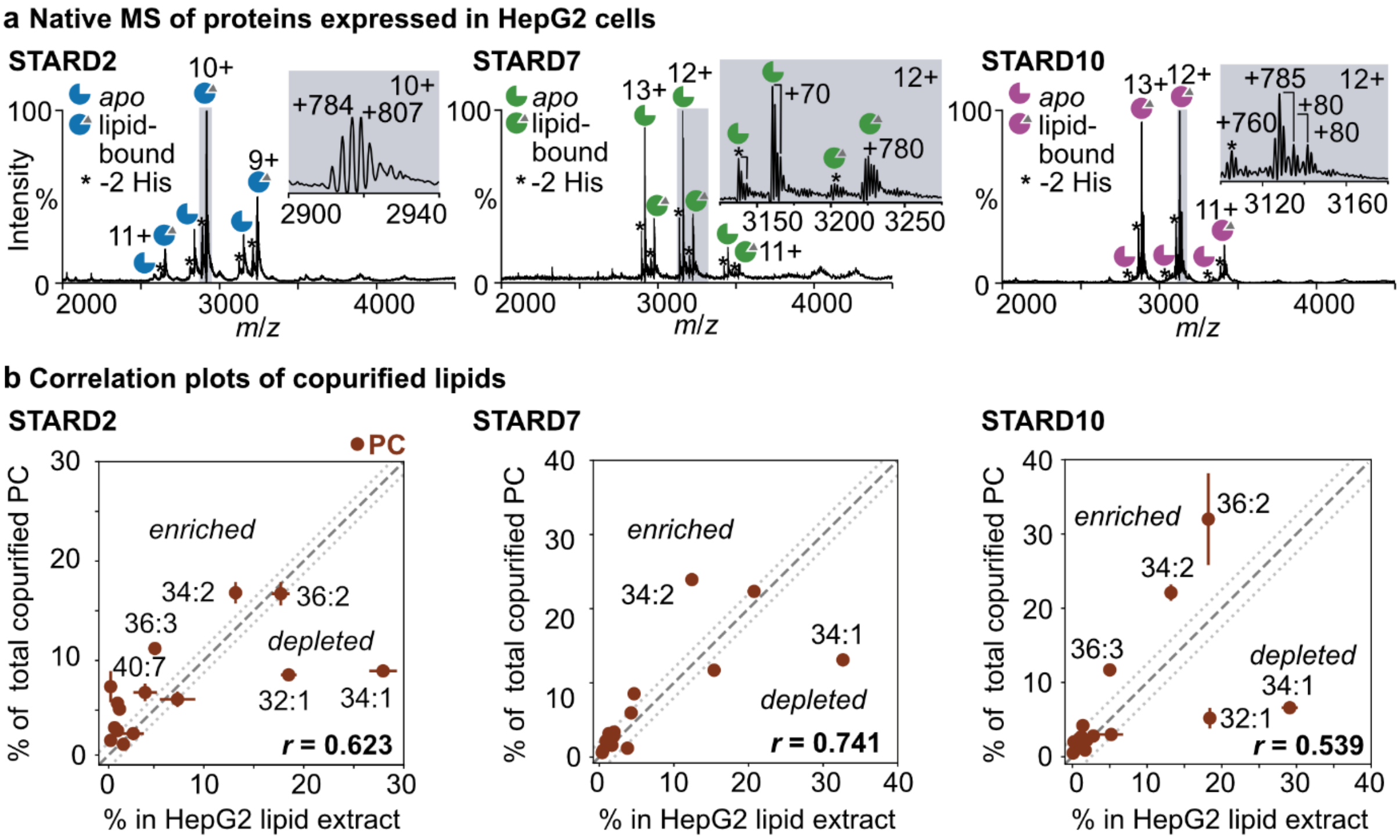

Supplementary Figure 13. Characterization of STARD2 (blue), STARD7 (green), and STARD10 (purple) expressed in HepG2 cells. **a.** Native mass spectra of STARD2, STARD7, and STARD10 following expression in HepG2 cells. All spectra reveal partial C-terminal truncation corresponding to the loss of two histidine residues (*). Native mass spectra were recorded in biological triplicate, and representative spectra are shown. **b.** Correlation plots comparing the relative abundance of individual PC species copurified with STARD2, STARD7, and STARD10 to their corresponding relative abundance in whole-cell lipid extracts from HepG2 cells. Data points represent the mean of three biological replicates, and error bars indicate the standard deviation. Correlation coefficients were calculated using Pearson's r. The dotted light gray lines indicate ±2.5% deviation from the diagonal. Source data are provided as a Source Data file.

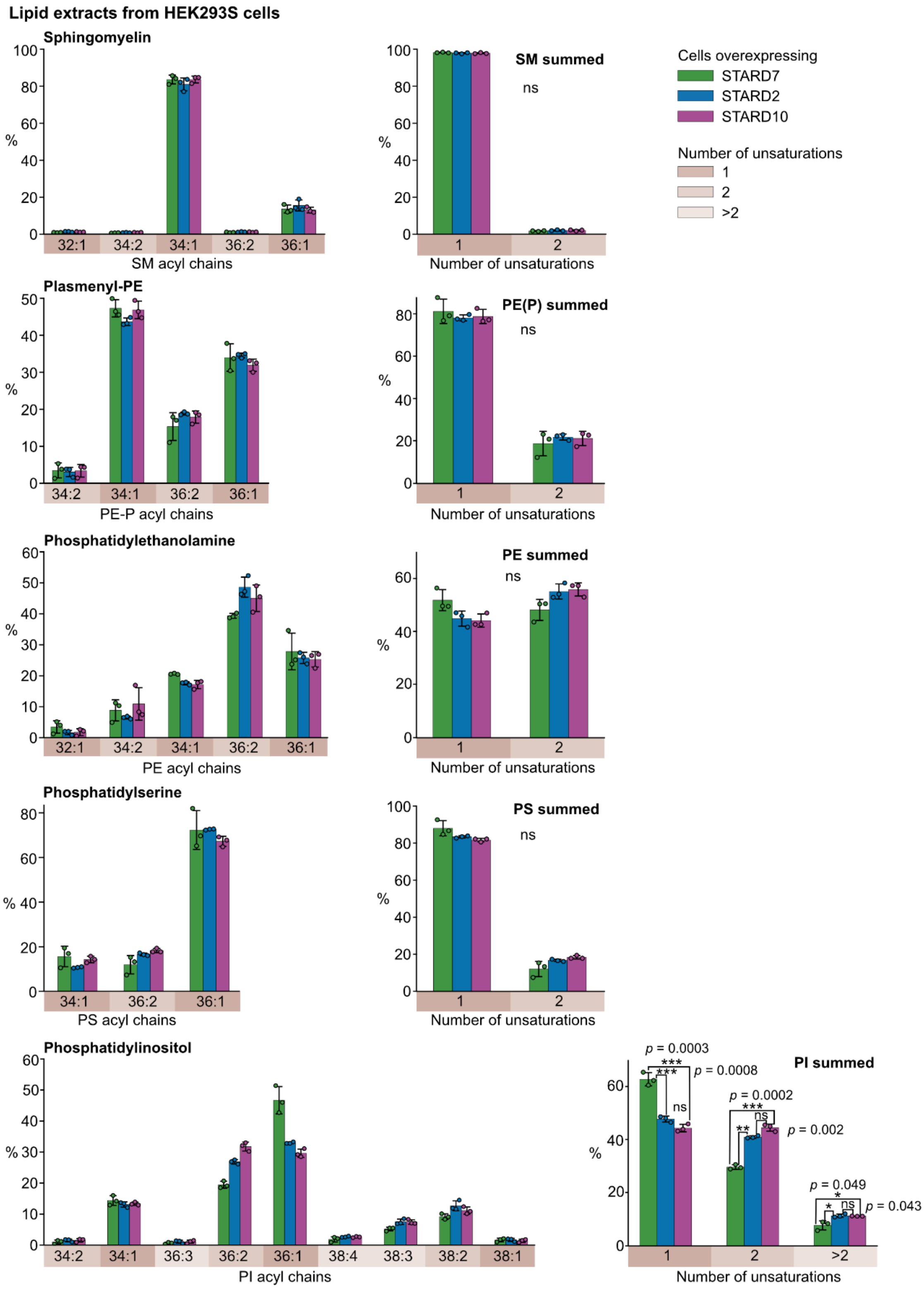

Supplementary Figure 14. Acyl chain profiles of different phospholipid classes in HEK293S cells overexpressing STARD2, STARD7, or STARD10. PE species follow a similar trend as PC but show no statistical significance. The acyl chain profiles of PI are significantly affected by overexpression of the LTPs. Bars represent the mean of three biological replicates, and error bars represent the standard deviation. To assess significance, data were centered log-ratio (CLR) transformed to account for compositional constraints, and statistical significance was determined using one-way ANOVA, with pairwise comparisons by Tukey's HSD.

Statistical significance is indicated based on adjusted p-values: * p < 0.05, ** p < 0.01, *** p < 0.001; ns = not significant. Source data are provided as a Source Data file.

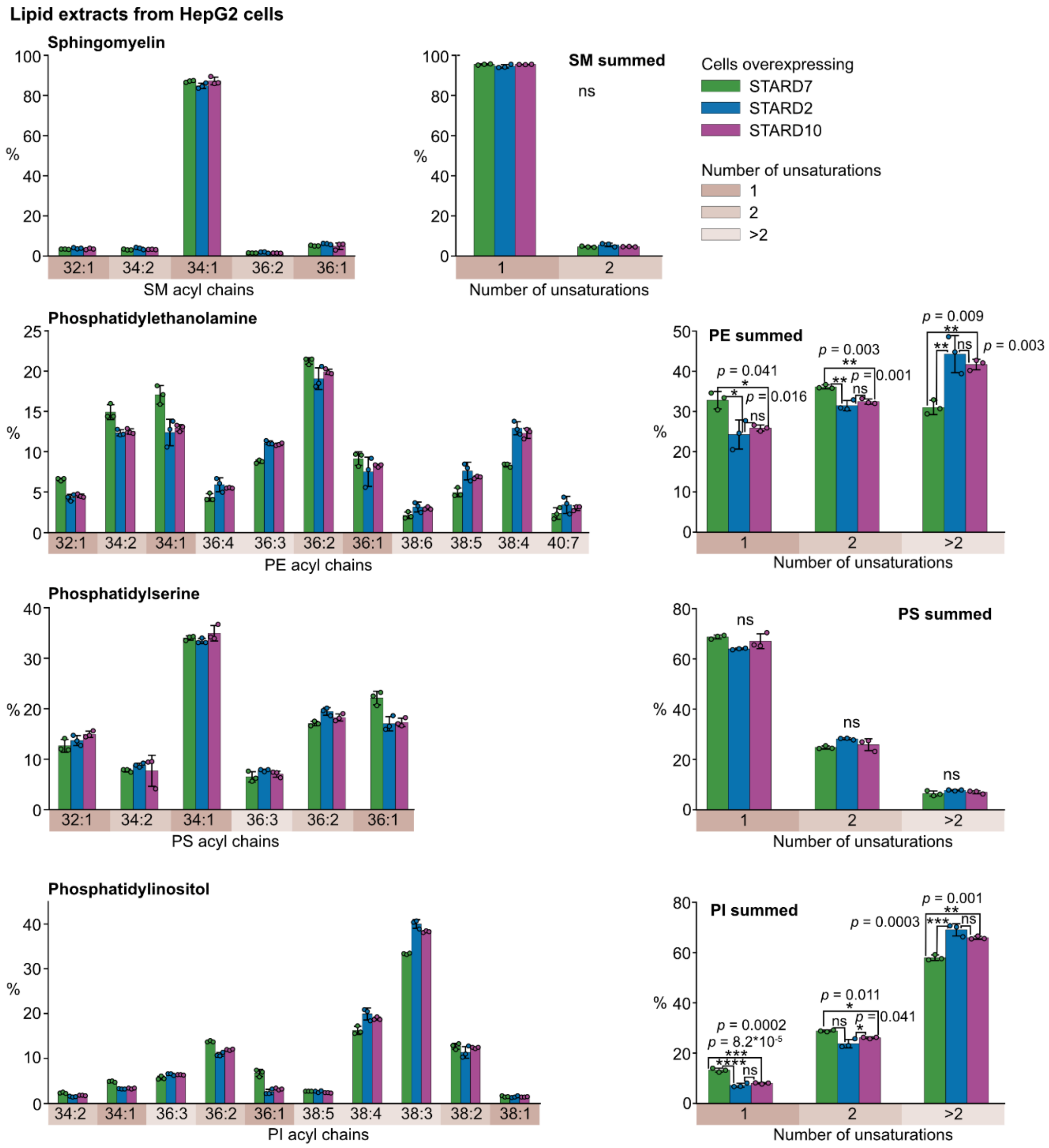

Supplementary Figure 15. Acyl chain profiles of different phospholipid classes in HepG2 cells overexpressing STARD2, STARD7, or STARD10. PE and PI acyl chains change following a similar trend as PC. Bars represent the mean of three biological replicates, and error bars represent the standard deviation. To assess significance, data were centered log-ratio (CLR) transformed to account for compositional constraints, and statistical significance was determined using one-way ANOVA, with pairwise comparisons by Tukey's HSD. Statistical significance is indicated based on adjusted p-values: * p < 0.05, ** p < 0.01, *** p < 0.001, **** p < 0.0001; ns = not significant. Source data are provided as a Source Data file.

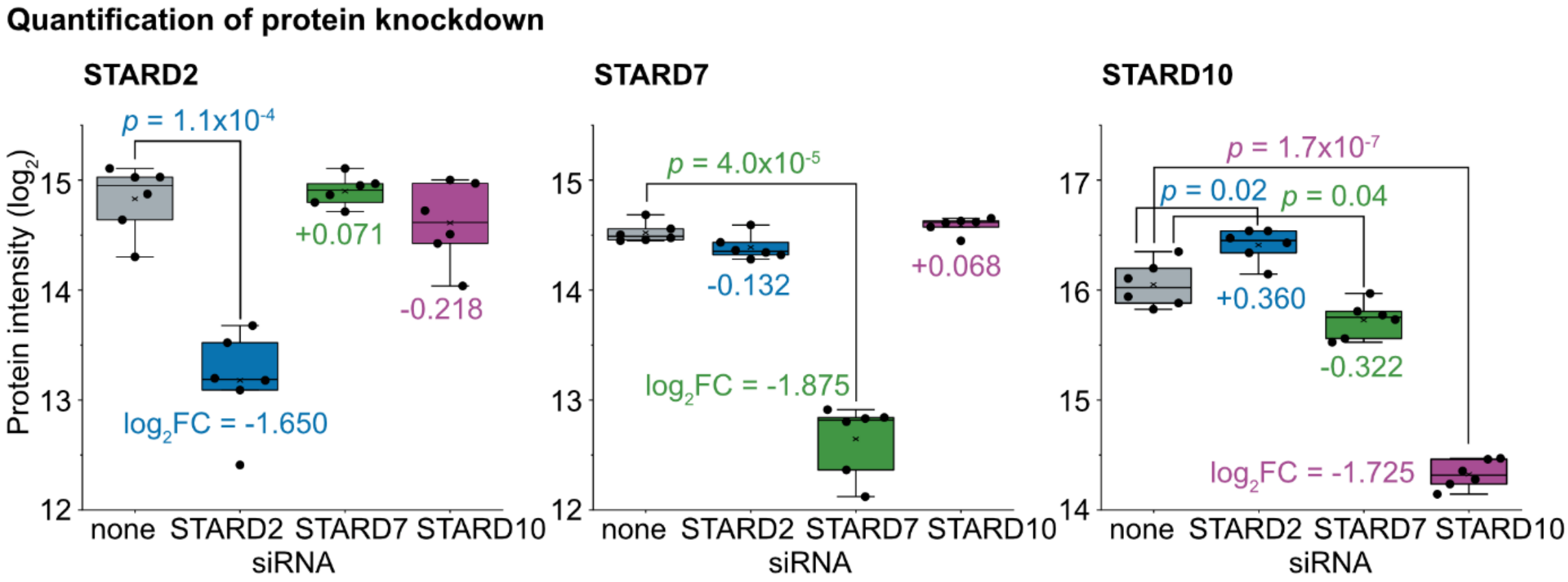

Supplementary Figure 16. Changes in the cellular proteome upon siRNA-mediated knockdown of STARD2, STARD7, or STARD10 quantified by MS-based proteomics. p-values were obtained from pairwise two-sided Welch's t-tests comparing the control to each of the three siRNA knockdown conditions, with Bonferroni correction applied for multiple testing. Only statistically significant p-values are shown. Log_2-fold changes are reported for all conditions relative to the control sample. Source data are provided as a Source Data file.

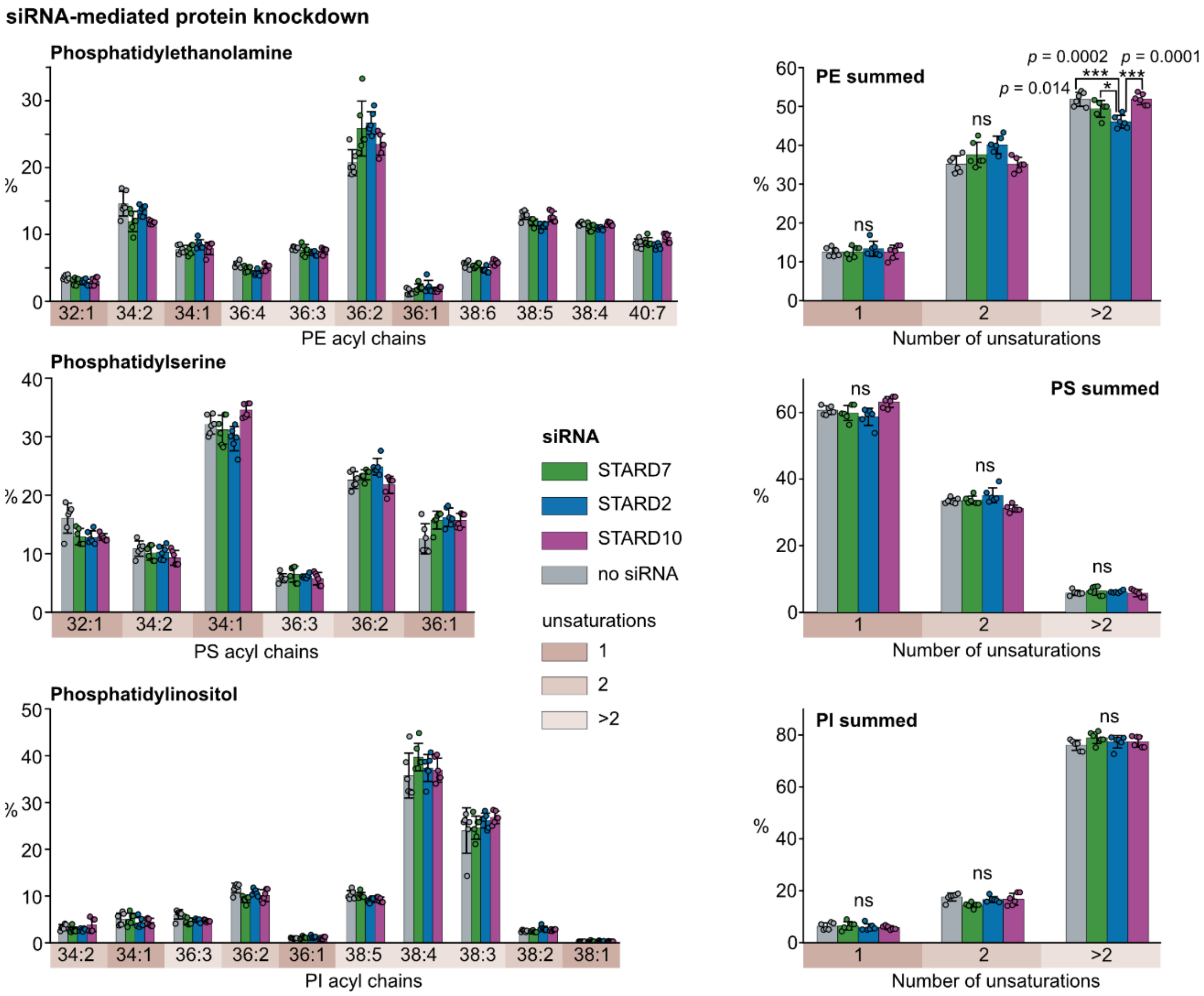

Supplementary Figure 17. Acyl chain profiles of different phospholipid classes in HepG2 cells following 72 h siRNA-mediated knockdown of STARD2, STARD7, or STARD10. Polyunsaturated PE is reduced in STARD2 knockdown cells Bars represent the mean of three biological replicates, and error bars represent the standard deviation. To assess significance, data were centered log-ratio (CLR) transformed to account for compositional constraints, and statistical significance was determined using one-way ANOVA, with pairwise comparisons by Tukey's HSD. Statistical significance is indicated based on adjusted p-values: * $p < 0.05$, ** $p < 0.01$, *** $p < 0.001$; ns = not significant. Source data are provided as a Source Data file.

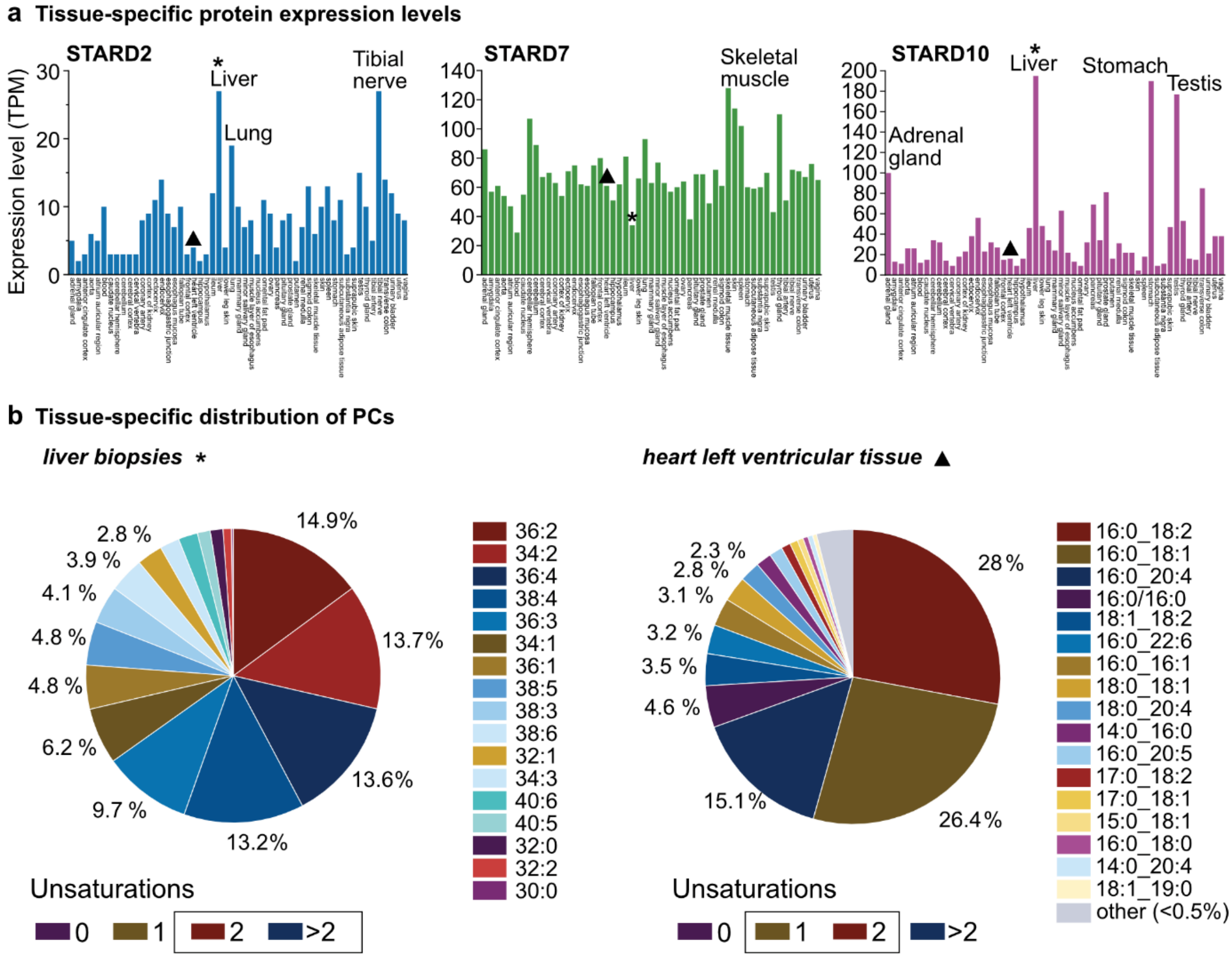

Supplementary Figure 18. Correlation between expression levels of STARD phospholipid transfer proteins and phosphatidylcholines in different tissues. **a.** Expression levels of STARD2, STARD7, and STARD10 across human tissues based on transcriptomics data (TPM = transcripts per million). Data were exported from the EMBL-EBI Expression Atlas (http://www.ebi.ac.uk/gxa).[1] **b.** Variation of the PC acyl chain composition in different tissues. Lipidomics analyses of human liver[2] and heart left ventricular tissue[3] are exemplarily shown. The liver samples mainly contain doubly and polyunsaturated PC lipids that are preferred ligands of STARD10 and STARD2, respectively. Though individual acyl chains were not determined, we infer from lipidomics data on HepG2 cells that doubly unsaturated PC 36:2 and 34:2 carry one unsaturation in each chain. Heart tissue contains a larger fraction of monounsaturated PC, whereas PC species with one unsaturation in each chain are not detected. Source data are provided as a Source Data file.

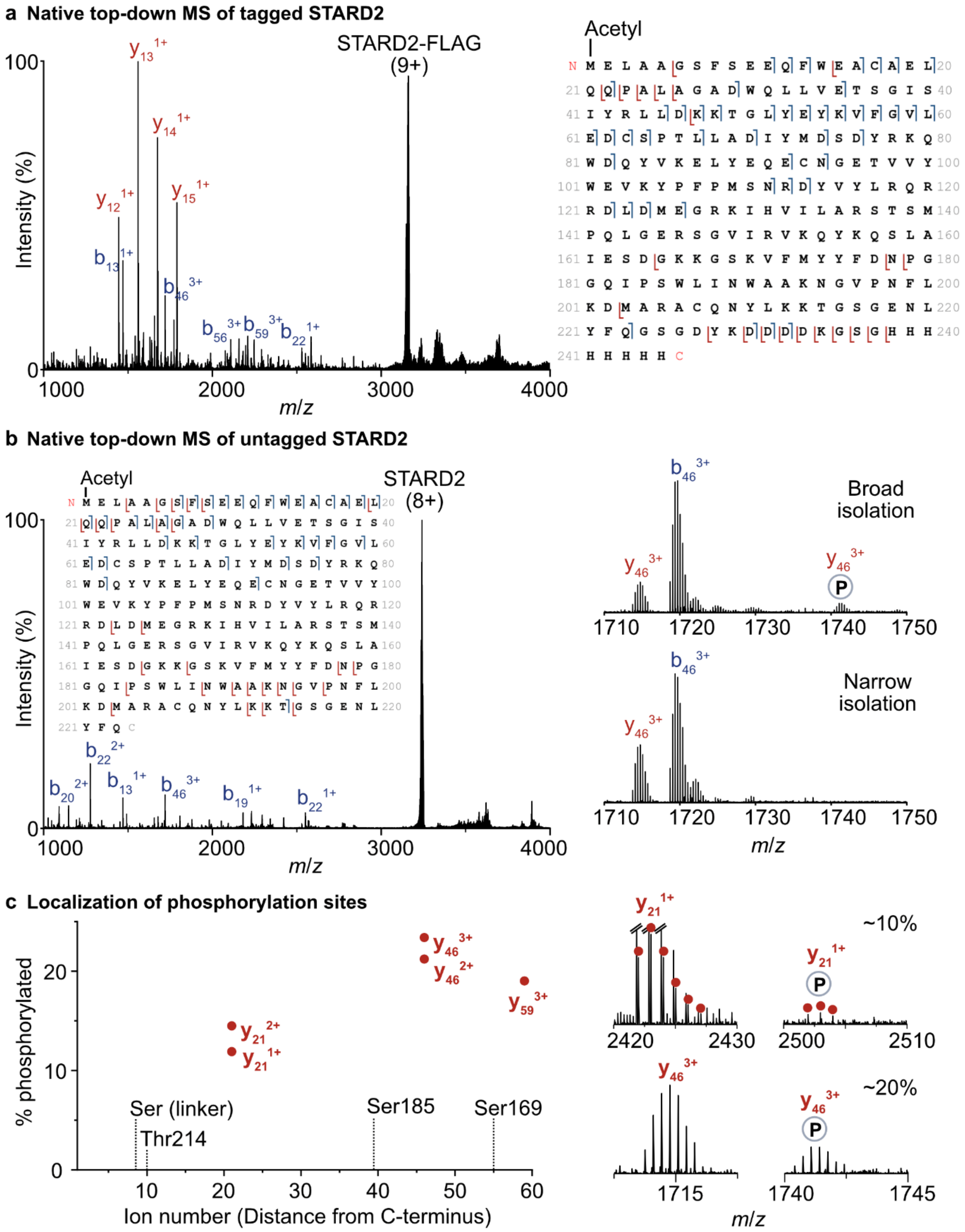

Supplementary Figure 19. Native top-down MS of STARD2. **a.** Fragmentation of FLAG-tagged STARD2 (9+ charge state, HCD 80V) did not yield phosphorylated peptides. **b.** After cleavage of the FLAG tag, C-terminal fragments with phosphate modification were obtained (8+ charge state, HCD 70 V). Phosphorylated fragments were observed when the phosphorylated precursor ion was included in the isolation window (*m*/*z* 3245 ± 10), and were absent when the *apo* protein was isolated alone (*m*/*z* 3240 ± 5). **c.** Two phosphorylation sites with ca. 10 % occupancy each were detected: Thr214 or Ser in the GSG linker (exact position cannot be determined), and Ser185. Relative intensities of phosphorylated fragments are

exemplarily shown for y21 and y46 ions. All b-type ions carry an acetyl modification, supporting N-acetylation of the protein. Source data are provided as a Source Data file.

STARD2

MELAAGSFSEEQFWEACAELQQPALAGADWQLLVETSGISIYRLLDKKTGLYEYKVFGVLEDCSPTLLADIYMDSDYRKQWDQY

137
VKELYEQECNGETVVYWEVKYPFPMSNRDYVYLRQRRDLDMEGRKIHVILARSTSMPQLGERSGVIRVKQYKQSLAIESDGKKG

171 **185** 213
SKVFMYYFDNPGGQIP**S**WLINWAAKNGVPNFLKDMARACQNYLKKTGSGENLYFQGSGDYKDDDDKGSGHHHHHHHH

Linker TEV protease cleavage site FLAG tag His8 tag

Supplementary Figure 20. Proteomics analysis of STARD2 after in-gel digestion. The sequence map shows detected phosphorylated peptides (underlined). All peptides are 2–3 % phosphorylated. Ser185 is highlighted. The measurement was performed once.

a **Native top-down MS of tagged STARD10**

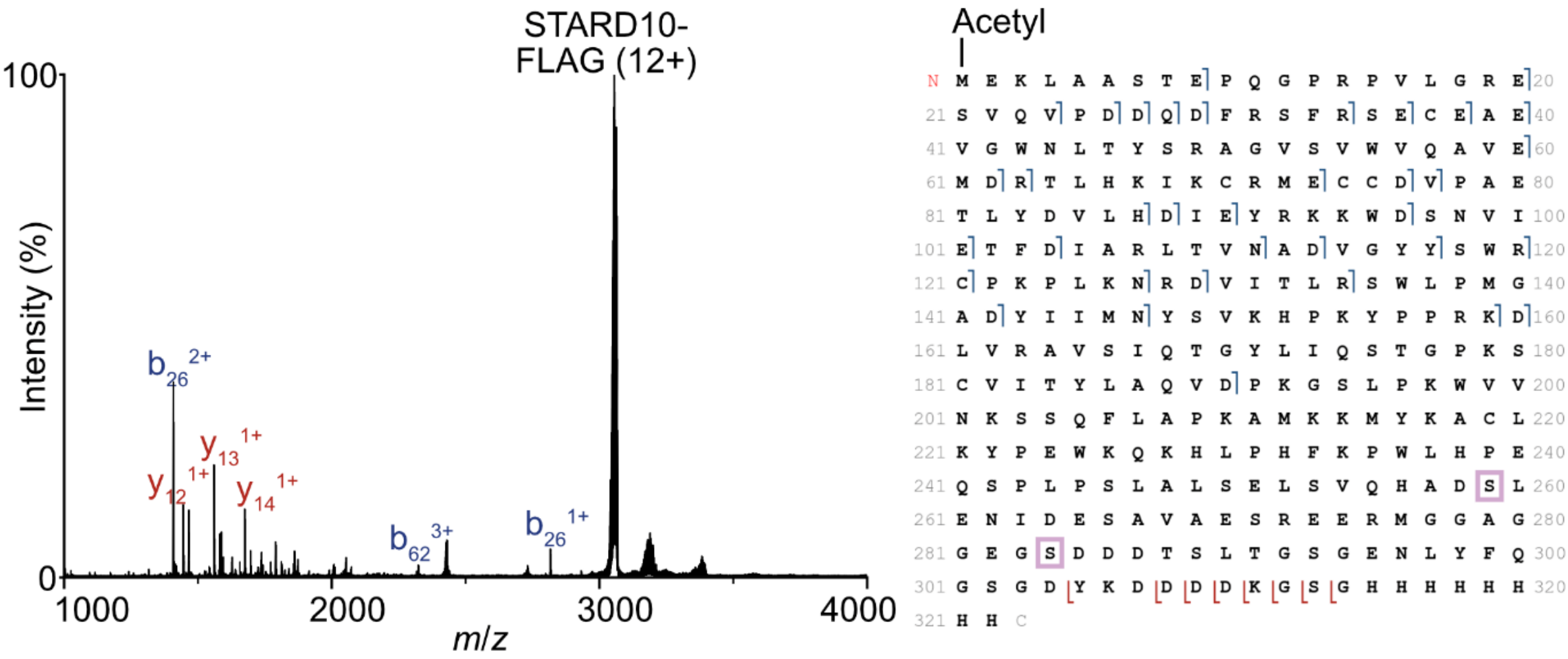

b **Native top-down MS of untagged STARD10**

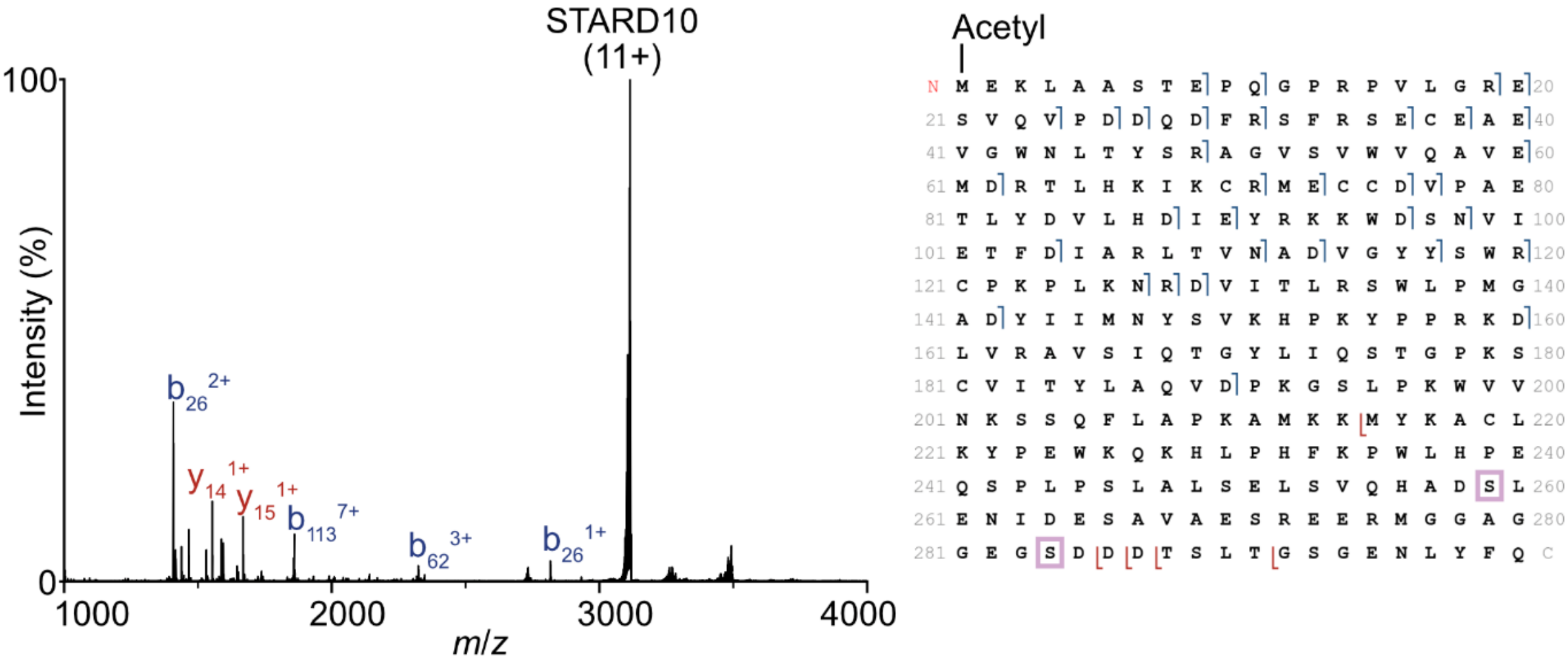

Supplementary Figure 21. Native top-down MS of STARD10. **a.** Fragmentation of FLAG-tagged STARD10 (12+ charge state, HCD 80V). **b.** Fragmentation of untagged STARD10 (11+ charge state, HCD 70 V). Phosphorylated peptides were not detected in either case. No fragments were generated in the region containing the two main phosphorylation sites (S259 and S284; pink). Detected cleavage sites are indicated in the sequence map (blue: b-type ions, red: y-type ions). All detected b-type ions carry an acetyl modification, supporting complete N-acetylation of the protein.

STARD10

MEKLAASTEPQGPRPVLGRESVQVPDDQDFRSFRSECEAEVGWNLTYSRAGVSVWVQAVEMDRTLHKIKCRMECCDVPAETL

YDVLHDIEYRKKWDSNVIETFDIARLTVNADVGYYSWRCPKPLKNRDVITLRSWLPMGADYIIMNYSVKHPKYPPRKDLVRAVSI

QTGYLIQSTGPKSCVITYLAQVDPKGSLPKWVVNKSSQFLAPKAMKKMYKACLKYPEWKQK229HLPHFKPWLHPEQSPLPSLALSE

LSVQHAD**259S**LENIDESAVAESREER276MGGAGGEG**284S**DDDTSLTGSGENLYFQGSGDYKDDDDKGSGHHHHHHHH

Linker TEV protease cleavage site FLAG tag His8 tag

Supplementary Figure 22. Proteomics analysis of STARD10. The sequence map shows two detected peptides with phosphate modifications (underlined). Both peptides were 90–95 % phosphorylated. The main phosphorylation sites are highlighted (Ser259 and Ser284). The measurement was performed once.

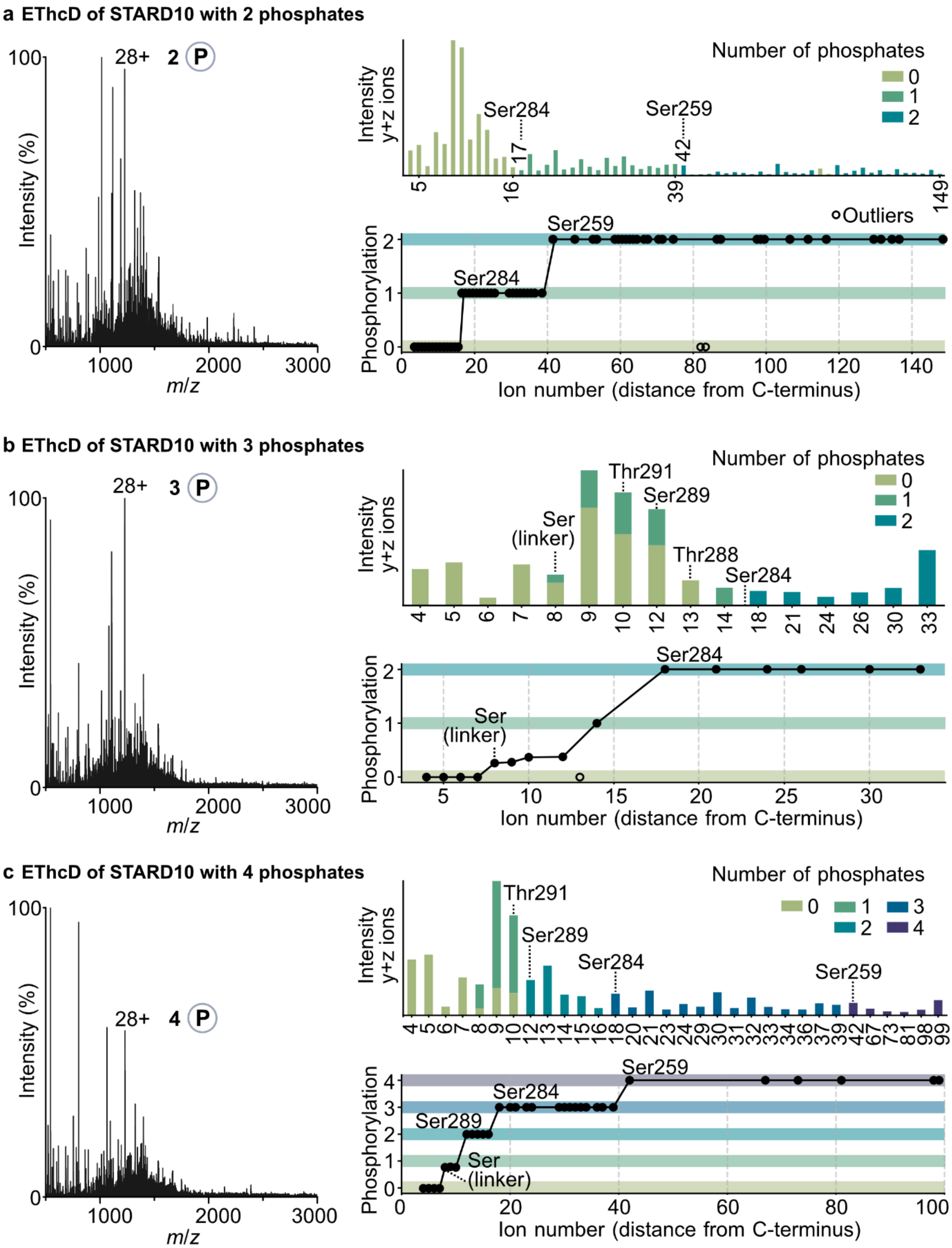

Supplementary Figure 23. Denaturing top-down MS of STARD10 enables mapping of phosphorylation sites. STARD10 (28+ charge state) carrying two (*m*/*z* 1224 ± 1) (**a**), three (*m*/*z* 1228 ± 1) (**b**) or four (*m*/*z* 1230 ± 1) (**c**) phosphates was fragmented by EThcD (1.5 ms, 30 % NCE). By comparing the number of phosphates on y- and z-ions of different length, phosphorylation sites were mapped. Ser284 and Ser259 are constitutively phosphorylated. Partially occupied phosphorylation sites include the serine in the linker (GSG) and three C-terminal residues (Thr288, Ser289, Thr291). Source data are provided as a Source Data file.

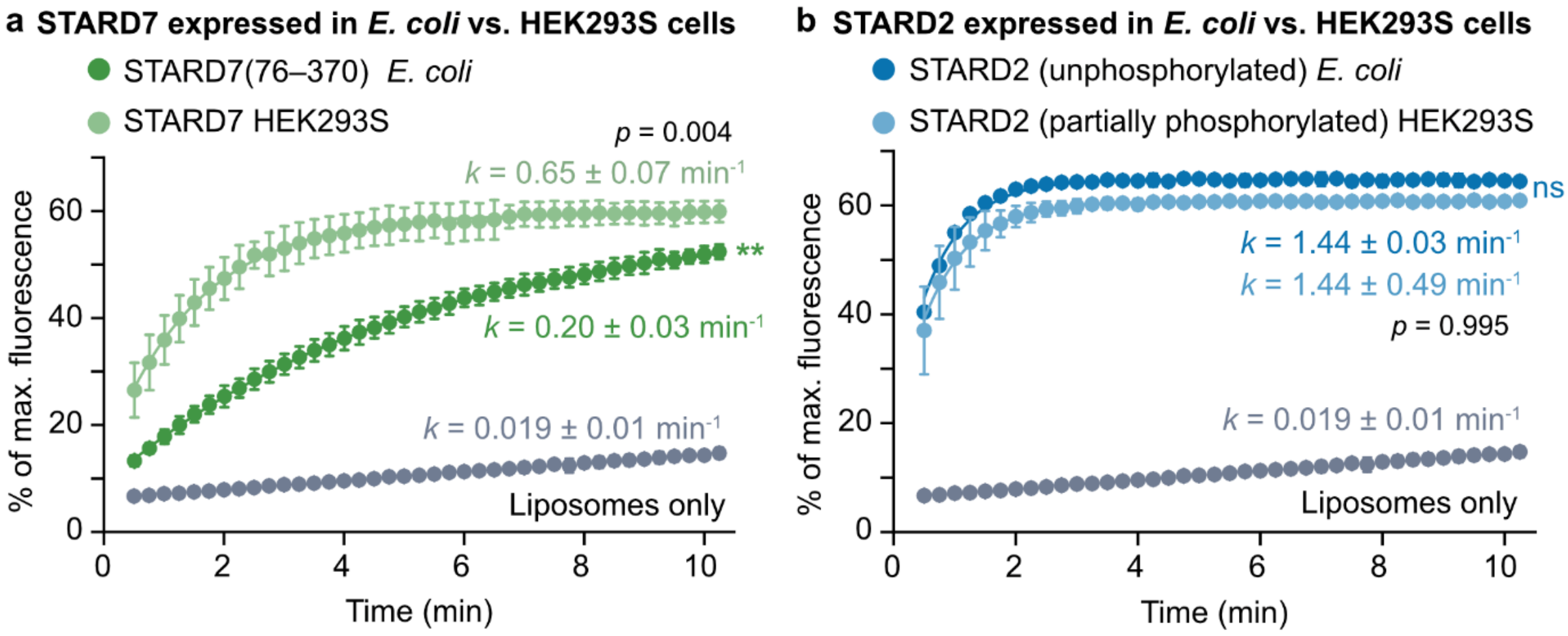

Supplementary Figure 24. Fluorescent lipid transfer assays using lipid transfer proteins recombinantly expressed in *E. coli* or in HEK293S cells. **a.** STARD7 purified from HEK293S cells shows increased lipid transfer activity compared to STARD7(76–370) purified from bacteria. **b.** STARD2 expressed in *E. coli* displays lipid transfer activity comparable to STARD2 produced in HEK293S cells, indicating that partial phosphorylation in human cells does not substantially affect its lipid transfer activity. First-order rate constants (k) were obtained from three technical replicates and are reported as mean ± standard deviation. Graphs show mean values with error bars representing the standard deviation (n = 3). Pairwise statistical significance between the two conditions was assessed using two-sided Welch's t-test. Statistical significance is indicated based on p-values: * $p < 0.05$, ** $p < 0.01$, *** $p < 0.001$; ns = not significant. Source data are provided as a Source Data file.

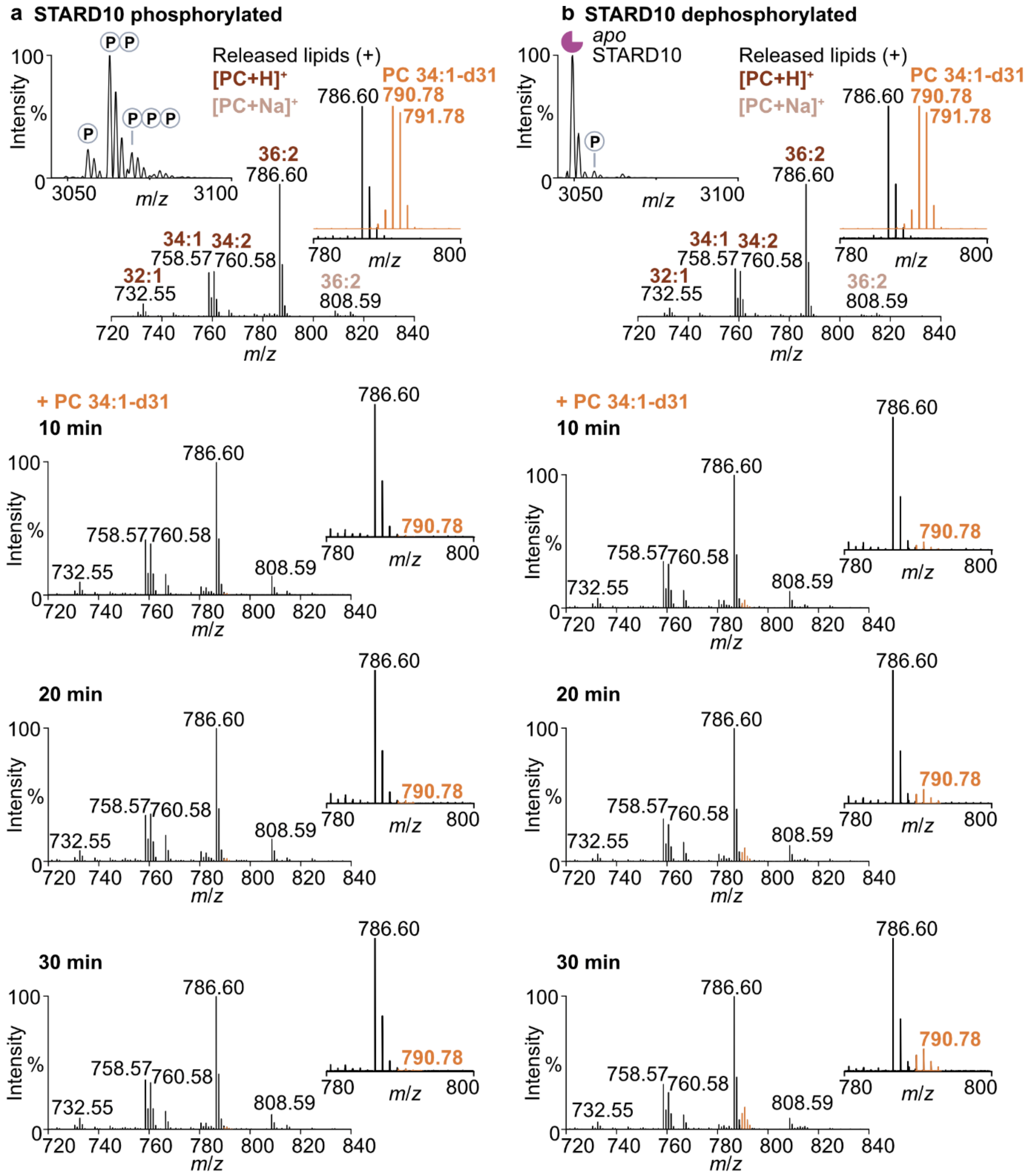

Supplementary Figure 25. Lipid exchange between liposomes containing deuterated PC and STARD10 in distinct phosphorylation states. STARD10 was expressed in HEK293S cells and incubated with liposomes in its native, phosphorylated state (**a**) or following λ-phosphatase treatment (**b**). Top: Native mass spectra of STARD10 recorded with in-source activation (120 V), showing the degree of phosphorylation. MS^2 spectra of STARD10–lipid complexes show copurified lipids prior to liposome addition. The overlaid orange trace represents the mass spectrum of liposomes consisting of 80 % PC 16:0-d31/18:1 (PC 34:1-d31) and 20 % cholesterol. The major species correspond to protonated PC 34:1-d30 (*m/z* 790.78) and PC 34:1-d31 (*m/z* 791.78). Bottom: MS^2 spectra of STARD10–lipid complexes after incubation with deuterated liposomes for 10, 20, or 30 minutes, with binding of deuterated PC 34:1 highlighted in orange. Phosphorylated STARD10 shows minimal exchange of endogenous PC for deuterated PC 34:1, whereas dephosphorylated STARD10 exhibits clear time-dependent

lipid exchange. Measurements were performed in technical triplicate, and the most representative spectra are shown for each time point.

Supplementary Tables

Supplementary Table 1. Expected and measured masses of proteins investigated in this work.

Protein	Expected mass (kDa)	Measured mass (KDa)
**Expressed in *E. coli***		
STARD2	25.038	25.036
STARD2 R78Q	25.009	25.008
STARD7(76–370)	34.891	34.886
STARD7(76–370) R189Q	34.863	34.863
STARD10	33.242	33.238
STARD10 R92Q	33.215	33.214
**Expressed in human cell lines**		
STARD2-FLAG	28.333	28.375 (+42 Da)
STARD7-FLAG (79-370)	37.914	37.914
STARD10-FLAG	36.539	36.741 (+202 Da)

Supplementary Table 2. System setup for molecular dynamics simulations.

<table>
<tr><th>System</th><th>Box dimensions in x,y,z</th><th>Total number of atoms</th><th>Number of water molecules</th><th>Salt concentration</th><th>Components</th></tr>
<tr><td>Wildtype STARD2</td><td rowspan="8">10 x 10 x 10</td><td>98,184</td><td>31,468</td><td rowspan="8">0.15 M NaCl</td><td rowspan="6">The protein bound to one DOPC molecule.</td></tr>
<tr><td>Wildtype STARD7</td><td>98,272</td><td>31,051</td></tr>
<tr><td>Wildtype STARD10</td><td>98,276</td><td>31,113</td></tr>
<tr><td>R78Q STARD2</td><td>98,181</td><td>31,469</td></tr>
<tr><td>R189Q STARD7</td><td>98,272</td><td>31,053</td></tr>
<tr><td>R92Q STARD10</td><td>98,276</td><td>31,115</td></tr>
<tr><td>Wildtype STARD2</td><td>98,261</td><td>31,493</td><td>STARD2 bound to one SAPC molecule</td></tr>
<tr><td>Wildtype STARD7</td><td>98,262</td><td>31,049</td><td>STARD7 bound to one POPC molecule</td></tr>
</table>

Supplementary Table 3. Characterization and relative quantification of phospholipids copurified with STARD2, STARD7, and STARD10 upon expression in HEK293S cells. Bar charts represent the mean ± standard deviation of biological triplicates (n = 3). Source data are provided as a Source Data file.

HEK293S

***m/z* (+)**	**Sum**	**Fatty acids**	**STARD2**	**STARD7**	**STARD10**
704.52	PC 30:1	14:0_16:1			
730.54	PC 32:2	16:1_16:1			
732.55	PC 32:1	16:0_16:1			
758.57	PC 34:2	16:1_18:1			
760.59	PC 34:1	16:0_18:1			
786.60	PC 36:2	18:1_18:1			
788.62	PC 36:1	18:0_18:1			
814.63	PC 38:2	18:1_20:1			

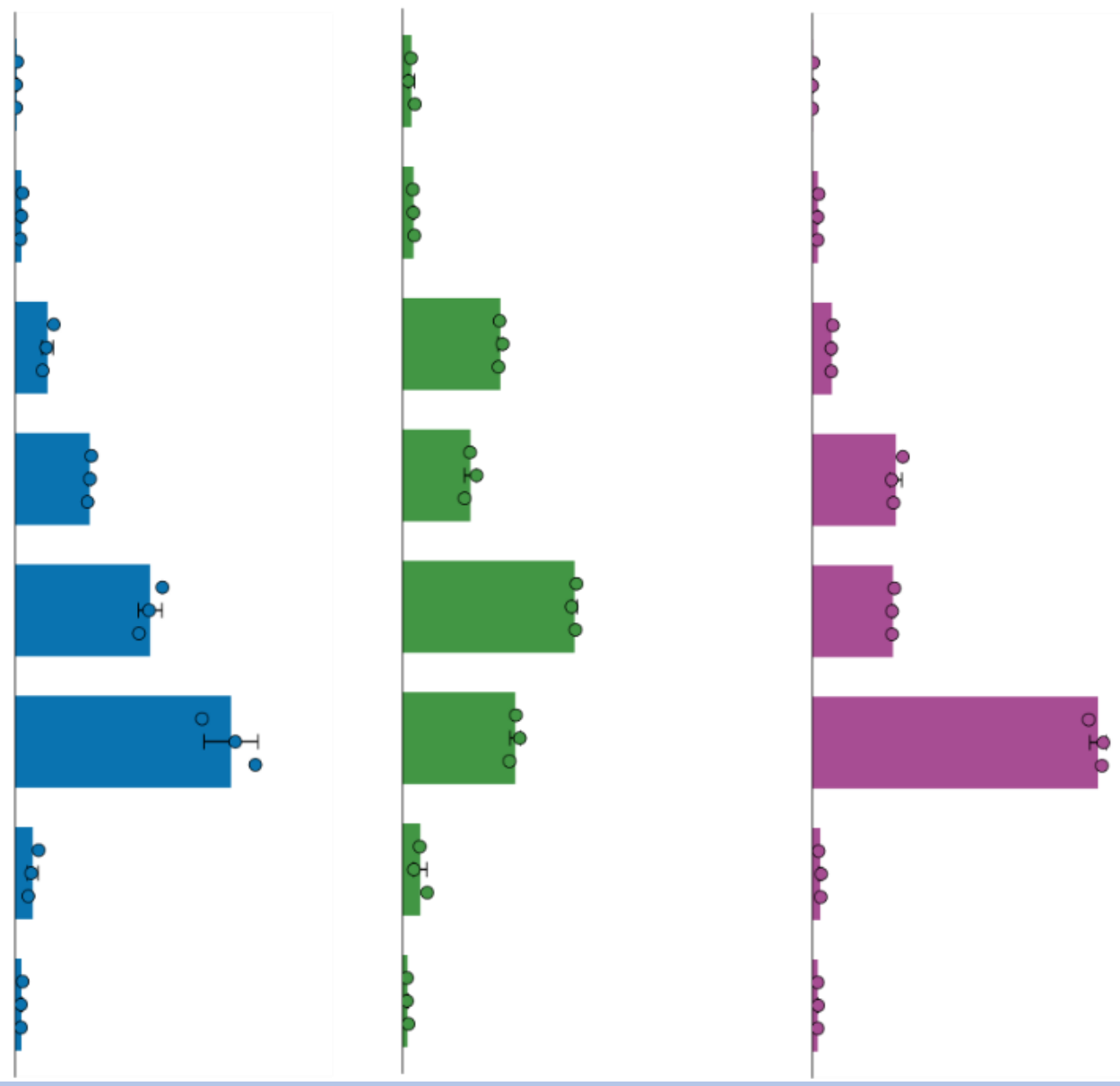

***m/z* (−)**	**Sum**	**Fatty acids**	**STARD10**
698.51	PE-P 34:2	16:1_18:1	
700.53	PE-P 34:1	16:0_18:1	
714.51	PE 34:2	16:1_18:1	
716.52	PE 34:1	16:0_18:1	
724.53	PE-P 36:3	18:1_18:2	
726.54	PE-P 36:2	18:1_18:1	
740.52	PE 36:3	18:1_18:2	
742.54	PE 36:2	18:1_18:1	
773.53	PG 36:2	18:1_18:1	

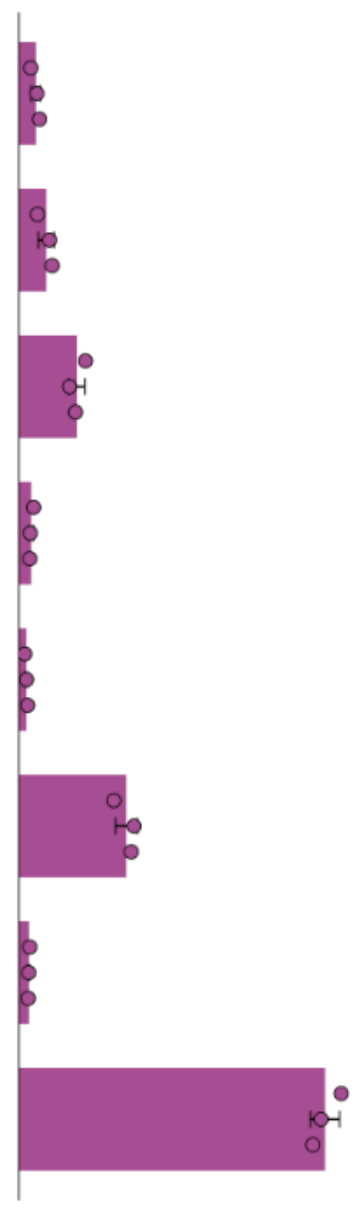

Supplementary Table 4. Characterization and relative quantification of phospholipids copurified with STARD2, STARD7, and STARD10 upon expression in HepG2 cells. Bar charts represent the mean ± standard deviation of biological triplicates (n = 3). Source data are provided as a Source Data file.

HepG2

***m*/*z* (+)**	**Sum**	**Fatty acids**	**STARD2**	**STARD7**	**STARD10**
730.54	PC 32:2	16:1_16:1			
732.55	PC 32:1	16:0_16:1			
756.55	PC 34:3	16:0_18:3<br>16:1_18:2			
758.57	PC 34:2	16:1_18:1<br>16:0_18:2			
760.59	PC 34:1	16:0_18:1			
780.55	PC 36:5	n.a.			
782.57	PC 36:4	n.a.			
784.59	PC 36:3	18:1_18:2			
786.60	PC 36:2	18:1_18:1			
788.62	PC 36:1	18:0_18:1			
804.55	PC 38:7	16:1_22:6			
806.57	PC 38:6	16:0_22:6			
808.59	PC 38:5	18:1_20:4			
810.60	PC 38:4	18:1_20:3			
832.59	PC 40:7	18:1_22:6			

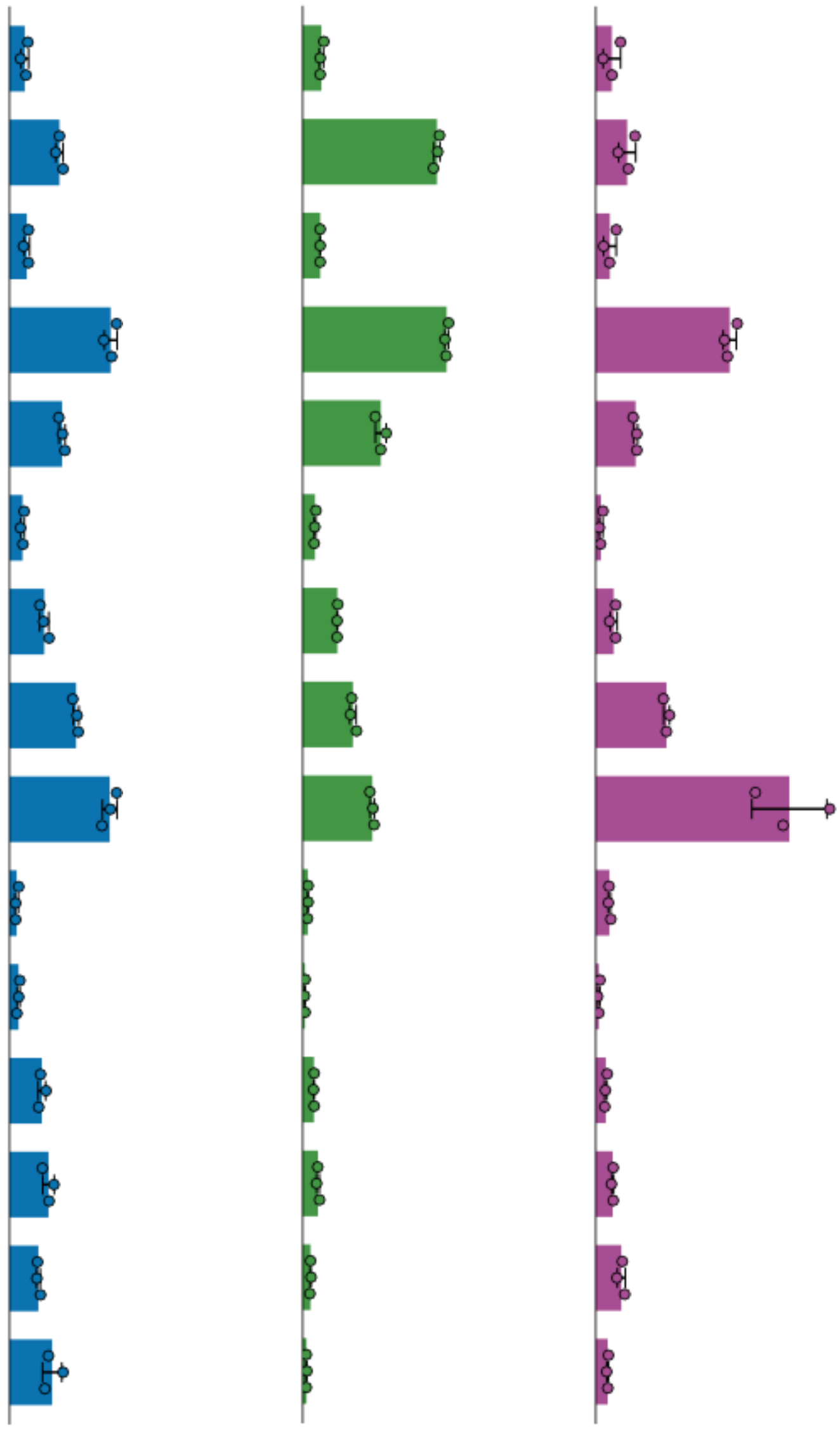

Supplementary Table 4 (continued).

m/*z* (−)	Sum	Fatty acids	STARD10
714.51	PE 34:2	16:1_18:1	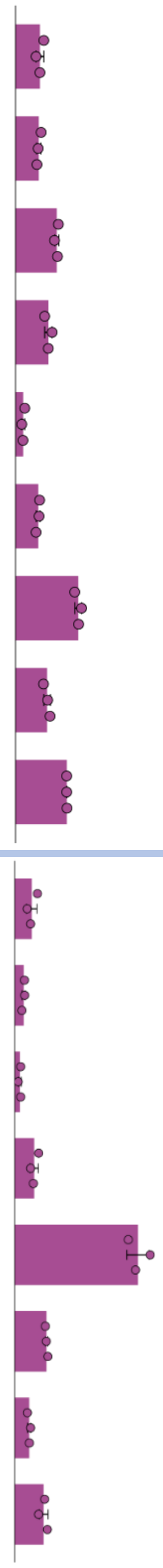
738.51	PE 36:4	18:1_18:3<br>16:0_20:4<br>16:1_20:3	
740.52	PE 36:3	18:1_18:2	
742.54	PE 36:2	18:1_18:1	
760.49	PE 38:7	16:1_22:6	
762.51	PE 38:6	16:0_22:6<br>18:1_20:5	
764.52	PE 38:5	18:1_20:4	
766.54	PE 38:4	18:1_20:3<br>18:0_20:4	
788.52	PE 40:7	18:1_22:6	
745.50	PG 34:2	16:1_18:1	
747.52	PG 34:1	16:0_18:1<br>16:1_18:0	
769.50	PG 36:4	16:1_20:3<br>18:2_18:2	
771.52	PG 36:3	18:1_18:2	
773.53	PG 36:2	18:1_18:1	
797.53	PG 38:4	18:1_20:3	
799.55	PG 38:3	18:1_20:2<br>18:0_20:3	
819.52	PG 40:7	18:1_22:6	

Supplementary Table 5. Precursor isolation windows for dia-PASEF.

Scan Type	Ramp	Mass Start [Da]	Mass End [Da]	Ion mobility Start [Vs/cm^2]	Ion mobility End [Vs/cm^2]
MS[1]	0				
dia-PASEF	1	400	425	0.64	0.83
dia-PASEF	1	600	625	0.83	1.01
dia-PASEF	1	800	825	1.01	1.37
dia-PASEF	2	425	450	0.64	0.85
dia-PASEF	2	625	650	0.85	1.04
dia-PASEF	2	825	850	1.04	1.37
dia-PASEF	3	450	475	0.64	0.87
dia-PASEF	3	650	675	0.87	1.06
dia-PASEF	3	850	875	1.06	1.37
dia-PASEF	4	475	500	0.64	0.90
dia-PASEF	4	675	700	0.90	1.09
dia-PASEF	4	875	900	1.09	1.37
dia-PASEF	5	500	525	0.64	0.92
dia-PASEF	5	700	725	0.92	1.11
dia-PASEF	5	900	925	1.11	1.37
dia-PASEF	6	525	550	0.64	0.94
dia-PASEF	6	725	750	0.94	1.13
dia-PASEF	6	925	950	1.13	1.37
dia-PASEF	7	550	575	0.64	0.97
dia-PASEF	7	750	775	0.97	1.16
dia-PASEF	7	950	975	1.16	1.37
dia-PASEF	8	575	600	0.64	0.99
dia-PASEF	8	775	800	0.99	1.18
dia-PASEF	8	975	1000	1.18	1.37

Supplementary Table 6. Oligonucleotide sequences of gBlocks used for protein expression in *E. coli* and human cell lines.

Description	gBlock sequence
**MBP-STARD2**<br><br>Expressed in *E. coli*<br><br>Plasmid: pET28b with N-terminal MBP (NdeI, XhoI)	GTATTTTCAGGGCCATATGGAATTAGCCGCGGGTTCGTTCTCCGAAG<br>AGCAATTTTGGGAGGCTTGTGCTGAGCTGCAACAACCCGCATTAGC<br>GGGGGCGGACTGGCAGCTTCTTGTTGAGACATCTGGCATCAGCATC<br>TACCGTCTGCTTGACAAGAAGACCGGGCTTTATGAGTACAAGGTGTT<br>TGGAGTGCTTGAGGATTGTAGTCCAACTCTTTTGGCAGACATCTACA<br>TGGATTCGGACTACCGTAAGCAATGGGACCAGTATGTTAAAGAATTAT<br>ATGAGCAAGAATGCAACGGAGAGACTGTGGTCTATTGGGAAGTTAAA<br>TACCCTTTTCCTATGTCCAACCGTGACTATGTCTACCTGCGTCAGCGC<br>CGTGACCTGGACATGGAGGGTCGTAAGATCCATGTCATCTTGGCTC<br>GCAGTACATCGATGCCCCAACTGGGAGAGCGTAGCGGAGTTATCCG<br>CGTAAAACAGTACAAACAAAGTTTGGCGATCGAAAGTGATGGAAAGA<br>AGGGTAGTAAAGTATTTATGTACTATTTCGACAATCCGGGAGGACAAA<br>TTCCCTCATGGTTAATCAATTGGGCCGCGAAAAACGGTGTCCCGAAT<br>TTCCTTAAGGACATGGCTCGCGCGTGCCAGAACTACTTGAAAAAAAC<br>CTAATGACTCGAGCACCACCACC
**MBP-STARD2 R78Q**<br><br>Expressed in *E. coli*<br><br>Plasmid: pET28b with N-terminal MBP (NdeI, XhoI)	GTATTTTCAGGGCCATATGGAATTAGCCGCGGGTTCGTTCTCCGAAG<br>AGCAATTTTGGGAGGCTTGTGCTGAGCTGCAACAACCCGCATTAGC<br>GGGGGCGGACTGGCAGCTTCTTGTTGAGACATCTGGCATCAGCATC<br>TACCGTCTGCTTGACAAGAAGACCGGGCTTTATGAGTACAAGGTGTT<br>TGGAGTGCTTGAGGATTGTAGTCCAACTCTTTTGGCAGACATCTACA<br>TGGATTCGGACTACCAGAAGCAATGGGACCAGTATGTTAAAGAATTAT<br>ATGAGCAAGAATGCAACGGAGAGACTGTGGTCTATTGGGAAGTTAAA<br>TACCCTTTTCCTATGTCCAACCGTGACTATGTCTACCTGCGTCAGCGC<br>CGTGACCTGGACATGGAGGGTCGTAAGATCCATGTCATCTTGGCTC<br>GCAGTACATCGATGCCCCAACTGGGAGAGCGTAGCGGAGTTATCCG<br>CGTAAAACAGTACAAACAAAGTTTGGCGATCGAAAGTGATGGAAAGA<br>AGGGTAGTAAAGTATTTATGTACTATTTCGACAATCCGGGAGGACAAA<br>TTCCCTCATGGTTAATCAATTGGGCCGCGAAAAACGGTGTCCCGAAT<br>TTCCTTAAGGACATGGCTCGCGCGTGCCAGAACTACTTGAAAAAAAC<br>CTAATGACTCGAGCACCACCACC
**STARD2-FLAG-His$_8$**<br><br>Expressed in HEK293S, HepG2<br><br>Plasmid: pHR-CMV-TetO$_2$ with IRES-EmGFP (EcoRI, XhoI)	GCTGTCTAGAGAATTCGCCACCATGGAGCTGGCAGCGGGTTCCTTC<br>TCAGAGGAACAGTTTTGGGAGGCGTGTGCGGAATTGCAGCAGCCTG<br>CACTGGCGGGTGCAGACTGGCAGTTGCTGGTCGAAACTAGCGGCAT<br>CTCCATCTATCGGCTTCTGGACAAAAAAACTGGGCTGTATGAGTACA<br>AGGTGTTTGGGGTTCTTGAAGATTGTAGTCCCACGCTGCTCGCAGA<br>CATATACATGGACTCTGACTATCGGAAACAATGGGATCAGTATGTAAA<br>AGAGCTGTATGAGCAAGAATGTAACGGAGAAACGGTTGTATACTGGG<br>AGGTGAAGTATCCATTCCCCATGAGTAACCGGGATTATGTTTATTTGA<br>GACAGCGGCGGGACCTCGACATGGAGGGTAGGAAAATACATGTGAT<br>TCTCGCCAGAAGTACGTCAATGCCCCAGCTCGGCGAAAGGTCAGGG<br>GTTATCCGAGTAAAACAGTATAAGCAAAGTCTCGCGATAGAGAGCGAT<br>GGAAAGAAGGGGAGTAAAGTGTTTATGTATTACTTCGATAACCCTGG<br>CGGACAGATTCCAAGCTGGTTGATAAATTGGGCCGCGAAGAATGGA<br>GTACCTAATTTTTTGAAGGATATGGCGAGAGCCTGTCAAAACTATCTT<br>AAGAAAACAGGCTCTGGCGAAAATTTGTACTTTCAGGGCTCCGGAG<br>ACTATAAAGACGACGACGATAAGGGCTCTGGCCATCATCATCATCAC<br>CACCACCATTAGTGACTCGAGCGCCCCTCTC

**MBP-STARD7 (76–370)**<br><br>Expressed in *E. coli*<br><br>Plasmid: pET28b with N-terminal MBP (NdeI, XhoI)	GTATTTTCAGGGCCATATGGCTGCGCTTGCGGGAGTATTTGTTTGGG<br>ATGAAGAGCGTATCCAAGAGGAGGAGTTGCAACGTAGCATTAATGAG<br>ATGAAACGCCTTGAAGAGATGTCGAATATGTTCCAGTCCTCGGGGGT<br>GCAACACCATCCTCCAGAGCCAAAGGCTCAAACGGAGGGGAATGAG<br>GATTCAGAAGGTAAGGAACAGCGTTGGGAGATGGTTATGGATAAGAA<br>GCATTTCAAGCTGTGGCGTCGCCCGATCACAGGAACACATTTATACC<br>AGTACCGTGTCTTCGGAACCTATACCGATGTTACACCACGTCAGTTCT<br>TCAACGTCCAGCTTGATACTGAGTACCGCAAAAAGTGGGACGCACT<br>GGTGATTAAGTTGGAGGTCATCGAACGCGATGTCGTGTCTGGATCAG<br>AAGTTCTGCACTGGGTAACGCACTTCCCGTATCCCATGTATTCACGT<br>GACTACGTTTACGTCCGCCGTTACTCCGTTGATCAAGAAAATAATATG<br>ATGGTACTGGTGTCTCGTGCGGTTGAGCATCCAAGTGTACCAGAGA<br>GCCCCGAGTTCGTCCGTGTACGTTCTTACGAAAGCCAGATGGTCATT<br>CGCCCTCATAAGTCATTTGATGAGAACGGTTTCGACTATCTTCTGACA<br>TATTCTGATAATCCACAAACCGTTTTTCCGCGCTATTGCGTCTCCTGG<br>ATGGTGTCGAGCGGTATGCCGGACTTCCTGGAGAAATTGCACATGG<br>CTACTTTGAAGGCGAAAAATATGGAGATTAAGGTAAAAGACTATATCA<br>GTGCAAAACCACTGGAAATGTCAAGTGAGGCGAAAGCAACAAGCCA<br>AAGCTCAGAACGTAAGAATGAGGGTAGTTGCGGGCCAGCACGTATC<br>GAATACGCCTAATGACTCGAGCACCACCACC
**MBP-STARD7 (76–370) R189Q**<br><br>Expressed in *E. coli*<br><br>Plasmid: pET28b with N-terminal MBP (NdeI, XhoI)	GTATTTTCAGGGCCATATGGCTGCGCTTGCGGGAGTATTTGTTTGGG<br>ATGAAGAGCGTATCCAAGAGGAGGAGTTGCAACGTAGCATTAATGAG<br>ATGAAACGCCTTGAAGAGATGTCGAATATGTTCCAGTCCTCGGGGGT<br>GCAACACCATCCTCCAGAGCCAAAGGCTCAAACGGAGGGGAATGAG<br>GATTCAGAAGGTAAGGAACAGCGTTGGGAGATGGTTATGGATAAGAA<br>GCATTTCAAGCTGTGGCGTCGCCCGATCACAGGAACACATTTATACC<br>AGTACCGTGTCTTCGGAACCTATACCGATGTTACACCACGTCAGTTCT<br>TCAACGTCCAGCTTGATACTGAGTACCAGAAAAAGTGGGACGCACTG<br>GTGATTAAGTTGGAGGTCATCGAACGCGATGTCGTGTCTGGATCAGA<br>AGTTCTGCACTGGGTAACGCACTTCCCGTATCCCATGTATTCACGTG<br>ACTACGTTTACGTCCGCCGTTACTCCGTTGATCAAGAAAATAATATGA<br>TGGTACTGGTGTCTCGTGCGGTTGAGCATCCAAGTGTACCAGAGAG<br>CCCCGAGTTCGTCCGTGTACGTTCTTACGAAAGCCAGATGGTCATTC<br>GCCCTCATAAGTCATTTGATGAGAACGGTTTCGACTATCTTCTGACAT<br>ATTCTGATAATCCACAAACCGTTTTTCCGCGCTATTGCGTCTCCTGGA<br>TGGTGTCGAGCGGTATGCCGGACTTCCTGGAGAAATTGCACATGGC<br>TACTTTGAAGGCGAAAAATATGGAGATTAAGGTAAAAGACTATATCAG<br>TGCAAAACCACTGGAAATGTCAAGTGAGGCGAAAGCAACAAGCCAA<br>AGCTCAGAACGTAAGAATGAGGGTAGTTGCGGGCCAGCACGTATCG<br>AATACGCCTAATGACTCGAGCACCACCACC
**STARD7-FLAG-His$_8$**<br><br>Expressed in HEK293S, HepG2<br><br>Plasmid: pHR-CMV-TetO$_2$ with IRES-EmGFP (EcoRI, XhoI)	GCTGTCTAGAGAATTCGCCACCATGTTGCCGCGACGCCTTTTGGCA<br>GCATGGCTCGCAGGTACAAGAGGTGGTGGTTTGCTGGCGCTTTTGG<br>CAAATCAATGTCGGTTTGTCACGGGCCTCCGCGTCAGGAGGGCACA<br>ACAGATTGCACAGTTGTACGGAAGACTGTACTCAGAGAGTTCCCGCA<br>GGGTGCTGCTCGGACGCCTGTGGCGCAGGCTTCACGGCAGACCCG<br>GGCATGCCTCCGCTTTGATGGCAGCGCTGGCCGGTGTTTTCGTGTG<br>GGACGAAGAAAGGATCCAGGAAGAGGAGCTCCAGAGAAGTATAAAC<br>GAGATGAAGCGCCTGGAGGAAATGAGCAACATGTTCCAGAGCAGTG<br>GGGTGCAACACCATCCACCTGAACCGAAAGCACAAACCGAAGGAAA<br>CGAAGACAGCGAAGGTAAGGAGCAACGGTGGGAAATGGTGATGGAT<br>AAAAAGCACTTTAAGCTCTGGCGAAGGCCCATCACCGGCACTCATCT<br>CTACCAATACCGAGTCTTTGGCACCTACACTGATGTAACACCCCGGC<br>AATTCTTTAATGTCCAGCTGGACACGGAGTACCGCAAAAAATGGGAC<br>GCCCTTGTAATTAAGCTGGAAGTTATCGAGAGAGATGTCGTCTCAGG<br>ATCCGAGGTTCTTCACTGGGTGACACATTTTCCCTATCCCATGTACTC

	CCGCGACTATGTGTACGTTCGGCGGTATAGCGTTGACCAAGAGAATA<br>ATATGATGGTCCTCGTGTCTCGCGCGGTAGAACATCCTAGCGTGCCA<br>GAATCACCGGAGTTTGTCCGCGTACGCAGTTACGAGTCTCAAATGGT<br>CATTCGACCTCACAAGTCATTTGACGAGAATGGCTTCGATTACCTGC<br>TCACTTATTCAGATAACCCTCAAACAGTATTTCCACGCTACTGCGTCT<br>CCTGGATGGTGTCATCAGGTATGCCCGATTTTCTTGAGAAGCTCCAC<br>ATGGCAACTCTCAAAGCGAAAAACATGGAGATCAAGGTTAAAGACTA<br>CATATCTGCAAAGCCACTGGAGATGAGTTCTGAGGCGAAAGCAACAA<br>GCCAGTCAAGCGAAAGGAAGAACGAGGGCAGTTGTGGACCAGCTC<br>GGATCGAATACGCCGGCTCTGGCGAAAATTTGTACTTTCAGGGCTCC<br>GGAGACTATAAAGACGACGACGATAAGGGCTCTGGCCATCATCATCA<br>TCACCACCACCATTAGTGACTCGAGCGCCCCTCTC
**MBP-STARD10**<br><br>Expressed in *E. coli*<br><br>Plasmid: pET28b (NcoI, XhoI)*	AAGAAGGAGATATACCATGGGTTCTTCTCACCATCACCATCACCACG<br>GTTCTTCTATGAAAATCGAAGAAGGTAAACTGGTAATCTGGATTAACG<br>GCGATAAAGGCTATAACGGTCTCGCTGAAGTCGGTAAGAAATTCGAG<br>AAAGATACCGGAATTAAAGTCACCGTTGAGCATCCGGATAAACTGGA<br>AGAGAAATTCCCACAGGTTGCGGCAACTGGCGATGGCCCTGACATT<br>ATCTTCTGGGCACACGACCGCTTTGGTGGCTACGCTCAATCTGGCCT<br>GTTGGCTGAAATCACCCCGGACAAAGCGTTCCAGGACAAGCTGTAT<br>CCGTTTACCTGGGATGCCGTACGTTACAACGGCAAGCTGATTGCTTA<br>CCCGATCGCTGTTGAAGCGTTATCGCTGATTTATAACAAAGATCTGCT<br>GCCGAACCCGCCAAAAACCTGGGAAGAGATCCCGGCGCTGGATAAA<br>GAACTGAAAGCGAAAGGTAAGAGCGCGCTGATGTTCAACCTGCAAG<br>AACCGTACTTCACCTGGCCGCTGATTGCTGCTGACGGGGGTTATGC<br>GTTCAAGTATGAAAACGGCAAGTACGACATTAAAGACGTGGGCGTGG<br>ATAACGCTGGCGCGAAAGCGGGTCTGACCTTCCTGGTTGACCTGAT<br>TAAAAACAAACACATGAATGCAGACACCGATTACTCCATCGCAGAAG<br>CTGCCTTTAATAAAGGCGAAACAGCGATGACCATCAACGGCCCGTG<br>GGCATGGTCCAACATCGACACCAGCAAAGTGAATTATGGTGTAACGG<br>TACTGCCGACCTTCAAGGGTCAACCATCCAAACCGTTCGTTGGCGT<br>GCTGAGCGCAGGTATTAACGCCGCCAGTCCGAACAAAGAGCTGGCA<br>AAAGAGTTCCTCGAAAACTATCTGCTGACTGATGAAGGTCTGGAAGC<br>GGTTAATAAAGACAAACCGCTGGGTGCCGTAGCGCTGAAGTCTTACG<br>AGGAAGAGTTGGCGAAAGATCCACGTATTGCCGCCACTATGGAAAAC<br>GCCCAGAAAGGTGAAATCATGCCGAACATCCCGCAGATGTCCGCTTT<br>CTGGTATGCCGTGCGTACTGCGGTGATCAACGCCGCCAGCGGTCGT<br>CAGACTGTCGATGAAGCCCTGAAAGACGCGCAGACTAATTCGAGCT<br>CGAACAACAACAACAATAACAATAACAACAACCTCGGGATCGAGGAA<br>AACCTGTATTTTCAGGGCCATATGGAAAAACTTGCCGCTTCAACGGA<br>ACCTCAGGGACCACGTCCGGTACTGGGACGTGAATCCGTACAAGTC<br>CCTGATGACCAAGACTTCCGCAGCTTTCGTTCCGAGTGCGAGGCGG<br>AAGTAGGTTGGAATCTTACTTACTCTCGCGCCGGTGTATCCGTGTGG<br>GTTCAAGCAGTTGAAATGGACCGTACCCTGCATAAAATCAAGTGTCG<br>TATGGAGTGCTGCGATGTCCCCGCAGAGACGCTTTATGATGTCCTGC<br>ATGATATCGAGTACCGTAAGAAGTGGGATAGTAACGTCATTGAAACAT<br>TCGACATCGCCCGCTTGACAGTGAATGCTGACGTCGGGTACTATAGT<br>TGGCGTTGCCCGAAACCGTTGAAAAATCGTGATGTAATCACTTTACG<br>CTCGTGGCTTCCAATGGGCGCTGACTATATCATTATGAATTACAGCGT<br>TAAGCATCCTAAGTATCCTCCCCGTAAAGATTTGGTCCGCGCGGTTA<br>GCATCCAAACGGGTTACCTTATTCAGTCCACTGGACCGAAATCTTGT<br>GTCATCACCTATCTTGCGCAAGTGGACCCTAAAGGTAGCTTGCCTAA<br>ATGGGTTGTGAACAAAAGTAGTCAATTTCTGGCGCCAAAAGCAATGA<br>AGAAAATGTACAAGGCTTGTTTGAAGTACCCAGAATGGAAGCAGAAG<br>CACTTACCTCATTTCAAACCTTGGCTGCATCCTGAGCAAAGCCCCTT<br>GCCGTCTTTAGCGCTGAGTGAGTTATCGGTACAGCATGCAGATTCAC

	TTGAGAACATTGATGAATCGGCTGTCGCGGAGTCTCGTGAAGAACGT<br>ATGGGCGGGGCGGGTGGTGAAGGGTCGGACGACGATACCAGCCTT<br>ACTTAATGACTCGAGCACCACCACC
**MBP-<br>STARD10<br>R92Q**<br><br>Expressed in<br>*E. coli*<br><br>Plasmid:<br>pET28b with<br>N-terminal<br>MBP (NdeI,<br>XhoI)	GTATTTTCAGGGCCATATGGAAAAACTTGCCGCTTCAACGGAACCTC<br>AGGGACCACGTCCGGTACTGGGACGTGAATCCGTACAAGTCCCTGA<br>TGACCAAGACTTCCGCAGCTTTCGTTCCGAGTGCGAGGCGGAAGTA<br>GGTTGGAATCTTACTTACTCTCGCGCCGGTGTATCCGTGTGGGTTCA<br>AGCAGTTGAAATGGACCGTACCCTGCATAAAATCAAGTGTCGTATGG<br>AGTGCTGCGATGTCCCCGCAGAGACGCTTTATGATGTCCTGCATGAT<br>ATCGAGTACCAGAAGAAGTGGGATAGTAACGTCATTGAAACATTCGA<br>CATCGCCCGCTTGACAGTGAATGCTGACGTCGGGTACTATAGTTGGC<br>GTTGCCCGAAACCGTTGAAAAATCGTGATGTAATCACTTTACGCTCG<br>TGGCTTCCAATGGGCGCTGACTATATCATTATGAATTACAGCGTTAAG<br>CATCCTAAGTATCCTCCCCGTAAAGATTTGGTCCGCGCGGTTAGCAT<br>CCAAACGGGTTACCTTATTCAGTCCACTGGACCGAAATCTTGTGTCA<br>TCACCTATCTTGCGCAAGTGGACCCTAAAGGTAGCTTGCCTAAATGG<br>GTTGTGAACAAAAGTAGTCAATTTCTGGCGCCAAAAGCAATGAAGAA<br>AATGTACAAGGCTTGTTTGAAGTACCCAGAATGGAAGCAGAAGCACT<br>TACCTCATTTCAAACCTTGGCTGCATCCTGAGCAAAGCCCCTTGCCG<br>TCTTTAGCGCTGAGTGAGTTATCGGTACAGCATGCAGATTCACTTGA<br>GAACATTGATGAATCGGCTGTCGCGGAGTCTCGTGAAGAACGTATG<br>GGCGGGGCGGGTGGTGAAGGGTCGGACGACGATACCAGCCTTACT<br>TAATGACTCGAGCACCACCACC
**STARD10-<br>FLAG-His$_8$**<br><br>Expressed in<br>HEK293S,<br>HepG2<br><br>Plasmid: pHR-<br>CMV-TetO$_2$<br>with IRES-<br>EmGFP<br>(EcoRI, XhoI)	GCTGTCTAGAGAATTCGCCACCATGGAGAAGTTGGCCGCAAGTACA<br>GAGCCCCAAGGCCCGAGGCCGGTCCTCGGGAGAGAAAGCGTGCAA<br>GTTCCAGACGATCAGGACTTTCGGAGCTTCCGAAGTGAATGTGAAG<br>CTGAGGTGGGGTGGAACTTGACATATAGCAGGGCGGGTGTCAGCGT<br>ATGGGTACAAGCTGTAGAGATGGACCGAACCTTGCATAAAATAAAATG<br>CCGCATGGAGTGTTGTGACGTACCAGCCGAGACACTCTACGATGTG<br>CTGCACGATATCGAATACAGGAAAAAGTGGGATAGTAACGTCATAGAA<br>ACGTTTGACATCGCCAGACTCACAGTCAATGCGGACGTTGGGTATTA<br>CTCCTGGCGGTGTCCCAAACCACTGAAGAATAGAGATGTAATTACGC<br>TCAGATCATGGCTTCCGATGGGTGCGGATTACATTATAATGAATTACT<br>CAGTTAAGCATCCCAAATACCCACCGAGAAAGGACCTGGTGCGGGC<br>TGTATCCATTCAGACGGGTTATTTGATACAGTCAACGGGGCCCAAATC<br>ATGCGTTATCACCTATCTTGCACAAGTAGATCCGAAAGGGTCCTTGC<br>CGAAGTGGGTAGTTAATAAATCCAGTCAATTTTTGGCGCCCAAAGCG<br>ATGAAGAAGATGTATAAAGCTTGCCTTAAGTATCCAGAATGGAAGCAA<br>AAACACCTTCCACATTTCAAGCCGTGGCTCCACCCAGAGCAGAGTC<br>CTCTCCCCAGCCTCGCCTTGAGCGAACTTTCTGTACAGCATGCCGA<br>CTCACTCGAAAATATTGACGAATCCGCCGTCGCAGAAAGCCGGGAA<br>GAGCGCATGGGGGGCGCGGGTGGTGAAGGCTCCGACGATGACACT<br>AGTCTGACGGGCTCTGGCGAAAATTTGTACTTTCAGGGCTCCGGAG<br>ACTATAAAGACGACGACGATAAGGGCTCTGGCCATCATCATCATCAC<br>CACCACCATTAGTGACTCGAGCGCCCCTCTC

* gBlock incorporates MBP sequence with NdeI restriction site, used for generation of pET28b plasmid with N-terminal MBP.

Supplementary Table 7. Summary table for molecular dynamics simulations.

**Reliability and reproducibility checklist for molecular dynamics simulations**<br>***All boxes must be marked YES by acceptance unless "Response not needed if No".**	**Yes**	**No**	**Response (Please state where this information can be found in the text)**
**1. Convergence of simulations and analysis**			
1a. Is an evaluation presented in the text to show that the property being measured has equilibrated in the simulations (*e.g.* time-course analysis)?	☒	☐	Time course analysis of the properties of interest is presented in Figure2 and the associated text.
1b. Then, is it described in the text how simulations are split into equilibration and production runs and how much data were analyzed from production runs?	☒	☐	This information is presented in the methods section and Figure 2.
1c. Are there at least 3 simulations per simulation condition with statistical analysis?	☒	☐	The methods section states the replicates and the statistical analysis.
1d. Is evidence provided in the text that the simulation results presented are independent of initial configuration?	☐	☒	Since only unbiased simulations are carried out, the simulation results depend on the initial configuration chosen. Multiple replicates started with different initial velocities were carried out to lessen this issue.
**2. Connection to experiments**			
2a. Are calculations provided that can connect to experiments (*e.g.* loss or gain in function from mutagenesis, binding assays, NMR chemical shifts, J-couplings, SAXS curves, interaction distances or FRET distances, structure factors, diffusion coefficients, bulk modulus and other mechanical properties, *etc.*)?	☒	☐	Comparison with mass spectrometry data, including loss-of-function mutant is shown in Figure 2 and the associated text.
**3. Method choice**			
3a. Do simulations contain membranes, membrane proteins, intrinsically disordered proteins, glycans, nucleic acids, polymers, or cryptic ligand binding?	☒	☐	The simulations contain lipids in the protein cavity, but not in a bilayer-like membrane assembly.
3b. Is it described in the text whether the accuracy of the chosen model(s) is sufficient to address the question(s) under investigation (e.g. all-atom vs. coarse-grained models, fixed charge vs. polarizable force fields, implicit vs. explicit solvent or membrane, force field and water model, etc.)?	☒	☐	The text states the use of all-atom molecular dynamics simulations to gain an understanding of the mechanism at a molecular level, which is the appropriate

			level-of-theory for these investigations.
3c. Is the timescale of the event(s) under investigation beyond the brute-force MD simulation timescale in this study that enhanced sampling methods are needed?	☐	☒	Enhanced sampling is not needed. No explicit free energy calculations have been carried out.
If **YES**, are the parameters and convergence criteria for the enhanced sampling method clearly stated?	☐	☐	
If **NO,** is the evidence provided in the text?	☒	☐	Yes, only equilibrium thermodynamic properties are reported/measured.
**4. Code and reproducibility**			
4a. Is a table provided describing the system setup that includes simulation box dimensions, total number of atoms, total number of water molecules, salt concentration, lipid composition (number of molecules and type)?	☒	☐	The system setup is described in Table S2.
4b. Is it described in the text what simulation and analysis software and which versions are used?	☒	☐	The software used is described in the methods section.
4c. Are other parameters for the system setup described in the text, such as protonation state, type of structural restraints if applied, nonbonded cutoff, thermostat and barostat, etc.?	☒	☐	Parameters for the system setup are described in the methods section.
4d. Are initial coordinate and simulation input files and a coordinate file of the final output provided as supplementary files or in a public repository?	☒	☐	All files are provided via Figshare.
4e. Is there custom code or custom force field parameters?	☐	☒	Response not needed if **No**
If **YES**, are they provided as supplementary files or in a public repository?	☐	☐	

Supplementary References

1 Kapushesky, M. *et al.* Gene expression atlas at the European bioinformatics institute. *Nucleic Acids Res.* **38**, D690–698 (2010).

2 Fahy, E. Biomarkers of NAFLD progression: a lipidomics approach to an epidemic. Part 1: Liver (2018). Metabolomics Workbench. Study ID: ST000915. Project DOI: https://doi.org/10.21228/M8V961

3 Koay, Y. C. Metabolomics studies on human cardiac samples (2023). Metabolomics Workbench. Study ID: ST003104. Project DOI: https://doi.org/10.21228/M8N42X
