# Supplementary material for: Differential lipid selectivity of StARD phospholipid transporters revealed by native MS
Source: Nat Commun. 2026 Aug 22;17:10054. doi: 10.1038/s41467-026-76988-1 (PMC13594186; doi:10.1038/s41467-026-76988-1)
Supplement: Supplementary file 2 [file 41467_2026_76988_MOESM2_ESM.pdf]

nature portfolio

Corresponding author(s): Prof. Dame Carol V. Robinson
Dr. Carla Kirschbaum

Last updated by author(s): Jul 24, 2026

# Reporting Summary

Nature Portfolio wishes to improve the reproducibility of the work that we publish. This form provides structure for consistency and transparency in reporting. For further information on Nature Portfolio policies, see our Editorial Policies and the Editorial Policy Checklist.

## Statistics

For all statistical analyses, confirm that the following items are present in the figure legend, table legend, main text, or Methods section.

| n/a | Confirmed | |
|---|---|---|
| ☐ | ☒ | The exact sample size (*n*) for each experimental group/condition, given as a discrete number and unit of measurement |
| ☐ | ☒ | A statement on whether measurements were taken from distinct samples or whether the same sample was measured repeatedly |
| ☐ | ☒ | The statistical test(s) used AND whether they are one- or two-sided<br>*Only common tests should be described solely by name; describe more complex techniques in the Methods section.* |
| ☒ | ☐ | A description of all covariates tested |
| ☐ | ☒ | A description of any assumptions or corrections, such as tests of normality and adjustment for multiple comparisons |
| ☐ | ☒ | A full description of the statistical parameters including central tendency (e.g. means) or other basic estimates (e.g. regression coefficient) AND variation (e.g. standard deviation) or associated estimates of uncertainty (e.g. confidence intervals) |
| ☐ | ☒ | For null hypothesis testing, the test statistic (e.g. *F*, *t*, *r*) with confidence intervals, effect sizes, degrees of freedom and *P* value noted<br>*Give P values as exact values whenever suitable.* |
| ☒ | ☐ | For Bayesian analysis, information on the choice of priors and Markov chain Monte Carlo settings |
| ☒ | ☐ | For hierarchical and complex designs, identification of the appropriate level for tests and full reporting of outcomes |
| ☐ | ☒ | Estimates of effect sizes (e.g. Cohen's *d*, Pearson's *r*), indicating how they were calculated |

*Our web collection on statistics for biologists contains articles on many of the points above.*

## Software and code

Policy information about availability of computer code

| | |
|---|---|
| Data collection | Thermo XCalibur 4.6.67.17, Bruker HyStar 6.3, BMG Labtech CLARIOstar 5.60 R2 |
| Data analysis | Thermo XCalibur 4.5.474.0 Qual Browser, MSConvert (ProteoWizard 3.0), LipiDex 1.1, MZmine 3, FragPipe 23.1, MSFragger 4.3, DIA-NN 1.8.2 beta 8, UniDec 8.1.0, Origin 2025b, precisION 0.2.0, Python 3.11.3, Matplotlib 3.10.3, GROMACS 2023.3 |

For manuscripts utilizing custom algorithms or software that are central to the research but not yet described in published literature, software must be made available to editors and reviewers. We strongly encourage code deposition in a community repository (e.g. GitHub). See the Nature Portfolio guidelines for submitting code & software for further information.

## Data

Policy information about availability of data

All manuscripts must include a data availability statement. This statement should provide the following information, where applicable:

- Accession codes, unique identifiers, or web links for publicly available datasets
- A description of any restrictions on data availability
- For clinical datasets or third party data, please ensure that the statement adheres to our policy

Source data are provided with this paper. Supplementary figures and tables are provided in the Supplementary Information. Raw files generated in this study have been deposited in the Figshare repository under DOI: 10.6084/m9.figshare.31056178 [https://doi.org/10.6084/m9.figshare.31056178]. The lipidomics data generated in this study have been deposited to the MassIVE database under accession code MSV000102576 [https://massive.ucsd.edu/ProteoSAFe/dataset.jsp?

accession=MSV000102576]. Proteomics data generated in this study have been deposited to the ProteomeXchange Consortium via the PRIDE partner repository under accession code PXD072869 [http://proteomecentral.proteomexchange.org/cgi/GetDataset?ID=PXD072869].

## Research involving human participants, their data, or biological material

Policy information about studies with human participants or human data. See also policy information about sex, gender (identity/presentation), and sexual orientation and race, ethnicity and racism.

| | |
|---|---|
| Reporting on sex and gender | N/A |
| Reporting on race, ethnicity, or other socially relevant groupings | N/A |
| Population characteristics | N/A |
| Recruitment | N/A |
| Ethics oversight | N/A |

Note that full information on the approval of the study protocol must also be provided in the manuscript.

# Field-specific reporting

Please select the one below that is the best fit for your research. If you are not sure, read the appropriate sections before making your selection.

☒ Life sciences ☐ Behavioural & social sciences ☐ Ecological, evolutionary & environmental sciences

For a reference copy of the document with all sections, see nature.com/documents/nr-reporting-summary-flat.pdf

# Life sciences study design

All studies must disclose on these points even when the disclosure is negative.

| | |
|---|---|
| Sample size | Proteins were expressed once in E. coli (n = 1 biological replicate) for hypothesis generation. For protein expression in human cell lines, n = 3 biological replicates were performed to enable statistical analysis of lipid enrichment. For siRNA-mediated protein knockdown, n = 6 biological replicates were performed to improve detection of minor changes in the cellular lipidome. |
| Data exclusions | No data were excluded from the analyses. |
| Replication | Mass spectrometry measurements used for quantification were acquired once for each biological replicate. Proteins expressed in E. coli were measured in technical triplicate. Qualitative measurements were performed only once. |
| Randomization | Samples were divided based on treatment. |
| Blinding | The investigators were not blinded because mass spectrometry analysis does not use group allocation. |

# Reporting for specific materials, systems and methods

We require information from authors about some types of materials, experimental systems and methods used in many studies. Here, indicate whether each material, system or method listed is relevant to your study. If you are not sure if a list item applies to your research, read the appropriate section before selecting a response.

## Materials & experimental systems

| n/a | Involved in the study | |
|---|---|---|
| ☒ | ☐ | Antibodies |
| ☐ | ☒ | Eukaryotic cell lines |
| ☒ | ☐ | Palaeontology and archaeology |
| ☒ | ☐ | Animals and other organisms |
| ☒ | ☐ | Clinical data |
| ☒ | ☐ | Dual use research of concern |
| ☒ | ☐ | Plants |

## Methods

| n/a | Involved in the study | |
|---|---|---|
| ☒ | ☐ | ChIP-seq |
| ☒ | ☐ | Flow cytometry |
| ☒ | ☐ | MRI-based neuroimaging |

## Eukaryotic cell lines

Policy information about cell lines and Sex and Gender in Research

| | |
|---|---|
| Cell line source(s) | HEK293S GNTI-cells (ATCC #CRL-3022): embryonic kidney from a female human fetus<br>HepG2 cells (ATCC #HB-8065): hepatocellular carcinoma from a male donor |
| Authentication | Cell lines were obtained from commercial suppliers and were therefore not authenticated. |
| Mycoplasma contamination | Cell lines were routinely tested negative for mycoplasma. |
| Commonly misidentified lines<br>(See ICLAC register) | none |

## Plants

| | |
|---|---|
| Seed stocks | N/A |
| Novel plant genotypes | N/A |
| Authentication | N/A |
