# Supplementary material for: Differential lipid selectivity of StARD phospholipid transporters revealed by native MS
Source: Nat Commun. 2026 Aug 22;17:10054. doi: 10.1038/s41467-026-76988-1 (PMC13594186; doi:10.1038/s41467-026-76988-1)
Supplement: Supplementary file 3 [file 41467_2026_76988_MOESM3_ESM.pdf]

nature portfolio

# Peer Review File

# Differential lipid selectivity of StARD phospholipid transporters revealed by native MS

Corresponding Author: Professor Carol Robinson

Parts of this Peer Review File have been redacted as indicated to remove third-party material.

**This file contains all reviewer reports in order by version, followed by all author rebuttals in order by version.**

Version 0:

Reviewer comments:

Reviewer #1

(Remarks to the Author)
In this manuscript, Kirschbaum and colleagues used high-end native mass spectrometry approaches to study the biochemical properties of three members of the STARD family of lipid transporters, STARD2, STARD7 and STARD10. They address several questions related to LTP functions: identify the native lipid cargoes, how gain or loss of transport function affects cellular lipidomes and the regulation of lipid transfer activities. The authors used a native MS-based approach to identify lipid co-purified with STARD2, STARD7 and STARD10 purified either from bacteria or human cells lines, at acyl chain resolution. By comparing these results to the total lipidome, they measured selectivity of the 3 STARDs for a specific acyl chain composition. They propose that the three STARDs studied have distinct selectivity for the acyl chain. Using overexpression and knock-down experiments, they suggest a link between these specificities and lipid remodeling in the total lipidome. Finally, they have identified the role of a conserved arginine for STARD7 and phosphorylation sites for STARD2 and STARD10 that are involved in PC transport.

The methods based on native MS are impressive. They should enable the characterization of STARD populations, e.g. apo and holo states. Establishing the link between phosphorylation and lipid occupancy would certainly be a technical tour the force. However, this part is not fully developed. The manuscript is full of interesting observations and, as mentioned, the approaches are truly powerful, but the scope remains poorly defined and there is a lack of clear messages to take away. I had to draw tables summarizing the multiple data (3 STARDs, in vitro, in HEK293, in HepG2, lipid bound, enrichment or not, phosphorylation, etc) scattered throughout the main and supplementary figures to understand the work. More problematic still, the quality of the main figures and their ability to support the claims and conclusions are limited. Much of the important data are currently buried in the 22 supplementary figures. The figure caption and, in general, the data analysis remains superficially described. In more details:

- Selectivity of the head group and acyl chain: It is reasonable to assume that similar results and conclusions would have been obtained using “classical” lipidomics based on LC-MS/MS. The authors should clearly explain the importance of using native MS for their purposes. If I understand correctly, this allows different STARD populations (assembled both in the tube and in the cell) to be disentangled/characterized: i.e., those that contain/do not contain a specific lipid, which is simply impossible to do with AP-LC-MS/MS approaches. It seems that this has not been sufficiently exploited.

- The selectivity profile they propose is somewhat limited and poorly supported by the main figures: STARD7 does not really bind to lipids (competition with acetate), STARD2 is not selective, while STRAD10 is selective for di-unsaturated lipids. Could it be that in another cell type STARD10 broadens its choice of lipids? Do MDS provide a hypothesis to explain this difference between STARD2 and STARD10?
The main figures do not provide convincing evidence for the claim regarding the selectivity profile. The enrichment in di-unsaturated fatty acids is only shown in vitro (Figure 1d). In cellulo, the data are hidden in supplementary figures S6, S7, and S8. The comparison between the histograms in Figure 2 and the enrichment/depletion analysis of the Sup data (S6, 7, 8) is not intuitive. The text does not provide a clear understanding of what was done and how to interpret it. The terms “selectivity/depletion” seems to be used without any threshold or statistics to support this claim. As this is an important claim, the data supporting it should be clearly presented (including statistics) in the main Figures 1 and 2.

- The message concerning STARD7 is confusing. On the one hand, the authors claim that this protein binds to lipids only very weakly in their assay, because the binding site is occupied by a competing acetate. They also provide structural evidence to support this claim. But then they claim that STARD7 has a different specificity than STARD2 and STARD10 for certain specific acyl chains. If the binding of STARD7 to acetate (and competition with lipids) is an artifact (due to the presence of acetate in their buffer system), I'm not sure that we can draw many conclusions from these experiments. But can they really exclude the possibility that association with acetate does not (also) occur in the cell? If this is the case, then this is a very interesting finding that warrants further experimentation (see below). Without additional experiments/evidence, the results obtained with STARD7 concerning lipid binding and specificity are not very convincing, and should not be so prominent.

- Acetate binding: it appears that acetate bound to STARD7 can be rapidly replaced by exogenously added isotopically labeled acetate, suggesting that this may indeed be an artifact. But could STARD7 also bind acetate in the cell and provide a regulatory mechanism? Acetate de novo synthesis in cells has been reported (10.1016/j.cell.2018.08.040). It is a precursor for acetyl-coenzyme A (Ac-CoA) metabolism and lipogenesis. It would be interesting to perform native MS experiments in an acetate-free buffer to test this hypothesis. I understand that this may not be entirely trivial, but it is probably worth the effort. In the transfer assay, was STARD7 bound to acetate and does the purification protocol imply the use of acetate (I guess not)? If acetate is bound, then is has to come from E. coli. Does acetate affect the transfer of PC in vitro. This should be feasible?

- Effect of overexpression: “Lipid extracts of cells overexpressing STARD2 or STARD10 contained higher amounts of unsaturated PC species than cells overexpressing STARD7, which synthesized mainly monounsaturated PC at similar abundances to wild type cells (Figure S15).” How do the authors explain this? STARD2 and STARD10 have either no selectivity or selectivity for di-unsaturated lipids, respectively. “Overexpression of individual LTPs resulted in a reproducible increase in their preferred PC species across both cell lines, highlighting a link between LTP expression levels and cellular lipid profiles. ” Confusing as you said something else in the first sentence (higher amounts of unsaturated PC). In addition, this does not seem to be true for STARD7 which overexpression does not modify PC species levels of the corresponding acyl chains. The remodeling is interesting, but not well carved out. What is the hypothesis, how would a gain in specific STARD function lead to this remodeling?

- PTMs: is certainly an intriguing part. But it is difficult to understand. Which form of STARD2 is phosphorylated, the apo or the holo? The statement “The measured mass of apo STARD2 was 42 Da higher than the expected mass, and we observed a peak shifted by 80 Da relative 278 to the apo protein, suggesting partial phosphorylation (ca. 20 %) (Figure 2b).” is very unclear. Can the authors relate the PTMs to a specific lipid-bound state?

General comments:
- There is a general lack of benchmarks and quality check: Are the STARDs folded and functional? A simple explanation would be that recombinant STARD7 is not functional. Could you measure folding or extract this information from the native MS experiments? Page 7 lines 133-134: “The native mass spectrum of STARD2 showed that >95 % of the protein copurified with phospholipids, suggesting stronger binding to human than to E. coli 134 lipids (70 %)”. An alternative explanation is that a larger fraction of STARD2 is folded in cellulo than in E. coli.
Similarly, in the biochemical transfer assay, was the quality of the liposome assessed (lipidomics, DSL, etc)?

- The data analysis is inconsistent. There is little mention of statistics and reproducibility. The graphs in figures 1 and 2 are without axis nor units. We do not know what is being measured nor what the values are. The legends of the figures fail not explain the units of the axis (e.g. %, percentage of what?).

- The figures have been overly simplified and lack important information: the Y axis labels, the number of replicates, and the statistical test used are missing. Some colours (i.e. PC, PE, PG) are not explained. The visual appearance of the graphs and the legends mainly constitute a conclusion of the result rather than a description of the data. It is therefore difficult to properly follow what has been done without consulting the materials and methods section very often.

- Insufficient reference to supplementary figures: the authors often fail to mention which panel of the supplementary figure is being cited, for example on page 8, line 184.

Specific points:
Figure 1: It is important to mention that the assay involves the use of detergent and that lipid uptake in this case involves the use of micelles (not membrane bilayers). The arrows between the gray boxes are misleading, as they give the impression of a sub-analysis. Figures 1d and S2 (lower panels) are identical.
Figure 2b, c: What are the y-axis of the histograms, is it pmol ? mol%? What are the error bars, from Methods, it seems that it is 3 recordings of the same sample in the MS.
Figure 3: 3a) The triangles indicated on top of the bars are not clear – what do they represent - significance?
Figure 6: For these experiments, several important information is missing and should be provided in the legend. Indeed, Fig 6b shows different curve profile than Fig. 6c, why? Could this be explained by the experimental setting? Are different amount of liposomes or protein used? What is the origin of the proteins used for each experiment: purified from bacterial or from HEK? It should be clearly stated in the figure legend.
In the text, the authors are making statements about differences and similarities of initial transfer rates. However, no quantification of this specific parameter can be found. What are the initial transfer rates, how reproducible are they? And are the differences statistically significant? Without this information it is difficult to draw any conclusion.
Along those lines, the authors describe some curves as “similar” (Fig6c: R->Q mutations of STARD2 and STARD10 only

minimally affect the transfer) and others as "different" (Fig6b: PC 16:0-12:0 and PC 18:1-12:0 are differently transferred by STARD2 and STARD10). However, simple comparison of the graph is not sufficient to make such statements. Real kinetic parameter/values should be extracted and compared. Statistical significance should be provided.
Line 114 : "from STARD10-expressing cells bacteria" should be "from STARD10-expressing bacteria"
Lines 133-134: "We characterized these ligands using multistage native MS and found that they were exclusively PC lipids". Why don't they see the other lipids they saw in vitro?
Line 207 : "The cellular lipidomes only exhibited modest changes in PC species, with the most prominent differences observed between the control and the three knockdown conditions (Figure S19)". Not clear what are these differences in S19.

Reviewer #2

(Remarks to the Author)
I co-reviewed this manuscript with one of the reviewers who provided the listed reports. This is part of the Nature Communications initiative to facilitate training in peer review and to provide appropriate recognition for Early Career Researchers who co-review manuscripts.

Reviewer #3

(Remarks to the Author)
Kirschbaum and colleagues present an interesting study elucidating the lipid selectivity profiles of three phospholipid transport proteins STARD2, STARD7 and STARD10. They utilise a multistage native mass spectrometry (MS) pipeline as the centrepiece of this work, which is complemented by molecular dynamics simulations, lipidomics, mutagenesis, and in vitro transport assays. Overall, the study is thorough, rigorous, and relatively well presented. I believe it is suitable for publication after the authors address my minor comments below.
- The presence of an acetate adduct on STARD7 is an obscure observation. The authors may wish to provide further clarification as to the origin of such an adduct and the reason for it.
- The authors state that acetate may have displaced co-purified lipids in STARD7. Are lipids observed prior to buffer exchange into ammonium acetate, for example by TLC?
- Could the authors provide an additional discussion of why STARD7 binds lipids with such low affinity in vitro?
- The purity of STARD7 appears lower in both E. coli and mammalian cell expression systems. Are the higher molecular weight bands in the SDS-PAGE gels oligomeric species or contaminants? Could high contaminant levels be impacting co-purification of lipids?
- It is stated that STARD7 is truncated – it would be helpful to indicate full length and truncated products in the intact spectra/zoomed insets.
- It is stated that Fig 3 displays how expression of the three proteins alters lipid compositions. I can not interpret Fig 3a as drawn. Perhaps some labels are missing? What are the arrows? What are the statistical tests used (I presume that's what the *s denote). What 'relative quantitation' was performed. Presumably to a non-expressing control? More details and clarity are needed.
- Figure 3b is repeated in Supp Figure S20. I would suggest removing this from Figure 3 (see next comment).
- The statements around tissue specific protein/lipid distributions are intriguing but underdeveloped and lack rigorous support. The authors conclusively draw a correlation by only considering two datapoints (liver and heart). Are additional data available of tissue specific lipid profiles to provide further evidence to support the authors claims? If not, the authors should tone down their statements about this data. Moreover, such expression data are from transcriptomics, which does not always correlate with protein levels. This caveat must be stated.
- Did the authors examine lipid binding in their phosphatase treated proteins? This might help to provide additional clarity to the interpretation of lipid transfer functional data.
- I found it difficult to navigate the supplementary. Whilst I understand the data are thematically organised, it would be better for the reader if this was sequentially arranged.

Reviewer #4

(Remarks to the Author)
The authors have used native mass spectrometry to investigate the lipid selectivity of three lipid transfer proteins: STARD2, STARD7 and STARD10. They report different lipid preferences for the three proteins and in particular their selectivity towards acyl chains: STARD2 copurifies with long polyunsaturated chains, STARD7 binds a wide range of lipids, and STARD10 copurifies preferentially with di-unsaturated phospholipids. This is also observed in in vitro lipid transfer assay. This work brings new and important information on the elusive acyl chain selectivity of three lipid transfer proteins and is in principle of interest for the readership of Nature Communications.

Unfortunately it does not succeed at proposing a structural explanation, despite extensive molecular dynamics simulations. For example a limitation of the simulations is that DOPC was used for all three proteins, despite the experiments showing that they have different acyl chain selectivity.

The molecular dynamics simulations were used to monitor the dynamics of a docked DOPC in the pockets of STARD2, 7 and 10 wild-type and of an arginine to glutamine mutant. The arginine is conserved and has been observed before to interact with a bound lipid in STARD2 (Titeca et al, Nature, 2026). The lipid bound to STARD7 is observed to be more mobile than the lipids bound to STARD2 and STARD10. The authors conclude that their simulation data "suggest that

STARD7 has lower affinity for PC lipids compared to STARD2 and STARD10". The statement "has lower affinity for PC lipids" is not correct given the data shown and should be replaced by "engages in weaker interactions with DOPC compared to STARD2 and STARD10".

The starting structures are built with Boltz-2 (a co-folding method) and shown on Figure 4C. MD simulation outcomes are in general dependent on input structures and the authors do not sufficiently document and discuss the differences between the DOPC-bound models of STARD2, 10 and 7 might and how these might have influenced the differential behavior of STARD7. The authors should provide information about the difference between their 3 models obtained with Boltz-2 and the lipid-protein complexes of STARD2, and the differences between the three models. One cannot see from Figure 4c if the three lipids have comparable poses, or if their headgroup is positioned in the cage as expected from the 3 STARD2 X-ray structures. The best would be to provide the three models as pdb or mmcif files as supporting information.

The RMSF (Figure 4d) of the DOPC lipid headgroup is fairly high for STARD7. Interestingly STARD2 and STARD7 have a conserved aromatic cage but the cage is absent from STARD10. One would therefore expect that the DOPC headgroup would be more loosely bound in STARD10 than in 2 or 7, but it does not seem to be the case. Is that correct? Please provide distances between the choline group and the aromatics of the cage.

Version 1:

Reviewer comments:

Reviewer #1

(Remarks to the Author)
The authors have carefully addressed our requests. The manuscript has improved in terms of readability. It is now much easier to navigate the complex data and the importance/relevance of the results are clearly demarcated. We find the approach interesting and powerful. The authors demonstrate how native spectrometry approaches can contribute to the understanding of LTP functioning in the cell. This should be of interest to a large community. As the experiments are complex and it may help the reader to further clarify a few points:
- To avoid any confusion, it is best to use only the term "mitochondrial localisation signal" to refer to the 1-74 sequence of STARD7 (rather than the term "signal peptide").
- As mentioned the experiments are complex and Figure 1a,b,c could benefit from a rework to improve readability and understanding.
On the left panels:
i) we barely see the grey box around the selected m/z and it takes quite some time to link it to the grey insets. As this is an important figure it may deserve a larger size.
ii) the grey insets differ in the presentation between the 3 proteins: the 9+, 11+ and 12+ are not always written at the same place, which is confusing. The position versus the x-axis (and explanation) of the schematic representation of the LTP (coloured (red/green/violet) cheese), is difficult to grasp. This should be clarified and/or explained in the legend.
On the right panels:
iii) These panels represent different MS experiments than the ones presented on the left – and this is not obvious from the display. They imply the use of micelles solubilised by C8E4 to determine lipid class selectivity and this is still not evident from the display, i.e. without the legend. A scheme or text could be added in the figure to make it clearer. Or may be better, a schematic of the 2 workflows explaining the principle of these experiments would help a lot the non-expert.
- Figure 2b. It is not clear to what lipids the R Q mutant proteins were exposed to. From the available information in the legend and main text, it seems that STARD2 and STARD10 data does not correspond to the same experiment as for STARD7. The text says STARD7 R189Q mutant is not binding to PC even when incubated with a large excess of DOPC - is it what is shown in panel 2b? However, the legend suggests that the MS measurements are from STARD7 exposed to bacterial lipids during expression. This requires clarification.
- Since STARD7 WT does not bind so much DOPC (Fig1b, right panel), a comparison of the difference of PC binding between STARD7 WT and R182Q in the same plot would strengthen the statement that STARD7 R182Q has impaired PC binding. At the moment, this is mostly supported by the loss of acetate binding. You can use the data of figure S5b.
- The description of STARD10 phosphorylation is intriguing. On the one hand, 95% of STARD10 from HEK and HepG2 contains lipids and STARD10 is highly phosphorylated ("95 and 90% at H229-R272 and M276-H322, respectively"). On the other hand, the in vitro transfer assay shows reduced transfer of phosphorylated STARD10 compared to dephosphorylated, suggesting that phosphorylation prevents transfer. How do you explain that? Is it possible that in cells, phosphorylated STARD10 is stalled in a lipid-bound state that prevents release which would explain the high amount of holo-STARD10? Can native MS help figure out these differences? Also, testing what step of lipid transfer is affected by phosphorylation would be interesting. Is it the uptake? the release? The binding to membranes? We realize that this may not be trivial, but this should at least be discussed.
- Figure 7 : The model is interesting but is currently not in total accordance with the data presented here. The suggestion that phosphorylation affect binding to membrane and lipid transfer requires confirmation/clarification for STARD10 (see comment above – and then STARD10 should be represented with a lipid in the binding pocket) and is not supported for STARD2 nor STARD7. The actual version of the manuscript does not allow to claim for a general role of phosphorylation to prevent lipid uptake/transfer by these three LTPs.
- On several occasion, the authors use the term "affinity" to describe in reference to the proportion of STARD that forms complex with a cargo. The authors did not measure affinities. In this context, affinity is likely not the only determining factor for the observed occupancy rate. STARD proteins must have an affinity for their cargo in order to remove them from

membranes, but for the transfer to occur, they must then lose affinity for them to release the lipid in the acceptor membrane. The observed occupancy rate could be explained by many ways: higher uptake, lower release, etc (see comment above on STARD10). "Affinity" should be rephrased.

Typo :
Line 2 page 6 : check bracket positioning.
Figure 6 panel E : Write which STARD is analysed (STARD10).
Page 12, line 23 : Pay attention to "(Figure 4a)" position in the sentence.

Reviewer #2

(Remarks to the Author)
I co-reviewed this manuscript with one of the reviewers who provided the listed reports. This is part of the Nature Communications initiative to facilitate training in peer review and to provide appropriate recognition for Early Career Researchers who co-review manuscripts.

Reviewer #3

(Remarks to the Author)
The authors have comprehensively addressed all my comments in this revised manuscript, with significant new data included. The manuscript is certainly ready for publication in its present form.

Reviewer #4

(Remarks to the Author)
I wish to thank the authors for their answers and the additional data. Overall I find that, unfortunately, they have not provided enough controls and/or data to rule out the effect of their starting structures on the outcome of the simulations, thus weakening the significance of their conclusions. Since the simulations are available, I believe that it should not take much time for the authors to provide answers to my questions below.
My overall impression is that the simulation data is not critical to explain or support the experimental findings which appear convincing enough. The authors should nonetheless avoid over-interpreting simulation data, which, as I see it, do not yet convincingly explain why the R to Q mutation only has an effect on STARD7.

(1) From the authors: # Our choice to select DOPC as the ligand to simulate with all three proteins was driven by two main rationales. First, it is a common ligand for all three proteins. Second, using a single ligand enables direct comparison of interaction profiles across the different proteins. We have now carried out comparable additional simulations with the respective most abundant acyl chain substrate for each protein (SAPC for STARD2 and POPC for STARD7, results are shown in Figure S6d and Figure S6e) and confirmed our initial qualitative results that STARD7 engages its natural substrate with weaker interactions compared to STARD2 and STARD10.#

I thank the authors for their answer and appreciate the addition of simulations of STARD2 with SAPC, a polyunsaturated lipid, and of STARD7 with POPC (monounsaturated lipid). Comparing Figure 2d and Supplementary Figure S6, it does not seem like SAPC and DOPC exhibit different mobilities in simulations with STARD2. It is unlike the experimental data showing that STARD2 preferentially binds to polyunsaturated lipids, and is actually a very interesting observation indicating that the STARD2 pocket might actually be able to host a larger variety of lipids than those observed experimentally. Would the authors comment on this?

(2) From the authors: # The predicted models, solvated and ionized, are already available in the supporting raw files under the name "initial.pdb" for each protein under the corresponding filename. Further, we now added a Supplementary Figure (Figure S6a, also attached below), to better describe the binding pose of DOPC in all models with reference to the crystal structure of lipid-bound STARD2 (PDB-ID: 1LN1).#

I appreciate that the authors provide all raw files and apologize if I missed them previously. In the Boltz-2 model for STARD10 with DOPC, the choline group is placed at a completely different site than for STARD2 and STARD7, where it naturally falls within the aromatic cage. STARD10 retains one of the four aromatic amino acids of the cage (Tyr117). Have the authors considered the possibility that the choline could engage in cation-pi interactions with that tyrosine? Have different starting structures been tested?

In the starting structure for the simulations of STARD7 with DOPC (the only lipid for which I scrutinized the MD files) there is no hydrogen bond between the conserved arginine and the phosphate (also visible on Figure 2e). Can the authors rule out that this could be a cause to the lower occupancy of the phosphate-arginine weaker hydrogen bond (and weaker interactions with the aromatic cage)? What would happen if the lipid was positioned similarly to that starting structure of STARD2? As far as I understand from the Methods section, the replicates for each MD unfortunately use the same starting structure.

(3) From the authors: # The attached figure below shows the aromatic cages of STARD2 and STARD7, showing comparable
binding of the aromatic cage to the lipid headgroup in the two proteins.

Analysis of the individual distances between the centre of mass of the choline group and the centre of mass of the aromatic cage are now shown in Figure S6b (also attached below). Interestingly, these data show that the choline group exhibits comparable binding to the aromatic cage in both wildtype STARD2 and STARD7. Upon mutation of the conserved arginine residue into glutamine, the binding of the choline group appears more weakened in STARD7 than in STARD2.#

The authors seem to have misunderstood what analysis I asked about, namely the distance between the choline group (eg N atom, but COM of choline is ok) and each of the aromatic groups of the cage. The distance to the COM of the cage does not reflect if the cage remains in its expected structure around the choline, as observed in Xray structures of STARD2. Related to this question, the authors declare using the CHARMM force field but do not mention if they used the WYF correction for choline-aromatics cation-pi interactions. If this correction (or any equivalent) has not been applied, it is important to provide time series of either the cation-pi occupancies or of the choline-aromatics distances.
With the provided data, I see no support for the following sentences: “Interestingly, the choline group exhibits comparable binding to the aromatic cage in STARD2 and STARD7 (Figure S6b). Upon mutation of the conserved arginine residue into glutamine, however, the binding of the choline group appeared more weakened in STARD7 than in STARD2.”

Version 2:

Reviewer comments:

Reviewer #1

(Remarks to the Author)
The authors have addressed all conerns raised. We find this research interesting. It demonstrates how native spectrometry techniques can contribute to our understanding of how LTP functions within the cell, which should be of interest to a wide audience. We recommend its publication.

Reviewer #2

(Remarks to the Author)
I co-reviewed this manuscript with one of the reviewers who provided the listed reports. This is part of the Nature Communications initiative to facilitate training in peer review and to provide appropriate recognition for Early Career Researchers who co-review manuscripts.

Reviewer #4

(Remarks to the Author)
The authors have satisfactorily answered most of my questions and comments. Yet, in one case (position of lipid in STARD10), the corresponding statements in the manuscript do not reflect the results presented, nor the answers that the authors provided in the rebuttal.

I acknowledge that the choice of the model for lipid-bound STARD10 is supported by several prediction tools. This model significantly differs from lipid-bound structures of STARD2 (eg. 1LN1 pdb id) and from the model for STARD7. The authors also confirmed in the rebuttal that the lipid choline headgroup in STARD10 is not positioned at the same place as in STARD2/7. All of this is fine but I suggest that the authors state the difference clearly in the text and correct the sentence starting at line 180. As it stands now, the text is at best misleading:
“As starting models for the simulations, we first obtained structural models for DOPC-bound STARD2, STARD7(76–370), and STARD10, using Boltz-240 (Figure 2c). The predicted binding poses are consistent with the location and orientation of DOPC in the crystal structure of STARD2 (Figure S6a), ...”

Likewise, the sentence starting l.188 needs to be corrected as it reads as a statement valid for the three proteins given the sentence prior to it:
“Similarly, the interaction of the DOPC headgroup with aromatic residues inside the binding cavity remained stable throughout the simulations (Figure S7a,b)."
This statement is only valid for STARD2 and STARD7.

Finally, and related to the above:
contrary to what is stated in the main text and in the rebuttal, the data on Fig.S7a does not support a cation-pi interaction between Tyr117 in STARD10 and the lipid choline headgroup. The snapshot does not indicate that the cation is stacked on either side of the ring, and the minimum distance between the (whole?) residue and the choline nitrogen seems like the wrong readout. The structure of cation-pi interactions is fairly basic structural biology (see for example 10.1126/science.271.5246.163, or Minoux and Chipot, JACS, 1999). To my eyes it actually looks as if there is no cation-pi interactions between the two groups in STARD10.

Please correct the text.

**Open Access** This Peer Review File is licensed under a Creative Commons Attribution 4.0 International License, which permits use, sharing, adaptation, distribution and reproduction in any medium or format, as long as you give appropriate credit to the original author(s) and the source, provide a link to the Creative Commons license, and indicate if changes were made.
In cases where reviewers are anonymous, credit should be given to 'Anonymous Referee' and the source.
The images or other third party material in this Peer Review File are included in the article's Creative Commons license, unless indicated otherwise in a credit line to the material. If material is not included in the article's Creative Commons license and your intended use is not permitted by statutory regulation or exceeds the permitted use, you will need to obtain permission directly from the copyright holder.
To view a copy of this license, visit https://creativecommons.org/licenses/by/4.0/

Reviewer's Comments:

**Reviewer #1 (Remarks to the Author)**

In this manuscript, Kirschbaum and colleagues used high-end native mass spectrometry approaches to study the biochemical properties of three members of the STARD family of lipid transporters, STARD2, STARD7 and STARD10. They address several questions related to LTP functions: identify the native lipid cargoes, how gain or loss of transport function affects cellular lipidomes and the regulation of lipid transfer activities. The authors used a native MS-based approach to identify lipid copurified with STARD2, STARD7 and STARD10 purified either from bacteria or human cells lines, at acyl chain resolution. By comparing these results to the total lipidome, they measured selectivity of the 3 STARDs for a specific acyl chain composition. They propose that the three STARDs studied have distinct selectivity for the acyl chain. Using overexpression and knock-down experiments, they suggest a link between these specificities and lipid remodeling in the total lipidome. Finally, they have identified the role of a conserved arginine for STARD7 and phosphorylation sites for STARD2 and STARD10 that are involved in PC transport.

The methods based on native MS are impressive. They should enable the characterization of STARD populations, e.g. apo and holo states. Establishing the link between phosphorylation and lipid occupancy would certainly be a technical tour the force. However, this part is not fully developed. The manuscript is full of interesting observations and, as mentioned, the approaches are truly powerful, but the scope remains poorly defined and there is a lack of clear messages to take away. I had to draw tables summarizing the multiple data (3 STARDs, in vitro, in HEK293, in HepG2, lipid bound, enrichment or not, phosphorylation, etc) scattered throughout the main and supplementary figures to understand the work. More problematic still, the quality of the main figures and their ability to support the claims and conclusions are limited. Much of the important data are currently buried in the 22 supplementary figures. The figure caption and, in general, the data analysis remains superficially described. In more details:

# We thank the reviewer for the thorough and rigorous evaluation of our manuscript. In response, we have streamlined the overall flow, clarified the scope, and reorganized the presentation to improve clarity. Specifically, we now present the results in a logical progression: STARD proteins recombinantly expressed in *E. coli,* followed by expression in HEK293S cells, then in HepG2 cells, and finally the transport activity analyses. We have also restructured the Supporting Information to improve navigability, arranging the figures according to their order of appearance in the main text rather than by topic. Individual points are addressed in more detail below.#

- Selectivity of the head group and acyl chain: It is reasonable to assume that similar results and conclusions would have been obtained using "classical" lipidomics based on LC-MS/MS. The authors should clearly explain the importance of using native MS for their purposes. If I understand correctly, this allows different STARD populations (assembled both in the tube and in the cell) to be disentangled/characterized: i.e., those that contain/do not contain a specific lipid, which is simply impossible to do with AP-LC-MS/MS approaches. It seems that this has not been sufficiently exploited.

# We agree that the advantages of native MS over LC-MS/MS for the identification of protein-bound lipids were not sufficiently explained in the previous version of the manuscript. In our view, native MS offers two major advantages:

1. As correctly pointed out by the reviewer, native MS enables quantitative characterization of intact protein-lipid complexes. This includes the quantification of apo versus lipid-bound protein species, as well as determination of the protein:lipid binding stoichiometry. This information cannot be obtained by LC-MS/MS analysis of extracted lipids, as the binding stoichiometry is lost during extraction.

2. Native MS provides superior selectivity. By isolating intact protein-lipid complexes in the gas phase, prior to releasing the bound lipids, we can ensure that the detected lipids were originally bound to the

protein. In contrast, LC-MS/MS is performed on lipid extracts obtained after protein purification. Even in the case of soluble LTPs, those lipid extracts could contain contaminant lipids that are not specifically bound in the lipid binding pocket, particularly highly abundant species such as phosphatidylcholine.

To assess specific binding using LC-MS/MS, lipid extracts from purified proteins must therefore be compared to membrane lipid extracts to identify enrichment of particular lipid species (see for example DOI: 10.1126/science.aah6171). Alternatively, a recently published workflow analyzes lipids after size-exclusion chromatography to capture only lipids that co-elute with the protein (DOI: 10.1038/s41586-025-10040-y). While this approach improves selectivity, it also involves substantially greater experimental effort and does not guarantee that the lipids are engaged in a specific complex. We have revised the discussion section of the manuscript to outline more clearly the advantages of native MS over LC-MS/MS:

Page 20:

Compared to classical approaches based on affinity purification and identification of bound lipids by separate LC-MS/MS analysis, our method offers two major advantages. First, native MS enables quantitative characterization of intact protein–lipid complexes, including protein:lipid binding stoichiometry and the relative abundance of *apo* versus lipid-bound protein. These data provide biophysical insights that manifest in functional differences. For example, the lower degree of lipid binding to STARD7 observed by native MS correlates with its reduced rate of lipid transfer in liposome assays. Second, isolation of intact protein–lipid complexes in the gas phase prior to controlled lipid release ensures that detected lipids were initially bound to the protein as specific complexes, whereas contaminant lipids are effectively excluded from the analysis. Our approach thus eliminates the need for additional lipid extraction or chromatographic separation steps while enabling deep structural analysis of lipids that selectively bind to LTPs, including their acyl chain composition.#

- The selectivity profile they propose is somewhat limited and poorly supported by the main figures: STARD7 does not really bind to lipids (competition with acetate), STARD2 is not selective, while STRAD10 is selective for di-unsaturated lipids. Could it be that in another cell type STARD10 broadens its choice of lipids? Do MDS provide a hypothesis to explain this difference between STARD2 and STARD10?
The main figures do not provide convincing evidence for the claim regarding the selectivity profile. The enrichment in di-unsaturated fatty acids is only shown in vitro (Figure 1d). In cellulo, the data are hidden in supplementary figures S6, S7, and S8. The comparison between the histograms in Figure 2 and the enrichment/depletion analysis of the Sup data (S6, 7, 8) is not intuitive. The text does not provide a clear understanding of what was done and how to interpret it. The terms "selectivity/depletion" seems to be used without any threshold or statistics to support this claim. As this is an important claim, the data supporting it should be clearly presented (including statistics) in the main Figures 1 and 2.

# We have revised Figures 1 and 2 (now Figure 3) to improve clarity and enhance the quantitative presentation of the selectivity profiles. In Figure 1, the correlation plots compare the relative abundance of individual PE and PG species bound to STARD2 or STARD10 with their respective abundances in whole-cell lipid extracts from the corresponding cell pellets. The figure legend has been expanded to more clearly describe the plots and the associated statistical analyses.

For Figure 3, we adopted a different visualization strategy to better represent the greater complexity of human PC lipid species (correlation plots in the Supporting Information). Figure 3d now presents scatter plots illustrating enrichment and depletion of individual PC species. Enrichment and depletion are color-coded (blue = enrichment; red = depletion), while the magnitude of the difference is represented by circle area. The color intensity encodes adjusted p-values derived from biological triplicates using two-sided Welch's t-tests and corrected for multiple hypothesis testing using the

Benjamini–Hochberg false discovery rate (FDR) procedure. We only show this analysis for the HepG2 dataset, as the comparatively restricted lipid repertoire of HEK293S cells does not permit robust, generalizable conclusions about acyl chain selectivities. We now explicitly state that this consideration motivated the additional expression of the proteins in HepG2 cells.

Page 11:

However, the lipid repertoire of HEK293S cells is relatively restricted and largely dominated by monounsaturated PC species,[41] which may limit the generalizability of our results. To investigate acyl chain selectivity in a context of broader lipid diversity, we characterized StARD cargo in HepG2 (hepatocellular carcinoma) cells, which possess a richer lipid composition, notably including polyunsaturated PCs.

In this regard, the reviewer raises an important point: preferred ligands can only be identified in expression systems that actually synthesize the respective lipid species. For example, a previous study investigating the ligand preference of STARD10 detected a preference for phospholipids with one saturated lipid chain, simply because lipids with two unsaturated chains were not present in the lipid extract used in that assay (DOI: 10.1074/jbc.M413330200). In this context, HepG2 cells provide the most suitable model system, as they produce PC species spanning the full range from mono- to polyunsaturated acyl chains. We therefore do not believe that STARD10 would bind a broader lipid pool under different conditions, as the relevant lipid diversity is already represented in the HepG2 system.

In summary, our data indicate that STARD7 does not exhibit pronounced acyl chain selectivity, based on the lipid species copurified with STARD7 expressed in human cells. When expressed in bacteria, STARD7 does not copurify with detectable lipids. We attribute this not to competition with acetate, but rather to a low binding affinity for bacterial PE and PG species (see below). For STARD2, we observe a preference for polyunsaturated lipids. However, this selectivity becomes apparent only when the protein is purified from HepG2 cells, as neither *E. coli* nor HEK293S cells produce substantial levels of polyunsaturated phospholipids, thereby limiting the accessible lipid pool. In contrast, STARD10 consistently enriches di-unsaturated lipid species across all cell lines examined, indicating robust intrinsic acyl chain selectivity. Unfortunately, MD simulations cannot provide an explanation for the observed difference between STARD2 and STARD10. This would require plenty of extensive free energy calculations, but even those would be strongly biased by the initial configuration obtained from Boltz-2.#

- The message concerning STARD7 is confusing. On the one hand, the authors claim that this protein binds to lipids only very weakly in their assay, because the binding site is occupied by a competing acetate. They also provide structural evidence to support this claim. But then they claim that STARD7 has a different specificity than STARD2 and STARD10 for certain specific acyl chains. If the binding of STARD7 to acetate (and competition with lipids) is an artifact (due to the presence of acetate in their buffer system), I'm not sure that we can draw many conclusions from these experiments. But can they really exclude the possibility that association with acetate does not (also) occur in the cell? If this is the case, then this is a very interesting finding that warrants further experimentation (see below). Without additional experiments/evidence, the results obtained with STARD7 concerning lipid binding and specificity are not very convincing, and should not be so prominent.

# We acknowledge that the presentation of the STARD7 data may have caused confusion. In the revised manuscript, we clarify the following points: STARD7 copurifies with fewer lipids than STARD2 and STARD10, as reflected by a lower lipid-bound:apo protein ratio in the native mass spectra (Figure 3a). Importantly, this observation is not due to competition with acetate in the native MS buffer, contrary to our previous hypothesis. Rather, STARD7 intrinsically copurifies with fewer lipids, and acetate from the native MS buffer fills up vacant lipid binding sites while not displacing bound lipids

(see next point). No PE or PG species copurified with STARD7 when the protein was expressed in *E. coli* (Figure 1b), precluding any conclusions regarding its acyl chain selectivity in Figure 1.

In contrast, when expressed in human cells, STARD7 co-purified with a substantial fraction of PC species, enabling assessment of its acyl chain selectivity in Figure 3d. This again demonstrates the value of native MS in these types of study. Without quantitative readouts of the % protein bound to lipid, LC-MS/MS workflows would not uncover the lower apparent affinity. All aspects related to acetate binding are addressed collectively in the following point.#

- Acetate binding: it appears that acetate bound to STARD7 can be rapidly replaced by exogenously added isotopically labeled acetate, suggesting that this may indeed be an artifact. But could STARD7 also bind acetate in the cell and provide a regulatory mechanism? Acetate de novo synthesis in cells has been reported (10.1016/j.cell.2018.08.040). It is a precursor for acetyl-coenzyme A (Ac-CoA) metabolism and lipogenesis. It would be interesting to perform native MS experiments in an acetate-free buffer to test this hypothesis. I understand that this may not be entirely trivial, but it is probably worth the effort. In the transfer assay, was STARD7 bound to acetate and does the purification protocol imply the use of acetate (I guess not)? If acetate is bound, then is has to come from E. coli. Does acetate affect the transfer of PC in vitro. This should be feasible?

# We performed further native MS experiments on STARD7 in acetate-free buffers and have included these results in the revised manuscript (**Figure S3**). Specifically, we tested ammonium formate and ammonium bicarbonate, two other native MS compatible buffers. In ammonium formate, STARD7 forms 1:1 complexes with formate, analogous to the acetate adduct observed previously. In ammonium bicarbonate, multiple bicarbonate adducts are detected, requiring in-source activation to obtain an interpretable mass spectrum. Such activation is not ideal, as it may also lead to dissociation of bound lipids. We also conducted additional titration experiments using STARD7 expressed in *E. coli* to reduce the ammonium acetate and ammonium formate concentrations down to 25 mM and 10 mM, respectively (200 mM being the standard concentration used throughout the manuscript). Further reduction significantly affected the quality of the mass spectrum. We observed a concentration-dependent decrease in both acetate and formate adduct formation. The concentration-dependent reduction of acetate adducts suggests that no substantial amount of acetate is carried over from the cells. However, this does not constitute definitive proof, as intracellular acetate concentrations are expected to be lower than those used in our buffer system and we do not expect to detect endogenous acetate adducts by native MS. We would like to emphasize here that binding of volatile molecules such as acetate is generally not observed by native MS, and the fact that we observe well-defined 1:1 adducts with acetate could have a biological meaning, though the acetate adduct observed in our experiment originates from the native MS buffer.

While reduced ammonium acetate concentrations led to a reduction of acetate adducts, this did not lead to concomitant appearance of lipid-bound species. This indicates that acetate does not compete with bound lipids. Rather, these results support the conclusion that STARD7 expressed in bacteria does not copurify with detectable lipids. We confirmed this by LC-MS/MS analysis of copurified lipids in the absence of acetate (see request by Reviewer 3) (**Figure S4**) and corrected our conclusions accordingly.

Page 5:

We tested alternative buffers to obtain a mass spectrum in the absence of acetate. Ammonium formate formed analogus 1:1 formate adducts with STARD7, while ammonium bicarbonate produced nonspecific adducts (**Figure S3d,e**). Lipid binding was not observed under any condition tested. We therefore performed LC-MS/MS lipidomics on purified STARD2, STARD7, and STARD10 to quantify copurified lipids prior to buffer exchange. STARD7 copurified with an order of magnitude fewer phospholipids than STARD2 and STARD10 (**Figure S4**). We conclude that STARD7 has a relatively low affinity for phospholipids and is predominantly in its *apo* form when purified from *E. coli*. The absence of lipids allows carboxylates to enter the empty lipid binding pocket upon buffer exchange into native

MS buffer. As acetate binding is not typically observed by native MS, the detection of 1:1 complexes with acetate could have biological relevance. However, any endogenously bound carboxylates would likely be lost during purification and thus remain undetected.

In the lipid transfer assay, acetate was not present. The purification protocol for STARD7 does not involve acetate-containing buffers. All lipid transfer assays were performed in 20 mM Tris, 150 mM NaCl. We also conducted the lipid transfer assay in 200 mM ammonium acetate buffer, but did not include the data in the manuscript. Under these conditions, we did not observe an obvious reduction in the rate constant for lipid transfer by STARD7, whereas slight reductions were observed in the rate constants for STARD2 and STARD10. The maximum fluorescence intensity differed substantially between the two buffers, suggesting that NBD-PC fluorescence intensity is highly buffer-dependent.

Because acetate did not have a clear effect on STARD7 lipid transfer activity and we do not wish to further speculate on potential roles of acetate binding, we decided not to pursue this aspect further. As the transfer assays in ammonium acetate buffer were not performed in replicate, the data were not included in the manuscript and are provided here for review purposes only. The data points in the left graph represent mean values from triplicate measurements, whereas data points on the right correspond to single measurements. Curves represent the kinetic fit using a first-order approach. The rate constant for lipid transfer by STARD7 is not decreased in ammonium acetate buffer compared to acetate-free liposome buffer.

[editorial note: unpublished, confidential data redacted]

- Effect of overexpression: "Lipid extracts of cells overexpressing STARD2 or STARD10 contained higher amounts of unsaturated PC species than cells overexpressing STARD7, which synthesized mainly monounsaturated PC at similar abundances to wild type cells (Figure S15)." How do the authors explain this? STARD2 and STARD10 have either no selectivity or selectivity for di-unsaturated lipids, respectively. "Overexpression of individual LTPs resulted in a reproducible increase in their preferred PC species across both cell lines, highlighting a link between LTP expression levels and cellular lipid profiles. " Confusing as you said something else in the first sentence (higher amounts of unsaturated PC). In addition, this does not seem to be true for STARD7 which overexpression does not modify PC species levels of the corresponding acyl chains. The remodeling is interesting, but not well carved out. What is the hypothesis, how would a gain in specific STARD function lead to this remodeling?

# We have reformulated this entire section to clarify our arguments. In brief, based on the revised Figure 3d, we make the following claims regarding acyl chain selectivity:

1. STARD2 preferentially enriches PC species with an increasing degree of unsaturation, particularly polyunsaturated PC.

2. STARD10 preferentially enriches di-unsaturated species.

3. Both STARD2 and STARD10 markedly deplete monounsaturated PC species.

4. In contrast, the PC profile copurified with STARD7 closely resembles that of the expression host, indicating minimal acyl chain selectivity.

In lipid extracts from cells overexpressing STARD2, STARD7, or STARD10, monounsaturated lipid levels in STARD7-overexpressing cells were unchanged compared to wild-type cells. In contrast, overexpression of STARD2 or STARD10 increased the abundance of phospholipids with two or more double bonds, consistent with their binding preferences.

In the Discussion (Page 20/21; Figure 7), we carve out our hypothesis that these LTPs selectively extract specific PC species from the ER, thereby triggering compensatory resynthesis of these species through equilibrating lipid metabolism. For example, extraction of polyunsaturated PC would increase membrane rigidity, which in turn promotes resynthesis of polyunsaturated species to maintain membrane homeostasis.#

- PTMs: is certainly an intriguing part. But it is difficult to understand. Which form of STARD2 is phosphorylated, the apo or the holo? The statement "The measured mass of apo STARD2 was 42 Da higher than the expected mass, and we observed a peak shifted by 80 Da relative 278 to the apo protein, suggesting partial phosphorylation (ca. 20 %) (Figure 2b)." is very unclear. Can the authors relate the PTMs to a specific lipid-bound state?

# We agree that the original statements were difficult to follow and have revised the main text and Figure 5 to improve clarity. In brief, the degree of phosphorylation is indistinguishable between the apo and lipid-bound forms of the proteins. However, as more than 95% of STARD2 and STARD10 are lipid-bound, it is difficult to visualize phosphorylation on the apo proteins in Figure 3a due to the very low abundance of the apo peak. In the revised manuscript, we now state early in the description of Figure 3 that the lipid-binding pattern observed in the native mass spectra of STARD2 and STARD10 are uniformly shifted by 80 Da, consistent with phosphorylation

Page 9:

We further note that the lipid binding pattern is repeated with an 80 Da mass shift, suggesting partial phosphorylation of the protein, as discussed in more detail below.

This consistent mass shift of all lipid-bound states demonstrates that phosphorylation is not associated with any specific lipid-bound state. We have expanded the section on STARD2 phosphorylation to provide a more detailed explanation and improve clarity.

Page 14:

The measured mass of *apo* STARD2 expressed in HEK293S and HepG2 cells was 42 Da higher than the expected mass, which we assigned to N-terminal acetylation. In addition, we observed a peak shifted by 80 Da relative to the *apo* protein, and the lipid-bound peaks were likewise reproduced with the same 80 Da shift (**Figure 3a**). This pattern is consistent with partial phosphorylation of the protein (approximately 20%), as estimated from the relative peak intensities observed in the native mass spectrum. The uniform 80 Da shift across the *apo* protein and all lipid-bound states indicates that phosphorylation is not associated with any specific lipid-bound species.

In the section on phosphorylation sites, we have expanded the description of the data and experimental procedures to clarify the observed phosphorylation pattern of STARD2. We have also restructured Figure 5 so that the experimental results are presented in the same order as they appear in the text. Importantly, we now explicitly state that in-source activation was applied to acquire the native mass spectra shown in Figure 5. In-source activation removes bound lipids, reducing the spectrum to peaks corresponding exclusively to the apo protein and thereby facilitating relative quantification of the phosphorylated species.

Page 14:

To accurately quantify the phosphorylated species, subsequent native mass spectra were acquired with in-source activation to dissociate all bound lipids, reducing the spectrum to two predominant peaks corresponding to *apo* STARD2 and its phosphorylated form. Phosphorylation of STARD2 was confirmed by treatment with $\lambda$-phosphatase, which eliminated the +80 Da peak (**Figure 5a**).#

General comments:

- There is a general lack of benchmarks and quality check: Are the STARDs folded and functional? A simple explanation would be that recombinant STARD7 is not functional. Could you measure folding or extract this information from the native MS experiments? Page 7 lines 133-134: "The native mass spectrum of STARD2 showed that >95 % of the protein copurified with phospholipids, suggesting stronger binding to human than to E. coli 134 lipids (70 %)". An alternative explanation is that a larger fraction of STARD2 is folded in cellulo than in E. coli. Similarly, in the biochemical transfer assay, was the quality of the liposome assessed (lipidomics, DSL, etc)?

# Information on protein folding can be derived from the native mass spectra. As a general principle, protein unfolding results in substantially increased charge states, leading to a shift toward lower m/z values. The observed charge state distributions for all three proteins are consistent with folded globular proteins. STARD7 and STARD10 display similar charge state distributions, while STARD2 exhibits lower charge states, in line with its significantly lower molecular weight. Comparable charge states were observed for proteins expressed in *E. coli* and in human cells, with slight deviations due to differences in molecular weight.

An additional strong indication of correct folding is the detection of protein–lipid complexes. As these proteins are soluble, the observation of lipid binding in native MS requires an intact hydrophobic lipid binding pocket. If the proteins were unfolded, lipid binding would not be observed. For comparison, a mass spectrum of unfolded STARD10 showing markedly increased charge states and complete loss of lipid binding, is presented in Figure 2B of our recent preprint: https://doi.org/10.64898/2026.01.18.700185.

Further evidence for functional integrity is provided by the liposome-based lipid transfer assays, which demonstrate that all recombinant proteins, except for the STARD7 R189Q mutant, significantly enhance lipid transfer between liposomes. We are therefore confident that the higher lipid occupancy of STARD2 when expressed in human cells compared to bacterial cells is not related to differences in protein folding. Instead, we attribute the reduced lipid binding to the fact that *E. coli* does not synthesize phosphatidylcholine (PC), the endogenous ligand of STARD2 and most abundant phospholipid in human cells. *E. coli* membranes are predominantly composed of phosphatidylethanolamine (PE) and phosphatidylglycerol (PG). In the absence of PC, STARD2 can bind PE and PG, as evidenced by their copurification with the protein when expressed in *E. coli*. However, PE and PG are readily and completely displaced upon incubation with synthetic PC *in vitro* (Figure 1A), indicating a substantially higher affinity of STARD2 for PC. We have clarified in the revised manuscript that *E. coli* membranes do not contain PC, as this was not sufficiently emphasized in the previous version

Page 5:

*E. coli* membranes predominantly contain PE and PG but lack PC, the endogenous ligand of STARD2.[39] Accordingly, the co-purified lipids were identified as PE and PG species (**Figure S2a**), both of which have previously been shown to bind STARD2 in the absence of PC.[31]

Regarding the liposomes used in the lipid transfer assays, we did not assess their size distribution by dynamic light scattering (DLS), as this technique was not available to us. However, we applied a rigorous and well-established extrusion protocol using sequential polycarbonate membranes with pore sizes of 400, 200, and 100 nm. This procedure yielded clear solutions, which is widely regarded as an indicator of liposome diameters around or below 100 nm. The lipids used for liposome preparation were extracted from HEK293S cells and analyzed by direct-infusion MS (see Figure 4a). We did not determine the exact percentage of fluorescent lipids incorporated into the final liposomes. However, this does not affect the validity of our comparisons, as the same liposome batch was used across all experiments. Furthermore, we report fluorescence values normalized to the maximal fluorescence intensity of the respective liposome batch rather than absolute fluorescence values. Therefore, minor deviations from the intended ratio of incorporated fluorescent lipids do not affect the interpretation of the results.#

- The data analysis is inconsistent. There is little mention of statistics and reproducibility. The graphs in figures 1 and 2 are without axis nor units. We do not know what is being measured nor what the values are. The legends of the figures fail not explain the units of the axis (e.g. %, percentage of what?).

# All figures and corresponding captions have been revised to ensure accurate data representation and comprehensive descriptions, including appropriate axis labeling and statistical details. Additional information is provided under “Specific points” below.#

- The figures have been overly simplified and lack important information: the Y axis labels, the number of replicates, and the statistical test used are missing. Some colours (i.e. PC, PE, PG) are not explained. The visual appearance of the graphs and the legends mainly constitute a conclusion of the result rather than a description of the data. It is therefore difficult to properly follow what has been done without consulting the materials and methods section very often.

# We addressed this point by adding axis labels to the figures and providing more detailed descriptions of the data and statistical analyses in the corresponding figure captions. In addition, we clarified the color coding used to distinguish between lipid classes in Figure 1 and Figures S2 and S9. The revised figure captions and main text now clearly describe how processed data points were derived from the raw files.#

- Insufficient reference to supplementary figures: the authors often fail to mention which panel of the supplementary figure is being cited, for example on page 8, line 184.

# We have reviewed the entire manuscript to ensure that individual panels in the SI figures are referenced where appropriate.#

Specific points:

Figure 1: It is important to mention that the assay involves the use of detergent and that lipid uptake in this case involves the use of micelles (not membrane bilayers). The arrows between the gray boxes are misleading, as they give the impression of a sub-analysis. Figures 1d and S2 (lower panels) are identical.

# We revised Figure 1 to include y-axes, removed the arrows, and extended the figure caption to provide a clearer description of the data shown. The use of detergent micelles is now explicitly mentioned in both the main text and the Figure 1 caption. The duplicate image has been removed from Figure S2.#

Figure 2b, c: What are the y-axis of the histograms, is it pmol ? mol%? What are the error bars, from Methods, it seems that it is 3 recordings of the same sample in the MS.

# We have now clarified the axis units and derivation of error bars in Figure 2 (now Figure 3) and the figure caption. In the mass spectra, the y-axis represents relative peak intensity (%) normalized to the most intense peak. In the histograms in Figure 3b, the y-axis represents the relative abundance (%) of individual PC species in the $MS^2$ spectrum, normalized to the sum of intensities of all detected PC species. Error bars in the histograms are based on biological triplicates: proteins were purified and analyzed on separate days from three consecutive passage numbers of the same cell lines. We have also clarified this point in the Methods section.#

Figure 3: 3a) The triangles indicated on top of the bars are not clear – what do they represent - significance?

# We have revised Figure 3 (now Figure 4) to improve interpretability of the displayed data, including statistical analysis and meaning of the triangles (see also request by Reviewer 3). The triangles are intended to guide the reader to representative lipid species, highlighting that STARD7 overexpression is associated with the highest proportion of monounsaturated PC, STARD10 overexpression with di-unsaturated PC, and STARD2 overexpression with polyunsaturated PC. Their meaning is now clarified in the figure caption.

Page 13:

Colored triangles point to the condition with the highest abundance for individual PC species (monounsaturated PC in STARD7-, di-unsaturated PC in STARD10-, and polyunsaturated lipids in STARD2-overexpressing cells).#

Figure 6: For these experiments, several important information is missing and should be provided in the legend. Indeed, Fig 6b shows different curve profile than Fig. 6c, why? Could this be explained by the experimental setting? Are different amount of liposomes or protein used? What is the origin of the proteins used for each experiment: purified from bacterial or from HEK? It should be clearly stated in the figure legend.

# We have included all information relevant for the interpretation of Figure 6 in the revised figure and caption. The figure panels now clearly indicate which proteins were used in each experiment (derived from *E. coli* or HEK cells, before or after dephosphorylation). The caption has also been expanded to provide additional experimental details, including protein and liposome concentrations. As correctly noted by the reviewer, the concentrations of STARD2 and STARD10 used in Figure 6b were reduced to slow down lipid exchange and allow observation of the initial kinetics with similar slopes for all proteins (otherwise a plateau is reached after approximately 2 minutes for STARD2 and STARD10, as seen in Figure 6c).#

In the text, the authors are making statements about differences and similarities of initial transfer rates. However, no quantification of this specific parameter can be found. What are the initial transfer rates, how reproducible are they? And are the differences statistically significant? Without this information it is difficult to draw any conclusion. Along those lines, the authors describe some curves as “similar” (Fig6c: R->Q mutations of STARD2 and STARD10 only minimally affect the transfer) and others as “different” (Fig6b: PC 16:0-12:0 and PC 18:1-12:0 are differently transferred by STARD2 and STARD10). However, simple comparison of the graph is not sufficient to make such statements. Real kinetic parameter/values should be extracted and compared. Statistical significance should be provided.

# We agree with the reviewer that rigorous quantification of the observed differences, including extraction of kinetic parameters and assessment of statistical significance, was missing. We now report first-order rate constants for all conditions in Figure 6 and Figure S23, enabling direct

comparison of lipid transfer rates. Welch's t-test was used to compare the two conditions in each case. All differences were statistically significant ($p < 0.05$), except for the comparison between the STARD7 arginine-to-glutamine mutant and the liposomes only condition, indicating that the mutation completely abolishes lipid transfer activity. We have revised the manuscript to describe differences between conditions using quantitative terms (e.g., "twofold increase in the first-order rate constant") and added a section to the Methods describing the kinetic fitting procedure:

Page 31:

Fluorescence time courses were analyzed by nonlinear regression using a single-exponential model describing a first-order approach to equilibrium:

$I(t)=I_0+(I_\infty-I_0)(1-e^{-kt})$

where $I(t)$ is the relative fluorescence intensity at time $t$, $I_0$ is the initial relative fluorescence, $I_\infty$ is the asymptotic relative fluorescence at equilibrium, and $k$ is the apparent first-order rate constant. Each replicate was fitted independently using nonlinear least-squares regression implemented in Python (scipy.optimize.curve_fit). For each condition, rate constants were obtained from three independent measurements and are reported as mean ± standard deviation. Statistical differences between conditions were assessed using Welch's two-sample t-test applied to the fitted rate constants.#

Line 114 : "from STARD10-expressing cells bacteria" should be "from STARD10-expressing bacteria"

# We changed "cells" to "bacteria".#

Lines 133-134: "We characterized these ligands using multistage native MS and found that they were exclusively PC lipids". Why don't they see the other lipids they saw in vitro?

# We distinguish between three experimental scenarios: (i) lipids copurified with STARD2 following recombinant expression in *E. coli* (Figure 1a, left panel), (ii) lipids bound to STARD2 after recombinant expression in *E. coli* followed by *in vitro* incubation with a mixture of synthetic phospholipids (Figure 1a, right panel), and (iii) lipids copurified with STARD2 after expression in human cell lines (Figure 3b,c). All three experiments are fully consistent. As *E. coli* membranes do not contain PC, STARD2 copurifies with PE and PG, the only phospholipids available in scenario (i). However, upon incubation with a mixture of PC, PE, PG, PI, and PS *in vitro*, PC entirely replaces the initially bound PE and PG lipids, demonstrating that STARD2 preferentially and exclusively binds PC even in the presence of competing phospholipids (scenario ii). This behavior is mirrored in human cells (scenario iii). Because PC is present in human cells, STARD2 copurifies exclusively with PC despite the simultaneous presence of multiple other phospholipid classes including PE and PG. In summary, STARD2 is capable of binding phospholipids other than PC when PC is absent but binds exclusively to PC when PC is available.#

Line 207 : "The cellular lipidomes only exhibited modest changes in PC species, with the most prominent differences observed between the control and the three knockdown conditions (Figure S19)". Not clear what are these differences in S19.

# We have moved the data from Figure S19 to Figure 4b and revised the statement to make it more specific. Our original comment primarily referred to the clear difference between the control and knockout conditions for PC 34:1, which largely accounts for the lower levels of monounsaturated PC in the control condition. The text has been reformulated accordingly:

Page 13:

The cellular lipidomes showed only modest changes in PC species, with the most pronounced differences observed between the control and the three knockdown conditions for PC 34:1 (**Figure 4b**).#

**Reviewer #2 (Remarks to the Author)**

I co-reviewed this manuscript with one of the reviewers who provided the listed reports. This is part of the Nature Communications initiative to facilitate training in peer review and to provide appropriate recognition for Early Career Researchers who co-review manuscripts.

**Reviewer #3 (Remarks to the Author)**

Kirschbaum and colleagues present an interesting study elucidating the lipid selectivity profiles of three phospholipid transport proteins STARD2, STARD7 and STARD10. They utilise a multistage native mass spectrometry (MS) pipeline as the centrepiece of this work, which is complemented by molecular dynamics simulations, lipidomics, mutagenesis, and in vitro transport assays. Overall, the study is thorough, rigorous, and relatively well presented. I believe it is suitable for publication after the authors address my minor comments below.

- The presence of an acetate adduct on STARD7 is an obscure observation. The authors may wish to provide further clarification as to the origin of such an adduct and the reason for it.

# We agree with the reviewer that acetate adducts, or binding of volatile small molecules in general, are not typically observed in native MS, as such species usually evaporate during electrospray ionization or dissociate in the ion source. The fact that acetate remains associated with STARD7 therefore suggests that it is trapped within the lipid binding pocket, which was further supported by our mutagenesis experiment. This experiment demonstrated that acetate interacts with Arg189 in the STARD7 binding pocket in a 1:1 stoichiometry. Given its structural similarity to the fatty acid moieties of phospholipids, we hypothesize that acetate diffuses into the lipid-binding pocket and becomes stabilized through an electrostatic interaction with the arginine residue.

In the revised manuscript, we extended our analysis to additional native MS buffers and observed comparable 1:1 adduct formation with formate, indicating that this interaction is not specific to acetate (**Figure S3**). Moreover, we performed concentration-dependent measurements in which the ammonium acetate or formate concentration in the native MS buffer was systematically reduced. Under these conditions, adduct formation was markedly diminished. Taken together, these findings summarized in **Figure S3** strongly suggest that the observed acetate binding is not a physiological complex but rather an artifact of the native MS buffer conditions. We have extended our discussion on this unusual acetate adduct in the first two sections of the results accordingly. However, we cannot exclude the possibility that the ability of STARD7 to bind small carboxylates may have a biological function, although addressing this question is beyond the scope of the present manuscript. #

- The authors state that acetate may have displaced copurified lipids in STARD7. Are lipids observed prior to buffer exchange into ammonium acetate, for example by TLC?

# We thank the reviewer for this insightful suggestion. Following this recommendation, we extracted lipids from purified proteins expressed in *E. coli* using methyl tert-butyl ether (MTBE)/methanol extraction and analyzed the lipids by LC–MS/MS lipidomics. Equal amounts of each protein (100 µg) were used as starting material, and identical quantities of SPLASH Lipidomix internal standard (Avanti Research) were added to each sample. Detected lipids were quantified relative to PE 33:1-d7 and PG 33:1-d7 in the standard mixture. The quantitative lipidomics data, now presented in **Figure S4**, are fully consistent with the native MS results represented in Figure 1. STARD10 copurified with the highest amount of phospholipids, while STARD2 copurified with approximately half that amount, in agreement with the native mass spectra. In contrast, the lipid extract of STARD7 exhibited ~20-fold lower levels of copurified lipids. The signal was close to the detection limit and may largely reflect background contamination. As the analysis was performed without buffer exchange into ammonium acetate, these findings indicate that acetate does not displace bound lipids but that the lipid binding pocket is empty prior to buffer exchange, contrary to our previous hypothesis. Thermodynamically, this interpretation is also more plausible, as the displacement theory would require extraction of a

lipid from a hydrophobic binding pocket into the aqueous phase, which is energetically highly unfavorable. We have changed the text accordingly.

Page 5:

We therefore performed LC-MS/MS lipidomics on purified STARD2, STARD7, and STARD10 to quantify copurified lipids prior to buffer exchange. STARD7 copurified with an order of magnitude fewer phospholipids than STARD2 and STARD10 (**Figure S4**). We conclude that STARD7 has a relatively low affinity for phospholipids and is predominantly in its *apo* form when purified from *E. coli.* The absence of lipids allows carboxylates to enter the empty lipid binding pocket upon buffer exchange into native MS buffer. As acetate binding is not typically observed by native MS, the detection of 1:1 complexes with acetate could have biological relevance. However, any endogenously bound carboxylates would likely be lost during purification and thus remain undetected.#

- Could the authors provide an additional discussion of why STARD7 binds lipids with such low affinity in vitro?

# Across our different expression systems, we consistently observed that STARD7 copurified with substantially fewer lipids than STARD2 or STARD10 (no detectable lipid binding in *E. coli* and markedly lower binding of endogenous PC in human cell lines). This may indicate that a significant fraction of cellular STARD7 exists in the *apo* form in cells (or is bound to an alternative ligand, such as endogenous acetate as suggested by Reviewer 1), or that bound lipids are more readily exchanged and partially lost during protein purification. Supporting this interpretation, our MD simulations indicate that PC is more mobile within the STARD7 binding pocket than within those of STARD2 or STARD10, consistent with weaker binding and an increased propensity for lipid dissociation. However, based on the available data, we cannot definitively determine the underlying cause of the reduced lipid binding observed for STARD7.

In the *in vitro* experiment referenced by the reviewer (Figure 1b, right panel), 20 µM STARD7 was incubated with an equimolar mixture of PC, PE, PG, PI, and PS (100 µM total lipid). Accordingly, the final solution contained 10 µM protein and 10 µM PC, corresponding to a 1:1 STARD7:PC ratio. This relatively low effective PC concentration, together with the low apparent affinity of STARD7 for PC, may explain the limited binding detected under these conditions. We added a corresponding sentence to the text and refer to the following section discussing MD simulations of DOPC in the STARD7 binding pocket.

Page 6:

We attribute the reduced binding to a lower affinity of STARD7 for PC relative to STARD2 and STARD10, as further discussed in the following section.#

- The purity of STARD7 appears lower in both E. coli and mammalian cell expression systems. Are the higher molecular weight bands in the SDS-PAGE gels oligomeric species or contaminants? Could high contaminant levels be impacting copurification of lipids?

# We analyzed the individual bands observed in the SDS-PAGE gel of STARD7 expressed in HEK293S cells by excising each band, followed by in-gel digestion and proteomic analysis. Both the main band and the higher molecular weight bands yielded STARD7 as the primary hit, with additional detected peptides corresponding to common contaminants such as keratin. We propose that the main band at approximately 40 kDa corresponds to the signal peptide-cleaved STARD7 monomer, while the band above it represents the monomer with an uncleaved signal peptide. The higher molecular weight bands are consistent in mass with STARD7 dimeric and trimeric species. We were unable to assign the lower molecular weight band below the main band. Overall, however, the majority of bands could be assigned to distinct forms of STARD7 (signal peptide-cleaved or uncleaved, monomeric and oligomeric species). The bands are now annotated in Figure S7, and the corresponding proteomics datasets are

available via Figshare. We have replaced the gel shown in Figure S7 with the exact gel from which the bands were excised for proteomic analysis. All additional gel images from biological replicates are provided via Figshare.

In the native mass spectra, we exclusively observed signal peptide-cleaved STARD7 in the form of monomers and also frequently as dimers and higher oligomers, suggesting a higher intrinsic propensity for oligomerization compared to STARD2 and STARD10. No impurities were detected in the native MS measurements. Although the absence of detectable signals does not exclude the presence of contaminants, neither visible nor invisible impurities should influence lipid copurification. Exceptions would include specific cases, such as proteins that selectively stabilize the apo or lipid-bound form of STARD7. From a technical perspective, however, the presence of contaminants does not affect the apo:lipid-bound ratio observed in native MS. In summary, all higher molecular weight bands correspond to STARD7 species, and we do not anticipate any impact of potential impurities on lipid copurification.#

- It is stated that STARD7 is truncated – it would be helpful to indicate full length and truncated products in the intact spectra/zoomed insets.

# We expressed a truncated STARD7 construct (residues 76–370) in *E. coli* to account for signal peptide cleavage in human cells and now consistently indicate this throughout Sections 1 and 2, as well as in Figures 1, 2, and 6.

In human cell lines, we expressed the full-length construct and detected two signal peptide-cleaved proteoforms by MS: STARD7(79–370) (major) and STARD7(78–370) (minor). Because both proteoforms are present, we collectively label them "STARD7" in Figures 3 and 4 but indicate the presence of both in the caption of Figure 3.

Page 10:

The signal peptide of STARD7 is cleaved at two alternative sites, yielding predominantly STARD7(79–370) and a minor fraction of STARD7(78–370), which displays a mass shift of +70 Da.#

- It is stated that Fig 3 displays how expression of the three proteins alters lipid compositions. I can not interpret Fig 3a as drawn. Perhaps some labels are missing? What are the arrows? What are the statistical tests used (I presume that's what the *s denote). What 'relative quantitation' was performed. Presumably to a non-expressing control? More details and clarity are needed.

# We agree with the reviewer that the original Figure 3 (now Figure 4) and its caption did not provide sufficient detail to fully interpret the data. We have therefore revised the figure as follows: panel b has been replaced with the siRNA knockdown data to allow direct comparison between overexpression and knockdown conditions. The y-axes now explicitly indicate "% of total PC," meaning that each lipid species is normalized to the total PC detected in the same sample (referred to as relative abundance in the caption). The statistical tests used are now specified in the caption (ANOVA and PERMANOVA for comparisons between two or three groups, respectively), with additional details provided in the Methods section. The triangles are intended to guide the reader to representative lipid species, highlighting that STARD7 overexpression is associated with the highest proportion of monounsaturated PC, STARD10 overexpression with di-unsaturated PC, and STARD2 overexpression with polyunsaturated PC. Their meaning is now clarified in the figure caption

Page 13:

Colored triangles point to the condition with the highest abundance for individual PC species (monounsaturated PC in STARD7-, di-unsaturated PC in STARD10-, and polyunsaturated lipids in STARD2-overexpressing cells).#

- Figure 3b is repeated in Supp Figure S20. I would suggest removing this from Figure 3 (see next comment).

# We have removed all duplicate figures that were previously presented in both the main manuscript and the Supporting Information. Specifically, the reproduction of Figure 3a (now Figure 4a) has been removed from the Supporting Information. In addition, former Figure 3b has been removed from the main manuscript, and the corresponding expression level data are now presented exclusively in the Supporting Information (Figure S17).#

- The statements around tissue specific protein/lipid distributions are intriguing but underdeveloped and lack rigorous support. The authors conclusively draw a correlation by only considering two datapoints (liver and heart). Are additional data available of tissue specific lipid profiles to provide further evidence to support the authors claims? If not, the authors should tone down their statements about this data. Moreover, such expression data are from transcriptomics, which does not always correlate with protein levels. This caveat must be stated.

# We agree with the reviewer that the data supporting this claim are limited and have toned down our statements accordingly. We now explicitly state, both in the main text and in Figure S17, that protein expression levels are inferred from transcriptomic data. Unfortunately, comparable and rigorous lipidomics datasets across human tissues are not available, and data quality varies substantially between studies, limiting direct comparisons. We selected liver and heart because high-quality lipidomics datasets were available for these tissues and, coincidentally, STARD expression levels differ markedly between them. These caveats are now clearly highlighted in the main text.

Page 14:

It should be noted that these data are limited by the fact that protein expression levels are inferred from transcriptomic analyses rather than direct protein measurements, and that comparable high-quality lipidomics datasets across human tissues are currently lacking for broader analysis. Nonetheless, the currently available data lead us to hypothesize that the acyl-chain selective LTPs STARD2 and STARD10 may exhibit higher expression levels in tissues with higher levels of PC unsaturation.#

- Did the authors examine lipid binding in their phosphatase treated proteins? This might help to provide additional clarity to the interpretation of lipid transfer functional data.

# We thank the reviewer for this helpful suggestion. To examine lipid binding to phosphorylated vs. dephosphorylated STARD10, we performed new additional experiments using STARD10 expressed in HEK293S cells, which was previously used in the lipid transfer assay. Because delipidation of STARD10 was not feasible, we instead monitored exchange of endogenous bound lipids with synthetic lipids supplied by liposomes. To enable clear distinction between native and added lipids, we prepared liposomes containing deuterated PC (PC 16:0-d31/18:1), which is readily distinguishable by mass from endogenous PC species. STARD10, with or without λ-phosphatase treatment, was incubated with these liposomes, and lipid exchange was measured using native MS at three time points (10, 20, and 30 minutes). We quantified lipid exchange based on the relative intensity of deuterated PC detected in the $MS^2$ spectra of STARD10–phospholipid complexes. Consistent with the lipid transfer assay, phosphorylated STARD10 showed minimal exchange of endogenous PC for deuterated PC, indicating a strongly reduced ability to extract lipids from liposomes. In contrast, dephosphorylated STARD10 exhibited a clear time-dependent uptake of deuterated PC. These data are now included in **Figure 6e**, with representative mass spectra shown in **Figure S25**. Together, these results demonstrate that phosphorylation markedly impairs lipid uptake by STARD10, potentially by interfering with its association with lipid bilayers. As partial phosphorylation of STARD2 did not significantly affect its lipid transfer activity, those data have been moved to the Supporting Information (Figure S23). We have added a final section to the manuscript.

Page 17:

To relate phosphorylation-controlled lipid transfer activity to native MS measurements, we examined whether phosphorylation of STARD10 affects lipid uptake from liposomes. We chose a simplified liposome system containing deuterated PC 16:0-d31/18:1, which is readily distinguishable by mass from endogenous PC species. STARD10 expressed in HEK293S cells was incubated with these liposomes either in its native, phosphorylated state or following λ-phosphatase treatment. Native MS analysis revealed that phosphorylated STARD10 exhibited minimal exchange of endogenous PC for deuterated PC, whereas dephosphorylated STARD10 showed pronounced, time-dependent uptake of deuterated PC from liposomes (**Figure 6e, Figure S25**). These findings indicate that phosphorylation of the flexible STARD10 C-terminus impairs lipid uptake from liposomes, potentially by reducing membrane association or obstructing the lipid entry site. This experiment further demonstrates how native MS-based lipid binding measurements can be directly correlated with functional lipid transfer activity.#

- I found it difficult to navigate the supplementary. Whilst I understand the data are thematically organised, it would be better for the reader if this was sequentially arranged.

# In the revised Supplementary Information, the data are now presented in the order in which they appear in the main text.#

**Reviewer #4 (Remarks to the Author):**

The authors have used native mass spectrometry to investigate the lipid selectivity of three lipid transfer proteins: STARD2, STARD7 and STARD10. They report different lipid preferences for the three proteins and in particular their selectivity towards acyl chains: STARD2 copurifies with long polyunsaturated chains, STARD7 binds a wide range of lipids, and STARD10 copurifies preferentially with di-unsaturated phospholipids. This is also observed in in vitro lipid transfer assay. This work brings new and important information on the elusive acyl chain selectivity of three lipid transfer proteins and is in principle of interest for the readership of Nature Communications.

Unfortunately it does not succeed at proposing a structural explanation, despite extensive molecular dynamics simulations. For example a limitation of the simulations is that DOPC was used for all three proteins, despite the experiments showing that they have different acyl chain selectivity.

# Our choice to select DOPC as the ligand to simulate with all three proteins was driven by two main rationales. First, it is a common ligand for all three proteins. Second, using a single ligand enables direct comparison of interaction profiles across the different proteins. We have now carried out comparable additional simulations with the respective most abundant acyl chain substrate for each protein (SAPC for STARD2 and POPC for STARD7, results are shown in **Figure S6d** and **Figure S6e**) and confirmed our initial qualitative results that STARD7 engages its natural substrate with weaker interactions compared to STARD2 and STARD10.#

The molecular dynamics simulations were used to monitor the dynamics of a docked DOPC in the pockets of STARD2, 7 and 10 wild-type and of an arginine to glutamine mutant. The arginine is conserved and has been observed before to interact with a bound lipid in STARD2 (Titeca et al, Nature, 2026). The lipid bound to STARD7 is observed to be more mobile than the lipids bound to STARD2 and STARD10. The authors conclude that their simulation data "suggest that STARD7 has lower affinity for PC lipids compared to STARD2 and STARD10". The statement "has lower affinity for PC lipids" is not correct given the data shown and should be replaced by "engages in weaker interactions with DOPC compared to STARD2 and STARD10".

# We thank the reviewer for highlighting this and we apologize for this oversight. We have now rephrased the sentence correctly, mentioning that in the MD simulations DOPC engages in weaker

interactions with STARD7, compared to STARD2 and STARD10, based on the RMSF and protein-lipid distance analyses.#

The starting structures are built with Boltz-2 (a co-folding method) and shown on Figure 4C. MD simulation outcomes are in general dependent on input structures and the authors do not sufficiently document and discuss the differences between the DOPC-bound models of STARD2, 10 and 7 might and how these might have influenced the differential behavior of STARD7. The authors should provide information about the difference between their 3 models obtained with Boltz-2 and the lipid-protein complexes of STARD2, and the differences between the three models. One cannot see from Figure 4c if the three lipids have comparable poses, or if their headgroup is positioned in the cage as expected from the 3 STARD2 X-ray structures. The best would be to provide the three models as pdb or mmcif files as supporting information.

# The predicted models, solvated and ionized, are already available in the supporting raw files under the name "initial.pdb" for each protein under the corresponding filename. Further, we now added a Supplementary Figure (**Figure S6a,** also attached below), to better describe the binding pose of DOPC in all models with reference to the crystal structure of lipid-bound STARD2 (PDB-ID: 1LN1).

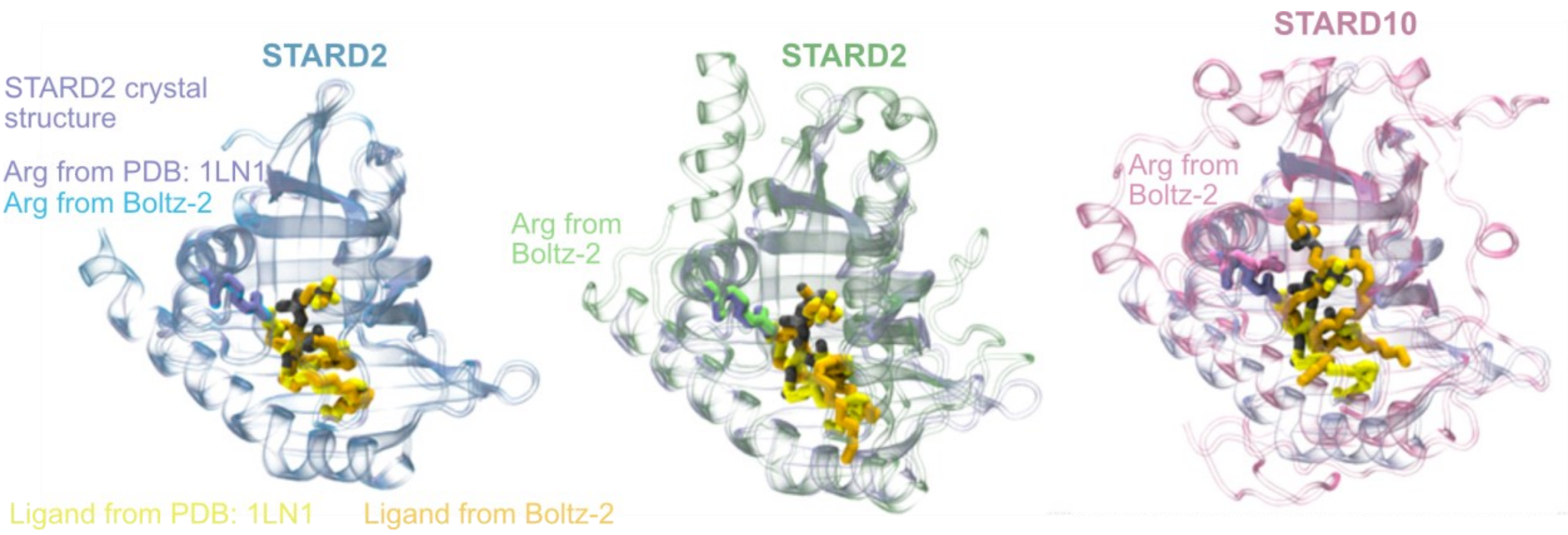


**Figure S6 a.** Binding poses of DOPC in Boltz-2-predicted models with reference to the crystal structure of lipid-bound STARD2 (PDB-ID: 1LN1). Lipid ligands and conserved arginine residues are highlighted.#

The RMSF (Figure 4d) of the DOPC lipid headgroup is fairly high for STARD7. Interestingly STARD2 and STARD7 have a conserved aromatic cage but the cage is absent from STARD10. One would therefore expect that the DOPC headgroup would be more loosely bound in STARD10 than in 2 or 7, but it does not seem to be the case. Is that correct? Please provide distances between the choline group and the aromatics of the cage.

# The attached figure below shows the aromatic cages of STARD2 and STARD7, showing comparable binding of the aromatic cage to the lipid headgroup in the two proteins.

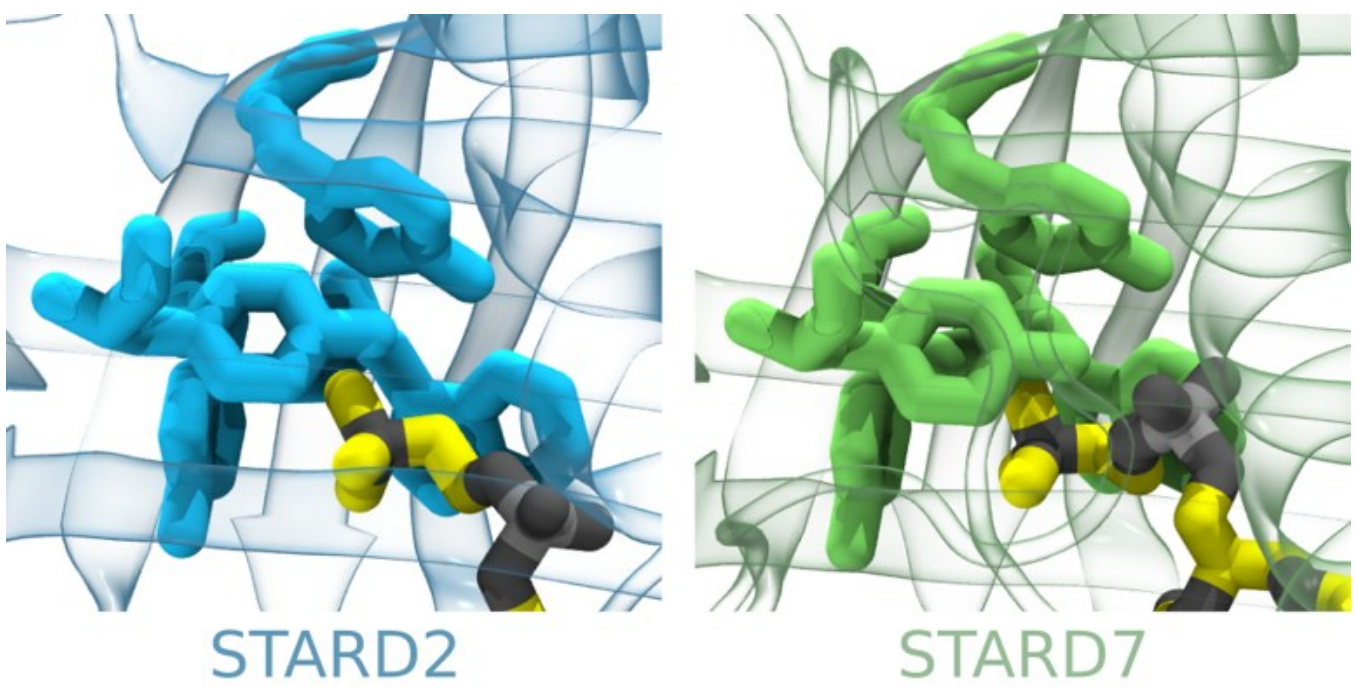

Analysis of the individual distances between the centre of mass of the choline group and the centre of mass of the aromatic cage are now shown in **Figure S6b** (also attached below). Interestingly, these data show that the choline group exhibits comparable binding to the aromatic cage in both wildtype STARD2 and STARD7. Upon mutation of the conserved arginine residue into glutamine, the binding of the choline group appears more weakened in STARD7 than in STARD2.

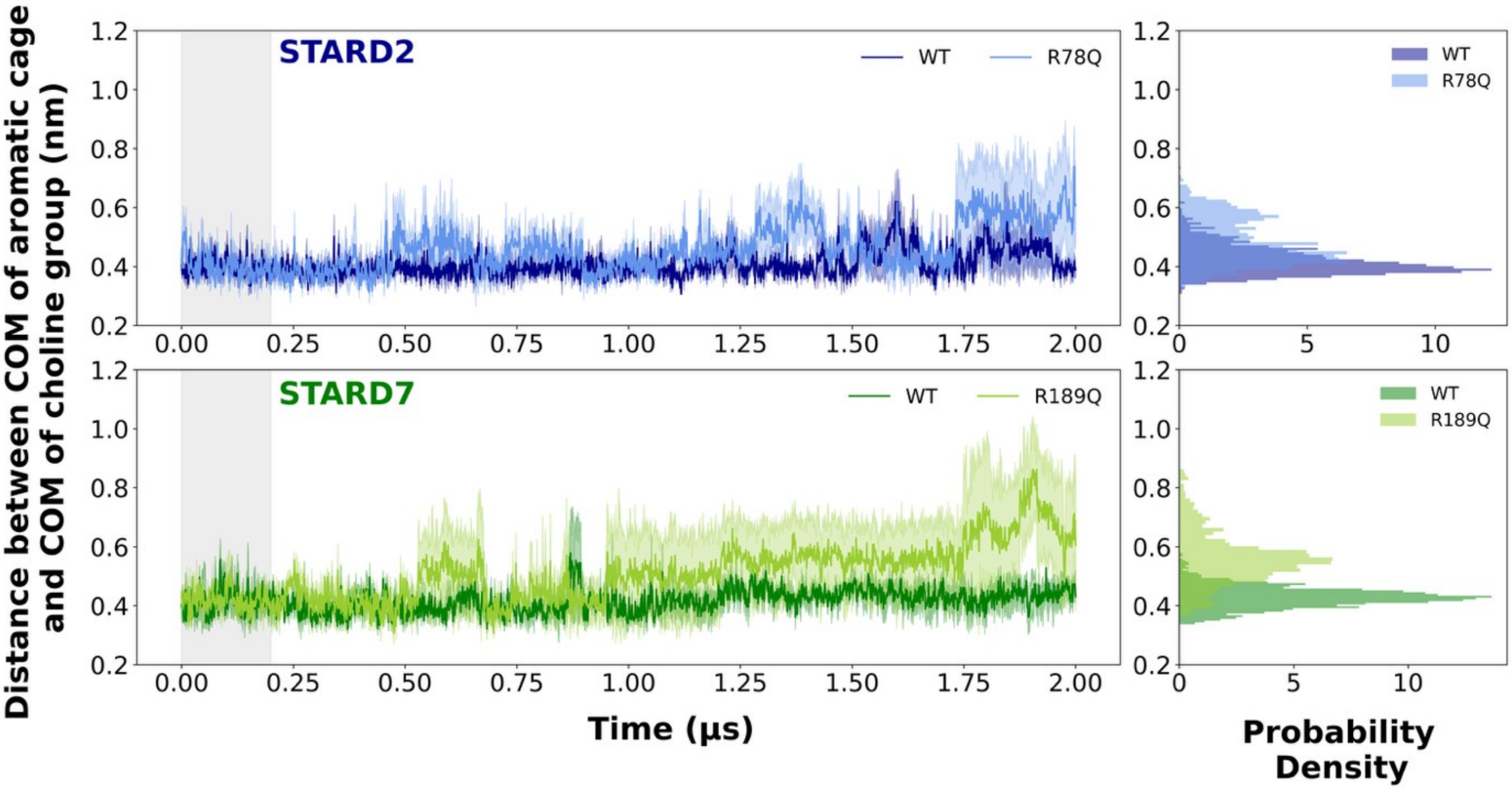


**Figure S6 b.** COM distances between the choline group of DOPC and the aromatic cage in the wild type proteins (dark traces) or the arginine-to-glutamine mutants (light traces). The shaded region around the solid line is the standard error of mean. The first 0.2 μs of simulation, shaded in grey, were excluded in the probability density calculation.#

**REVIEWER COMMENTS**

**Reviewer #1 (Remarks to the Author):**

The authors have carefully addressed our requests. The manuscript has improved in terms of readability. It is now much easier to navigate the complex data and the importance/relevance of the results are clearly demarcated. We find the approach interesting and powerful. The authors demonstrate how native spectrometry approaches can contribute to the understanding of LTP functioning in the cell. This should be of interest to a large community. As the experiments are complex and it may help the reader to further clarify a few points:

# We thank the reviewer for their positive feedback and address the remaining points below.#

- To avoid any confusion, it is best to use only the term “mitochondrial localisation signal” to refer to the 1-74 sequence of STARD7 (rather than the term “signal peptide”).

# We replaced the term “signal peptide” by “mitochondrial localization signal” throughout the revised manuscript and Supporting Information.#

- As mentioned the experiments are complex and Figure 1a,b,c could benefit from a rework to improve readability and understanding. On the left panels: i) we barely see the grey box around the selected m/z and it takes quite some time to link it to the grey insets. As this is an important figure it may deserve a larger size.

# In the revised Figure 1, we added arrows linking the grey box in the full spectrum to the corresponding insets, which we hope makes the connection between them clearer. We elongated the figure to accommodate the experimental scheme (see below) but we do not believe that the available data are sufficient to justify a double-column figure. Note that we also added arrows between the full spectra and insets in Figure 3a for consistency.#

ii) the grey insets differ in the presentation between the 3 proteins: the 9+, 11+ and 12+ are not always written at the same place, which is confusing. The position versus the x-axis (and explanation) of the schematic representation of the LTP (coloured (red/green/violet) cheese), is difficult to grasp. This should be clarified and/or explained in the legend.

# We placed the charge state in the same position for each protein (upper right corner) and removed the pie representations in the insets, as their localization should now be clear by the arrows connecting the full spectrum and inset. The filled pie symbol encompasses all peaks corresponding to lipid-bound protein shown in the insets. We labelled the mass shift of the most abundant bound lipid peak relative to the apo protein. This is now clarified in the caption.#

On the right panels:

iii) These panels represent different MS experiments than the ones presented on the left – and this is not obvious from the display. They imply the use of micelles solubilised by C8E4 to determine lipid class selectivity and this is still not evident from the display, i.e. without the legend. A scheme or text could be added in the figure to make it clearer. Or may be better, a schematic of the 2 workflows explaining the principle of these experiments would help a lot the non-expert.

# Following the reviewer’s suggestion, we extended the figure to include an experimental scheme (now Figure 1a), which is directly related to the mass spectra shown below in the left and right panels. We describe the composition of the mixed lipid/C8E4 micelles in Figure 1a and hope that the sample treatment is now sufficiently clear from the display, without referring to the caption.#

- Figure 2b. It is not clear to what lipids the R◊Q mutant proteins were exposed to. From the available information in the legend and main text, it seems that STARD2 and STARD10 data does not correspond to the same experiment as for STARD7. The text says STARD7 R189Q mutant is not binding to PC even when incubated with a large excess of DOPC - is it what is shown in panel 2b? However, the legend suggests that the MS measurements are from STARD7 exposed to bacterial lipids during expression. This requires clarification.

# We clarified the different experimental conditions in the text and figure caption. All three mutants were expressed in *E. coli*, and the lipids observed in the native mass spectra of STARD2 and STARD10 mutants represent copurified bacterial PE and PG. The STARD7 mutant did not copurify with any lipids like previously observed for wild-type STARD7. We therefore incubated the purified protein with 100 µM DOPC (micelles containing 0.5 % C8E4) because we knew from the previous experiments (Figure 1) that wild-type STARD7 is able to bind PC *in vitro* under these conditions. However, despite using pure DOPC micelles rather than mixed lipid micelles (i.e. the effective PC concentration being 5x higher than in Figure 1c), we observed no DOPC binding to the STARD7 mutant. We reformulated the text accordingly:

We expressed the three R→Q mutants STARD2 R78Q, STARD7(76–370) R189Q, and STARD10 R92Q in *E. coli* and quantified the percentage of LTP that copurified with lipids by native MS (Figure 2b, Figure S5). The deconvoluted native mass spectra of STARD2 R78Q and STARD10 R92Q show that the mutation of the arginine residue does not compromise phospholipid binding, as bacterial phospholipids copurified in similar abundance as with the wild-type proteins. STARD7(76–370) R189Q did not copurify with phospholipids, as previously observed for wild-type STARD7. However, unlike the wild-type protein, STARD7(76–370) R189Q did not bind PC after incubation with excess DOPC (1:5 protein:lipid ratio) in 0.5% C8E4, whereas the wild-type protein showed clear DOPC uptake under the same conditions. Furthermore, we did not observe acetate binding in the native mass spectrum of STARD7(76–370) R189Q.#

- Since STARD7 WT does not bind so much DOPC (Fig1b, right panel), a comparison of the difference of PC binding between STARD7 WT and R182Q in the same plot would strengthen the statement that STARD7 R182Q has impaired PC binding. At the moment, this is mostly supported by the loss of acetate binding. You can use the data of figure S5b.

# In the revised Figure 2, we overlaid the deconvoluted spectra of all wild-type proteins (gray) with the spectra of the mutants (black). It is now evident that the STARD7 mutant binds neither DOPC nor acetate, contrary to wild-type STARD7 under identical conditions. While Figure 2b now contains the same spectra as Figure S5, we decided to keep Figure S5 because it contains additional annotations and shows the spectra at a larger scale for closer inspection. Note that the peaks corresponding to the apo proteins are slightly shifted between the wild-type and mutant proteins because the mutation changes the intact protein mass.#

- The description of STARD10 phosphorylation is intriguing. On the one hand, 95% of STARD10 from HEK and HepG2 contains lipids and STARD10 is highly phosphorylated (“95 and 90% at H229-R272 and M276-H322, respectively”). On the other hand, the in vitro transfer assay shows reduced transfer of phosphorylated STARD10 compared to dephosphorylated, suggesting that phosphorylation prevents transfer. How do you explain that? Is it possible that in cells, phosphorylated STARD10 is stalled in a lipid-bound state that prevents release which would explain the high amount of holo-STARD10? Can native MS help figure out these differences? Also, testing what step of lipid transfer is affected by phosphorylation would be interesting. Is it the uptake? the release? The binding to membranes? We realize that this may not be trivial, but this should at least be discussed.

#We agree that reconciling these results requires further discussion. Phosphorylation may affect lipid uptake, lipid release, or both, for example by impairing protein-membrane association. In our liposome-based lipid exchange assay, lipid transfer between the liposome and the lipid-binding pocket of phosphorylated STARD10 is impaired compared to non-phosphorylated STARD10, which is consistent with defects in lipid uptake, release, or both. If lipid uptake were primarily affected, the observation that we detect almost exclusively lipid-bound, fully phosphorylated STARD10 upon purification from human cells could be explained by lipid loading occurring prior to phosphorylation. Conversely, the reviewer's suggestion that lipid release could be impaired is more consistent with our data.

In principle, native MS would allow to discriminate whether lipid uptake or release are affected if we were able to delipidate STARD10. This would allow to start the assay with an empty lipid-binding pocket to measure lipid uptake specifically. However, despite extensive efforts, we were unable to obtain lipid-free STARD10, even under stringent delipidation conditions (2% octyl glucoside), which did not measurably reduce bound lipids. Therefore, we cannot experimentally distinguish whether lipid uptake, release, or both are affected. We have added a discussion in the revised manuscript to reconcile the high level of lipid binding with complete phosphorylation of STARD10 in cells:

Phosphates introduce negative charges that may disrupt protein–membrane association and lipid exchange between the membrane and the lipid-binding pocket of LTPs. We showed by native MS that lipid transfer between liposomes and the lipid-binding pocket of STARD10 is strongly impaired by phosphorylation. However, these experiments did not reveal which step of the exchange process, i.e., protein–liposome association, lipid uptake, or lipid release, is specifically affected. Notably, STARD10 purified from human cells was entirely phosphorylated while being almost fully saturated with bound phospholipids, suggesting that phosphorylated STARD10 may still efficiently take up lipids, whereas lipid release could be impaired. Distinguishing between defects in lipid uptake versus lipid release by native MS would require completely delipidated STARD10, enabling direct measurement of lipid uptake without previous lipid release like in our lipid exchange assay. However, we were unable to delipidate STARD10, as stringent detergent washing with 2% octyl glucoside did not measurably reduce the fraction of lipid-bound protein. Together, our results confirm that phosphorylation impairs STARD10 lipid transfer activity, although the underlying mechanism and the step affected remain unresolved.#

- Figure 7 : The model is interesting but is currently not in total accordance with the data presented here. The suggestion that phosphorylation affect binding to membrane and lipid transfer requires confirmation/clarification for STARD10 (see comment above – and then STARD10 should be represented with a lipid in the binding pocket) and is not supported for STARD2 nor STARD7. The actual version of the manuscript does not allow to claim for a general role of phosphorylation to prevent lipid uptake/transfer by these three LTPs.

# We have revised Figure 7 to highlight phosphorylation as a potential regulatory mechanism for STARD10, but not STARD2 or STARD7. STARD10 is now depicted with two phosphates and a lipid in its binding pocket, consistent with our experimental observations. Furthermore, we have revised the figure caption to more generally state that phosphorylation reduces STARD10 lipid transfer activity (which we experimentally observed in the liposome assay), without specifying which step in the lipid exchange between the STARD10 binding pocket and the bulk membrane is affected.#

- On several occasion, the authors use the term "affinity" to describe in reference to the proportion of STARD that forms complex with a cargo. The authors did not measure affinities. In this context, affinity is likely not the only determining factor for the observed occupancy rate. STARD proteins must have an affinity for their cargo in order to remove them from membranes, but for the transfer to occur, they

must then lose affinity for them to release the lipid in the acceptor membrane. The observed occupancy rate could be explained by many ways: higher uptake, lower release, etc (see comment above on STARD10). “Affinity” should be rephrased.

# We have replaced all mentions of “affinity” with alternative descriptions, as highlighted in the revised manuscript. The only exception is the use of “affinity” in the context of affinity purification.#

Typo : Line 2 page 6 : check bracket positioning.

# We assume that the reviewer refers to “dioleoyl (DO)PC”. We now correctly write out all lipid names:

dioleoyl PC (DOPC), dioleoyl PE (DOPE), dioleoyl PG (DOPG), dioleoyl phosphatidylinositol (DOPI), and dioleoyl phosphatidylserine (DOPS) #

Figure 6 panel E : Write which STARD is analysed (STARD10).

# We added “STARD10” to the heading of Figure 6e.#

Page 12, line 23 : Pay attention to “(Figure 4a)” position in the sentence.

# We have removed the period preceding (Figure 4) that caused confusion in the sentence.#

**Reviewer #2 (Remarks to the Author):**

I co-reviewed this manuscript with one of the reviewers who provided the listed reports. This is part of the Nature Communications initiative to facilitate training in peer review and to provide appropriate recognition for Early Career Researchers who co-review manuscripts.

**Reviewer #3 (Remarks to the Author):**

The authors have comprehensively addressed all my comments in this revised manuscript, with significant new data included. The manuscript is certainly ready for publication in its present form.

**Reviewer #4 (Remarks to the Author):**

I wish to thank the authors for their answers and the additional data. Overall I find that, unfortunately, they have not provided enough controls and/or data to rule out the effect of their starting structures on the outcome of the simulations, thus weakening the significance of their conclusions. Since the simulations are available, I believe that it should not take much time for the authors to provide answers to my questions below. My overall impression is that the simulation data is not critical to explain or support the experimental findings which appear convincing enough. The authors should nonetheless avoid over-interpreting simulation data, which, as I see it, do not yet convincingly explain why the R to Q mutation only has an effect on STARD7.

# We thank the reviewer for appreciating that the overall findings are convincing. We agree that the simulation data critically depend on the initial starting structures, and to this end, we performed additional analyses as requested and carefully reviewed the manuscript to avoid over-interpretation of the simulation data.#

(1) From the authors: # Our choice to select DOPC as the ligand to simulate with all three proteins was driven by two main rationales. First, it is a common ligand for all three proteins. Second, using a single ligand enables direct comparison of interaction profiles across the different proteins. We have now carried out comparable additional simulations with the respective most abundant acyl chain substrate

for each protein (SAPC for STARD2 and POPC for STARD7, results are shown in Figure S6d and Figure S6e) and confirmed our initial qualitative results that STARD7 engages its natural substrate with weaker interactions compared to STARD2 and STARD10.#

I thank the authors for their answer and appreciate the addition of simulations of STARD2 with SAPC, a polyunsaturated lipid, and of STARD7 with POPC (monounsaturated lipid). Comparing Figure 2d and Supplementary Figure S6, it does not seem like SAPC and DOPC exhibit different mobilities in simulations with STARD2. It is unlike the experimental data showing that STARD2 preferentially binds to polyunsaturated lipids, and is actually a very interesting observation indicating that the STARD2 pocket might actually be able to host a larger variety of lipids than those observed experimentally. Would the authors comment on this?

# We thank the reviewer for their observation, which allows us to further clarify the experimental data on STARD2. First, while STARD2 does indeed show preference for PUFA lipids (Fig. 3d), our mass spectrometry data also indicates that STARD2 co-purifies with all PC lipids in the cellular lipidome (Pearson's r=0.77) (Figure 1d), showing binding to a large variety of chains in PC lipids, including monounsaturated ones (Figure 3c).
Second, and consistent with this information, the simulations show that both SAPC and DOPC are stable inside the pocket of STARD2, but with DOPC tails being slightly more mobile than SAPC ones (Figure R1 and new Figure S7d). Overall, given the limitations of the AA-MD simulations, we can't conclusively determine the molecular origin of the observed preference of STARD2 for PUFA lipids, but the MD results agree with the experimental data, indicating that although STARD2 has a preference towards polyunsaturated acyl chains (Figure 3d), it still binds, to a lower extent, lipids of several chain lengths and varying saturation depending on the lipid abundances and cell types (Figure 3b,c).

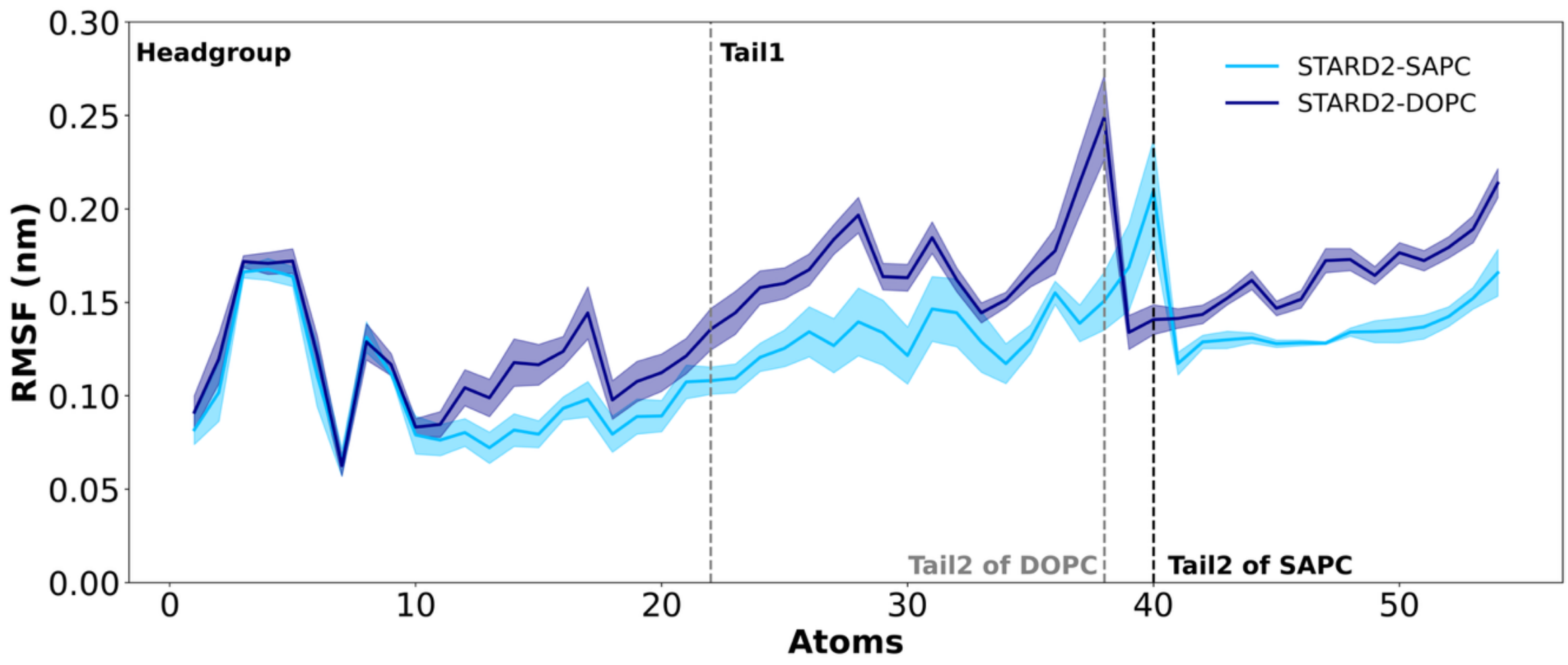


**Figure R1.** Root Mean Square Deviation (RMSF) of SAPC and DOPC inside the lipid-binding pocket of STARD2.#

(2) From the authors: # The predicted models, solvated and ionized, are already available in the supporting raw files under the name "initial.pdb" for each protein under the corresponding filename. Further, we now added a Supplementary Figure (Figure S6a, also attached below), to better describe the binding pose of DOPC in all models with reference to the crystal structure of lipid-bound STARD2 (PDB-ID: 1LN1).#

I appreciate that the authors provide all raw files and apologize if I missed them previously. In the Boltz-2 model for STARD10 with DOPC, the choline group is placed at a completely different site than for STARD2 and STARD7, where it naturally falls within the aromatic cage. STARD10 retains one of the four aromatic amino acids of the cage (Tyr117). Have the authors considered the possibility that the

choline could engage in cation-pi interactions with that tyrosine? Have different starting structures been tested?

# We now report, see answer below and new Supp Figure S7a, the time traces of the minimum distance between each aromatic residue of each protein and the choline N group of DOPC averaged over the three replicates. In STARD10, the cation-pi interaction between Tyr117 and the choline group of DOPC is indeed rather stable, showing a similar pattern to the interaction between DOPC and other aromatic residues in the conserved cages of the other proteins.

For what concerns the choice of the starting structure, we opted to use the same starting structure in the three replicates of each protein, with the only difference being the initial velocity distribution assigned to each replica, which follows standard molecular dynamics protocols (https://doi.org/10.1038/s41586-025-10040-y). The decision to begin with the same starting structure was strongly supported by the consensus among multiple tools (Boltz-2, Chai-1, and Openfold3) that predict essentially the same highest-scoring lipid-bound conformation. These models are now shown explicitly in new Supplementary Figure S6b and here Figure R2. Importantly, even lower-scoring models consistently positioned the lipid within the same binding pocket, with only minor variations in lipid tail orientation. Given this strong consensus across prediction tools and template conditions, we opted to only use one initial structure.

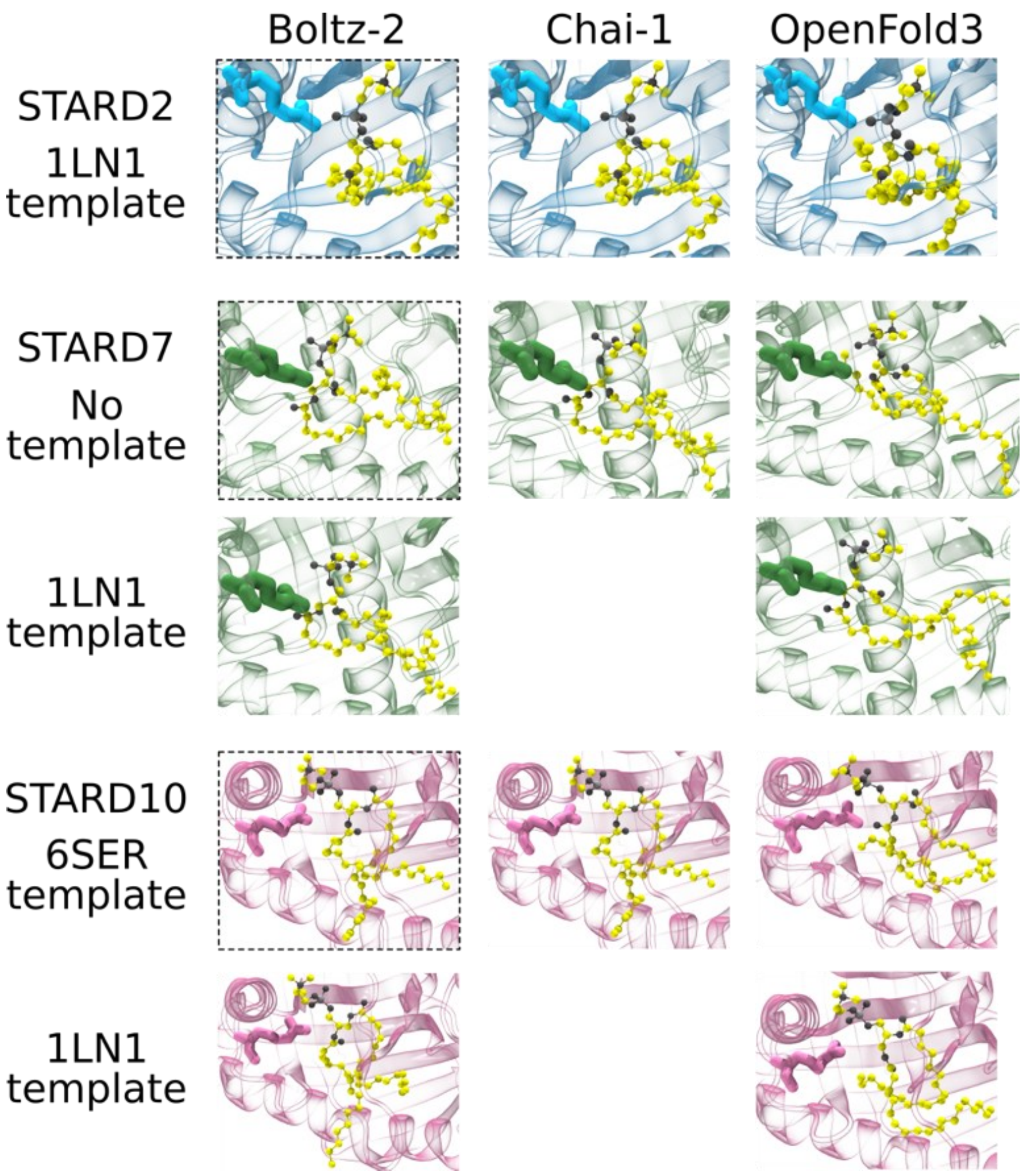


**Figure R2.** DOPC poses relative to the conserved arginine from different prediction tools for each protein with and without using a template to guide the prediction. The predictions selected for the simulations are highlighted in dashed boxes.#

In the starting structure for the simulations of STARD7 with DOPC (the only lipid for which I scrutinized the MD files) there is no hydrogen bond between the conserved arginine and the phosphate (also visible on Figure 2e). Can the authors rule out that this could be a cause to the lower occupancy of the phosphate-arginine weaker hydrogen bond (and weaker interactions with the aromatic cage)? What would happen if the lipid was positioned similarly to that starting structure of STARD2? As far as I understand from the Methods section, the replicates for each MD unfortunately use the same starting structure.

# Indeed, there is not a hydrogen bond between the conserved arginine and the phosphate in the STARD7 starting model. However, a similar placement of the ligand inside STARD7 was obtained when the crystal structure of STARD2 (PDB: 1LN1) was used as template (Figure S6b). Notably, also this predicted configuration positioned DOPC such that no hydrogen bond was formed between the phosphate group and the conserved arginine residue.

Additionally, while we can't rule out that the initial structure is not at the origin of such behaviour, we note that during the 2 µs-long unbiased all-atom MD simulations, which is generally sufficient for the ligand to spontaneously explore the cavity, there were instances where the DOPC headgroup closely associated with the conserved arginine (Figure 2, dark traces). However, these interactions were comparatively short-lived, and for most of the simulation time the ligand remained more loosely associated with the arginine, especially when compared with the more persistent interactions observed in STARD2 and STARD10 (Figure 2, dark traces).#

(3) From the authors: # The attached figure below shows the aromatic cages of STARD2 and STARD7, showing comparable binding of the aromatic cage to the lipid headgroup in the two proteins. Analysis of the individual distances between the centre of mass of the choline group and the centre of mass of the aromatic cage are now shown in Figure S6c (also attached below). Interestingly, these data show that the choline group exhibits comparable binding to the aromatic cage in both wildtype STARD2 and STARD7. Upon mutation of the conserved arginine residue into glutamine, the binding of the choline group appears more weakened in STARD7 than in STARD2.#

The authors seem to have misunderstood what analysis I asked about, namely the distance between the choline group (eg N atom, but COM of choline is ok) and each of the aromatic groups of the cage. The distance to the COM of the cage does not reflect if the cage remains in its expected structure around the choline, as observed in Xray structures of STARD2. Related to this question, the authors declare using the CHARMM force field but do not mention if they used the WYF correction for choline-aromatics cation-pi interactions. If this correction (or any equivalent) has not been applied, it is important to provide time series of either the cation-pi occupancies or of the choline-aromatics distances.

With the provided data, I see no support for the following sentences: "Interestingly, the choline group exhibits comparable binding to the aromatic cage in STARD2 and STARD7 (Figure S6b). Upon mutation of the conserved arginine residue into glutamine, however, the binding of the choline group appeared more weakened in STARD7 than in STARD2."

# We apologize to have misunderstood the analysis before; we have now updated it and included it as Supplementary Figure S7a:

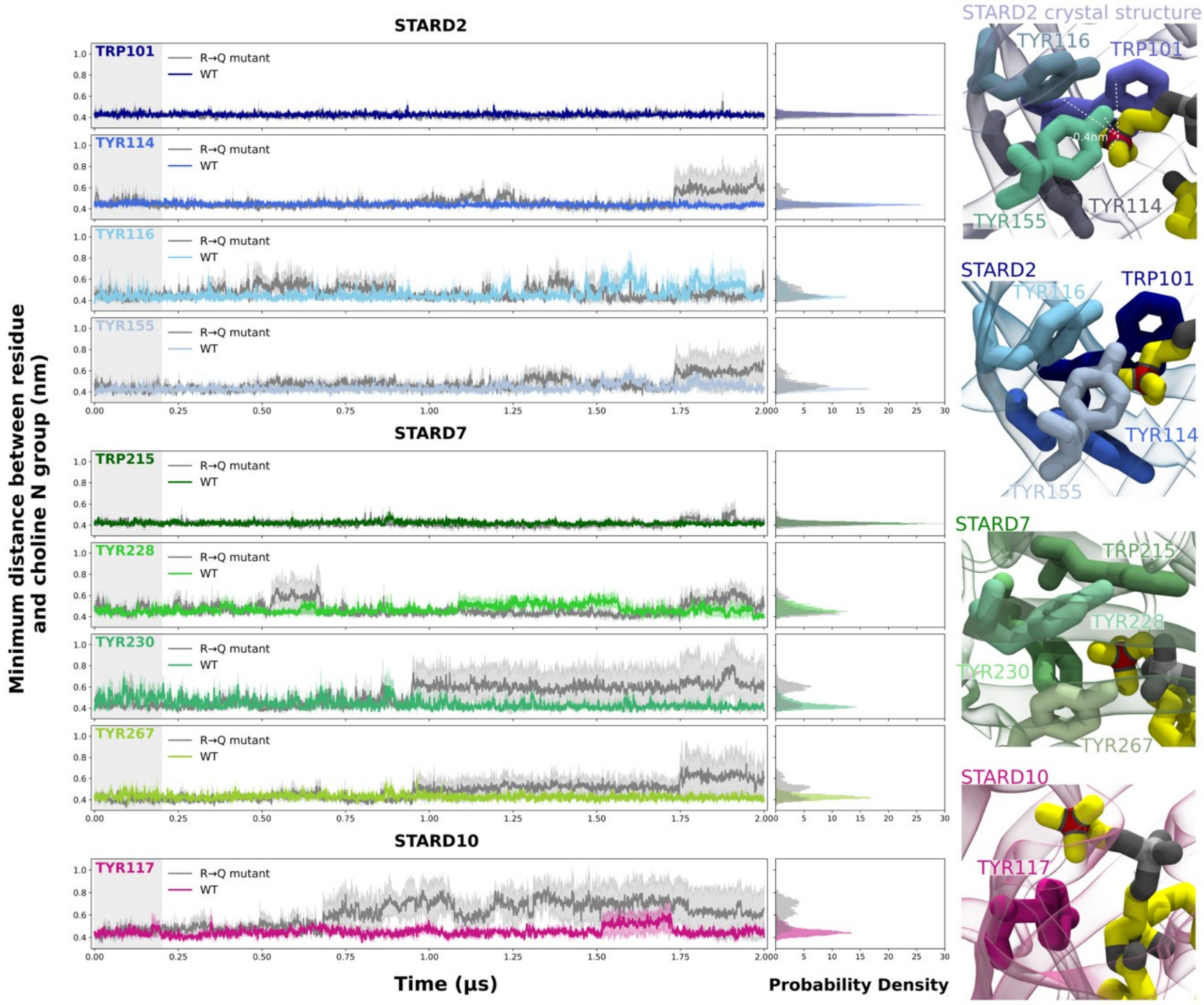


**Figure R3.** Right: Boltz-2 predictions of the DOPC headgroup (choline N group highlighted in red) interacting with the aromatic residues from the crystal structure of STARD2 (PDB-ID: 1LN1) and in each protein. Left: Minimum distance between the choline N group of DOPC and each aromatic residue of the conserved cages in STARD2 and STARD7 and Tyr117 in STARD10 in WT (dark colored traces) and the R→Q mutant (dark grey traces). The shaded region around the solid line is the standard error of mean. The first 0.2 μs of simulation, shaded in gray, were excluded in the probability density calculation.

In all cases, we did not use the WYF correction for choline-aromatics cation-pi interactions. In wild type simulations, the aromatic cage in STARD2 and STARD7 seems to retain its structure around the choline group throughout the simulations (Figure S7a,b). In the R→Q mutants the interactions between the choline group and the aromatic residues appear to be mostly destabilized in STARD7 (Y230 and Y267), as well as for the lone aromatic in STARD10, than in STARD2 (Figure S7a). We hope that the reviewer agrees that with the new analysis, albeit without the WYF correction, the modified sentence: "Interestingly, while the choline group exhibits comparable binding to the aromatic cage in STARD2 and STARD7 (Figure S7b), upon mutation of the conserved arginine residue into glutamine, the binding of the choline group with the aromatic cage appeared more weakened in STARD7 than in STARD2 (Figure S7a,b)." is now more supported by the data.#

**REVIEWER COMMENTS**

**Reviewer #4 (Remarks to the Author):**

The authors have satisfactorily answered most of my questions and comments. Yet, in one case (position of lipid in STARD10), the corresponding statements in the manuscript do not reflect the results presented, nor the answers that the authors provided in the rebuttal.

I acknowledge that the choice of the model for lipid-bound STARD10 is supported by several prediction tools. This model significantly differs from lipid-bound structures of STARD2 (eg. 1LN1 pdb id) and from the model for STARD7. The authors also confirmed in the rebuttal that the lipid choline headgroup in STARD10 is not positioned at the same place as in STARD2/7. All of this is fine but I suggest that the authors state the difference clearly in the text and correct the sentence starting at line 180. As it stands now, the text is at best misleading:

“As starting models for the simulations, we first obtained structural models for DOPC-bound STARD2, STARD7(76–370), and STARD10, using Boltz-240 (Figure 2c). The predicted binding poses are consistent with the location and orientation of DOPC in the crystal structure of STARD2 (Figure S6a), ...”

#We apologize for the oversight. We have now amended the sentence to:

“For STARD2 and STARD7, the predicted binding poses are consistent with the location and orientation of DOPC in the crystal structure of STARD2, while for STARD10 the DOPC choline group adopts a different orientation (**Figure S6a)**, likely due to the absence of the aromatic cage in STARD10.”#

Likewise, the sentence starting l.188 needs to be corrected as it reads as a statement valid for the three proteins given the sentence prior to it:

“Similarly, the interaction of the DOPC headgroup with aromatic residues inside the binding cavity remained stable throughout the simulations (Figure S7a,b)." This statement is only valid for STARD2 and STARD7.

#We apologize for the oversight. We have now amended the sentence to:

“Similarly, the interaction of the DOPC headgroup with aromatic residues inside the binding cavity remained stable throughout the simulations for both STARD2 and STARD7 (**Figure S7a,b**).”#

Finally, and related to the above: contrary to what is stated in the main text and in the rebuttal, the data on Fig.S7a does not support a cation-pi interaction between Tyr117 in STARD10 and the lipid choline headgroup. The snapshot does not indicate that the cation is stacked on either side of the ring, and the minimum distance between the (whole?) residue and the choline nitrogen seems like the wrong readout. The structure of cation-pi interactions is fairly basic structural biology (see for example 10.1126/science.271.5246.163, or Minoux and Chipot, JACS, 1999). To my eyes it actually looks as if there is no cation-pi interactions between the two groups in STARD10.

Please correct the text.

#Thanks for the observation. Indeed, careful analysis show no cation-pi interaction between Tyr117 and the lipid choline headgroup in STARD10, in agreement with the image depicted in Fig.S7a. To clarify

this, we have now removed any reference to STARD10 in Fig.S7a, which focuses on the aromatic cage, and accordingly, removed the following sentence from the main manuscript:

"Of note, the binding of the choline headgroup to Tyr117 of STARD10 was similarly weakened following the R→Q mutation (**Figure S7a**)."#
